# Exercise/physical activity and health outcomes: an overview of Cochrane systematic reviews

**DOI:** 10.1186/s12889-020-09855-3

**Published:** 2020-11-16

**Authors:** Pawel Posadzki, Dawid Pieper, Ram Bajpai, Hubert Makaruk, Nadja Könsgen, Annika Lena Neuhaus, Monika Semwal

**Affiliations:** 1 Kleijnen Systematic Reviews Ltd., York, UK; 2 Nanyang Technological University, Singapore, Singapore; 3 Institute for Research in Operative Medicine, Witten/Herdecke University, Witten, Germany; 4 School of Medicine, Keele University, Staffordshire, UK; 5 Jozef Pilsudski University of Physical Education in Warsaw, Faculty Physical Education and Health, Biala Podlaska, Poland; 6 Health Outcomes Division, University of Texas at Austin College of Pharmacy, Austin, USA

**Keywords:** Exercise, Health, Effectiveness, Mortality

## Abstract

**Background:**

Sedentary lifestyle is a major risk factor for noncommunicable diseases such as cardiovascular diseases, cancer and diabetes. It has been estimated that approximately 3.2 million deaths each year are attributable to insufficient levels of physical activity. We evaluated the available evidence from Cochrane systematic reviews (CSRs) on the effectiveness of exercise/physical activity for various health outcomes.

**Methods:**

Overview and meta-analysis. The Cochrane Library was searched from 01.01.2000 to issue 1, 2019. No language restrictions were imposed. Only CSRs of randomised controlled trials (RCTs) were included. Both healthy individuals, those at risk of a disease, and medically compromised patients of any age and gender were eligible. We evaluated any type of exercise or physical activity interventions; against any types of controls; and measuring any type of health-related outcome measures. The AMSTAR-2 tool for assessing the methodological quality of the included studies was utilised.

**Results:**

Hundred and fifty CSRs met the inclusion criteria. There were 54 different conditions. Majority of CSRs were of high methodological quality. Hundred and thirty CSRs employed meta-analytic techniques and 20 did not. Limitations for studies were the most common reasons for downgrading the quality of the evidence. Based on 10 CSRs and 187 RCTs with 27,671 participants, there was a 13% reduction in mortality rates risk ratio (RR) 0.87 [95% confidence intervals (CI) 0.78 to 0.96]; *I*^2^ = 26.6%, [prediction interval (PI) 0.70, 1.07], median effect size (MES) = 0.93 [interquartile range (IQR) 0.81, 1.00]. Data from 15 CSRs and 408 RCTs with 32,984 participants showed a small improvement in quality of life (QOL) standardised mean difference (SMD) 0.18 [95% CI 0.08, 0.28]; *I*^2^ = 74.3%; PI -0.18, 0.53], MES = 0.20 [IQR 0.07, 0.39]. Subgroup analyses by the type of condition showed that the magnitude of effect size was the largest among patients with mental health conditions.

**Conclusion:**

There is a plethora of CSRs evaluating the effectiveness of physical activity/exercise. The evidence suggests that physical activity/exercise reduces mortality rates and improves QOL with minimal or no safety concerns.

**Trial registration:**

Registered in PROSPERO (CRD42019120295) on 10th January 2019.

**Supplementary Information:**

The online version contains supplementary material available at 10.1186/s12889-020-09855-3.

## Background

The World Health Organization (WHO) defines physical activity “as any bodily movement produced by skeletal muscles that requires energy expenditure” [1]. Therefore, physical activity is not only limited to sports but also includes walking, running, swimming, gymnastics, dance, ball games, and martial arts, for example. In the last years, several organizations have published or updated their guidelines on physical activity. For example, the Physical Activity Guidelines for Americans, 2nd edition, provides information and guidance on the types and amounts of physical activity that provide substantial health benefits [2]. The evidence about the health benefits of regular physical activity is well established and so are the risks of sedentary behaviour [2]. Exercise is dose dependent, meaning that people who achieve cumulative levels several times higher than the current recommended minimum level have a significant reduction in the risk of breast cancer, colon cancer, diabetes, ischemic heart disease, and ischemic stroke events [3]. Benefits of physical activity have been reported for numerous outcomes such as mortality [4, 5], cognitive and physical decline [5–7], glycaemic control [8, 9], pain and disability [10, 11], muscle and bone strength [12], depressive symptoms [13], and functional mobility and well-being [14, 15]. Overall benefits of exercise apply to all bodily systems including immunological [16], musculoskeletal [17], respiratory [18], and hormonal [19]. Specifically for the cardiovascular system, exercise increases fatty acid oxidation, cardiac output, vascular smooth muscle relaxation, endothelial nitric oxide synthase expression and nitric oxide availability, improves plasma lipid profiles [15] while at the same time reducing resting heart rate and blood pressure, aortic valve calcification, and vascular resistance [20].

However, the degree of all the above-highlighted benefits vary considerably depending on individual fitness levels, types of populations, age groups and the intensity of different physical activities/exercises [21]. The majority of guidelines in different countries recommend a goal of 150 min/week of moderate-intensity aerobic physical activity (or equivalent of 75 min of vigorous-intensity) [22] with differences for cardiovascular disease [23] or obesity prevention [24] or age groups [25].

There is a plethora of systematic reviews published by the Cochrane Library critically evaluating the effectiveness of physical activity/exercise for various health outcomes. Cochrane systematic reviews (CSRs) are known to be a source of high-quality evidence. Thus, it is not only timely but relevant to evaluate the current knowledge, and determine the quality of the evidence-base, and the magnitude of the effect sizes given the negative lifestyle changes and rising physical inactivity-related burden of diseases. This overview will identify the breadth and scope to which CSRs have appraised the evidence for exercise on health outcomes; and this will help in directing future guidelines and identifying current gaps in the literature.

The objectives of this research were to a. answer the following research questions: in children, adolescents and adults (both healthy and medically compromised) what are the effects (and adverse effects) of exercise/physical activity in improving various health outcomes (e.g., pain, function, quality of life) reported in CSRs; b. estimate the magnitude of the effects by pooling the results quantitatively; c. evaluate the strength and quality of the existing evidence; and d. create recommendations for future researchers, patients, and clinicians.

## Methods

Our overview was registered with PROSPERO (CRD42019120295) on 10th January 2019. The Cochrane Handbook for Systematic Reviews of interventions and Preferred Reporting Items for Overviews of Reviews were adhered to while writing and reporting this overview [26, 27].

### Search strategy and selection criteria

We followed the practical guidance for conducting overviews of reviews of health care interventions [28] and searched the Cochrane Database of Systematic Reviews (CDSR), 2019, Issue 1, on the Cochrane Library for relevant papers using the search strategy: (health) and (exercise or activity or physical). The decision to seek CSRs only was based on three main aspects. First, high quality (CSRs are considered to be the ‘gold methodological standard’) [29–31]. Second, data saturation (enough high-quality evidence to reach meaningful conclusions based on CSRs only). Third, including non-CSRs would have heavily increased the issue of overlapping reviews (also affecting data robustness and credibility of conclusions). One reviewer carried out the searches. The study screening and selection process were performed independently by two reviewers. We imported all identified references into reference manager software EndNote (X8). Any disagreements were resolved by discussion between the authors with third overview author acting as an arbiter, if necessary.

We included CSRs of randomised controlled trials (RCTs) involving both healthy individuals and medically compromised patients of any age and gender. Only CSRs assessing exercise or physical activity as a stand-alone intervention were included. This included interventions that could initially be taught by a professional or involve ongoing supervision (the WHO definition). Complex interventions e.g., assessing both exercise/physical activity and behavioural changes were excluded if the health effects of the interventions could not have been attributed to exercise distinctly.

Any types of controls were admissible. Reviews evaluating any type of health-related outcome measures were deemed eligible. However, we excluded protocols or/and CSRs that have been withdrawn from the Cochrane Library as well as reviews with no included studies.

### Data analysis

Three authors (HM, ALN, NK) independently extracted relevant information from all the included studies using a custom-made data collection form. The methodological quality of SRs included was independently evaluated by same reviewers using the AMSTAR-2 tool [32]. Any disagreements on data extraction or CSR quality were resolved by discussion. The entire dataset was validated by three authors (PP, MS, DP) and any discrepant opinions were settled through discussions.

The results of CSRs are presented in a narrative fashion using descriptive tables. Where feasible, we presented outcome measures across CSRs. Data from the subset of homogeneous outcomes were pooled quantitatively using the approach previously described by Bellou et al. and Posadzki et al. [33, 34]. For mortality and quality of life (QOL) outcomes, the number of participants and RCTs involved in the meta-analysis, summary effect sizes [with 95% confidence intervals (CI)] using random-effects model were calculated. For binary outcomes, we considered relative risks (RRs) as surrogate measures of the corresponding odds ratio (OR) or risk ratio/hazard ratio (HR). To stabilise the variance and normalise the distributions, we transformed RRs into their natural logarithms before pooling the data (a variation was allowed, however, it did not change interpretation of results) [35]. The standard error (SE) of the natural logarithm of RR was derived from the corresponding CIs, which was either provided in the study or calculated with standard formulas [36]. Binary outcomes reported as risk difference (RD) were also meta-analysed if two more estimates were available. For continuous outcomes, we only meta-analysed estimates that were available as standardised mean difference (SMD), and estimates reported with mean differences (MD) for QOL were presented separately in a supplementary Table 9. To estimate the overall effect size, each study was weighted by the reciprocal of its variance. Random-effects meta-analysis, using DerSimonian and Laird method [37] was applied to individual CSR estimates to obtain a pooled summary estimate for RR or SMD. The 95% prediction interval (PI) was also calculated (where ≥3 studies were available), which further accounts for between-study heterogeneity and estimates the uncertainty around the effect that would be anticipated in a new study evaluating that same association. *I*-squared statistic was used to measure between study heterogeneity; and its various thresholds (small, substantial and considerable) were interpreted considering the size and direction of effects and the *p*-value from Cochran’s Q test (*p* < 0.1 considered as significance) [38]. Wherever possible, we calculated the median effect size (with interquartile range [IQR]) of each CSR to interpret the direction and magnitude of the effect size. Sub-group analyses are planned for type and intensity of the intervention; age group; gender; type and/or severity of the condition, risk of bias in RCTs, and the overall quality of the evidence (Grading of Recommendations Assessment, Development and Evaluation (GRADE) criteria). To assess overlap we calculated the corrected covered area (CCA) [39]. All statistical analyses were conducted on Stata statistical software version 15.2 (StataCorp LLC, College Station, Texas, USA).

## Results

**Fig. 1:**
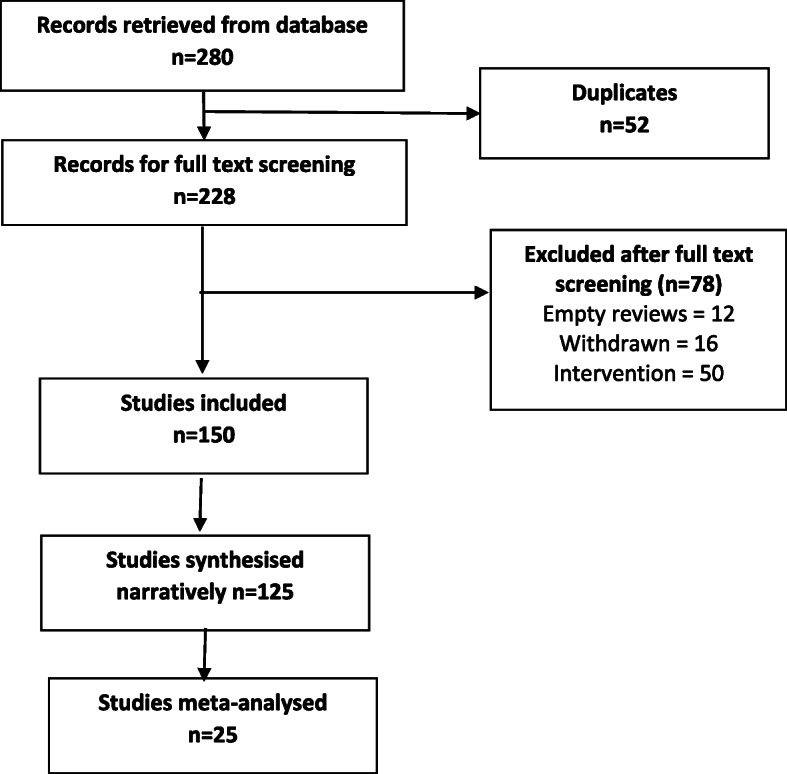
Study selection process

The searches generated 280 potentially relevant CRSs. After removing of duplicates and screening, a total of 150 CSRs met our eligibility criteria [40–189] (Fig. 1). Reviews were published between September 2002 and December 2018. A total of 130 CSRs employed meta-analytic techniques and 20 did not. The total number of RCTs in the CSRs amounted to 2888; with 485,110 participants (mean = 3234, SD = 13,272). The age ranged from 3 to 87 and gender distribution was inestimable. The main characteristics of included reviews are summarised in supplementary Table 1. Supplementary Table 2 summarises the effects of physical activity/exercise on health outcomes. Conclusions from CSRs are listed in supplementary Table 3. Adverse effects are listed in supplementary Table 4. Supplementary Table 5 presents summary of withdrawals/non-adherence. The methodological quality of CSRs is presented in supplementary Table 6. Supplementary Table 7 summarises studies assessed at low risk of bias (by the authors of CSRs). GRADE-ings of the review’s main comparison are listed in supplementary Table 8.

There were 54 separate populations/conditions, considerable range of interventions and comparators, co-interventions, and outcome measures. For detailed description of interventions, please refer to the supplementary tables. Most commonly measured outcomes were - function 112 (75%), QOL 83 (55%), AEs 70 (47%), pain 41 (27%), mortality 28 (19%), strength 30 (20%), costs 47 (31%), disability 14 (9%), and mental health in 35 (23%) CSRs.

There was a 13% reduction in mortality rates risk ratio (RR) 0.87 [95% CI 0.78 to 0.96]; *I*^2^ = 26.6%, [PI 0.70, 1.07], median effect size (MES) = 0.93 [interquartile range (IQR) 0.81, 1.00]; 10 CSRs, 187 RCTs, 27,671 participants) following exercise when compared with various controls (Table 1). This reduction was smaller in ‘other groups’ of patients when compared to cardiovascular diseases (CVD) patients - RR 0.97 [95% CI 0.65, 1.45] versus 0.85 [0.76, 0.96] respectively. The effects of exercise were not intensity or frequency dependent. Sessions more than 3 times per week exerted a smaller reduction in mortality as compared with sessions of less than 3 times per week RR 0.87 [95% CI 0.78, 0.98] versus 0.63 [0.39, 1.00]. Subgroup analyses by risk of bias (ROB) in RCTs showed that RCTs at low ROB exerted smaller reductions in mortality when compared to RCTs at an unclear or high ROB, RR 0.90 [95% CI 0.78, 1.02] versus 0.72 [0.42, 1.22] versus 0.86 [0.69, 1.06] respectively. CSRs with moderate quality of evidence (GRADE), showed slightly smaller reductions in mortality when compared with CSRs that relied on very low to low quality evidence RR 0.88 [95% CI 0.79, 0.98] versus 0.70 [0.47, 1.04].

**Table 1:** Quantitative evidence synthesis for mortality outcomes

Subgroup categories	No of SRs in MA	No of original studies in MA	No of participants in MA	Random-effects pooled RR [95% CI]	I-squared [%]	Prediction interval	Median effect size [IQR]
**Disease type**							
CVD	8	166	24,275	0.85 [0.76, 0.96]	38.5	0.65, 1.12	0.93 [0.74, 1.00]
Others	2	21	3395	0.97 [0.65, 1.45]	0.0	–	1.03 [0.93, 1.12]
**Type of exercise**							
Aerobic exercise	2	70	15,067	0.85 [0.67, 1.08]	77.9	0.07, 11.13	0.96 [0.74, 1.01]
Multiple exercise	3	20	1265	0.99 [0.21, 4.61]	0.0	0.00, 21,076.80	1.00 [0.40, 4.46]
Other exercise	3	52	4022	0.75 [0.51, 1.10]	26.4	0.03, 20.65	0.92 [0.54, 0.93]
Not reported	2	45	7317	0.90 [0.78, 1.03]	0.0	0.37, 2.15	0.93 [0.88, 1.12]
**Duration of intervention**							
Up to 12 months	4	27	1845	1.00 [0.32, 3.15]	0.00	0.08, 12.41	1.00 [0.70, 2.73]
More than 12 months	4	140	23,638	0.88 [0.78, 0.99]	47.1	0.65, 1.18	0.93 [0.88, 0.96]
Not reported	2	20	2187	0.72 [0.42, 1.22]	59.5	–	0.74 [0.54, 0.93]
**Session length**							
Up to 60 mins	3	25	2212	0.56 [0.35, 0.91]	0.0	0.02, 12.98	0.54 [0.40, 4.46]
More than 60 mins	5	141	22,063	0.88 [0.79, 0.97]	34.7	0.69, 1.11	0.96 [0.92, 1.00]
Not reported	2	21	3395				1.03 [0.93, 1.12]
**Frequency per week**							
Up to 3 times	2	43	3204	0.63 [0.39, 1.00]	9.4	–	0.73 [0.54, 0.92]
More than 3 times	5	121	20,887	0.87 [0.78, 0.98]	37.8	0.68, 1.13	0.93 [0.88, 1.00]
Not reported	3	23	3543	1.00 [0.67, 1.48]	0.0	0.08, 12.81	1.03 [0.93, 1.12]
**Evidence quality**							
Very low to low	6	50	5791	0.70 [0.47, 1.04]	0.0	0.40, 1.23	1.00 [0.54, 1.12]
Moderate	4	137	21,879	0.88 [0.79, 0.98]	45.6	0.66, 1.17	0.93 [0.88, 0.93]
**Risk of bias (RoB)**							
Low	3	77	9152	0.90 [0.78, 1.02]	0.0	0.67, 1.20	0.93 [0.90, 1.03]
High	5	90	16,331	0.86 [0.69, 1.06]	52.9	0.52, 1.41	0.98 [0.74, 1.01]
Unclear	2	20	2187	0.72 [0.42, 1.22]	59.5	–	0.74 [0.54, 0.93]
**Overall**	**10**	**187**	**27,671**	**0.87 [0.78, 0.96]**	**26.6**	**0.70, 1.07**	**0.93 [0.81, 1.00]**

Footnote: *MA* meta-analysis, *RR* relative risk, *CI* confidence interval, *IQR* interquartile range

Exercise also showed an improvement in QOL, standardised mean difference (SMD) 0.18 [95% CI 0.08, 0.28]; *I*^2^ = 74.3%; PI -0.18, 0.53], MES = 0.20 [IQR 0.07, 0.39]; 15 CSRs, 408 RCTs, 32,984 participants) when compared with various controls (Table 2). These improvements were greater observed for health related QOL when compared to overall QOL SMD 0.30 [95% CI 0.21, 0.39] vs 0.06 [− 0.08, 0.20] respectively. Again, the effects of exercise were duration and frequency dependent. For instance, sessions of more than 90 mins exerted a greater improvement in QOL as compared with sessions up to 90 min SMD 0.24 [95% CI 0.11, 0.37] versus 0.22 [− 0.30, 0.74]. Subgroup analyses by the type of condition showed that the magnitude of effect was the largest among patients with mental health conditions, followed by CVD and cancer. Physical activity exerted negative effects on QOL in patients with respiratory conditions (2 CSRs, 20 RCTs with 601 patients; SMD -0.97 [95% CI -1.43, 0.57]; *I*^2^ = 87.8%; MES = -0.46 [IQR-0.97, 0.05]). Subgroup analyses by risk of bias (ROB) in RCTs showed that RCTs at low or unclear ROB exerted greater improvements in QOL when compared to RCTs at a high ROB SMD 0.21 [95% CI 0.10, 0.31] versus 0.17 [0.03, 0.31]. Analogically, CSRs with moderate to high quality of evidence showed slightly greater improvements in QOL when compared with CSRs that relied on very low to low quality evidence SMD 0.19 [95% CI 0.05, 0.33] versus 0.15 [− 0.02, 0.32]. Please also see supplementary Table 9 more studies reporting QOL outcomes as mean difference (not quantitatively synthesised herein).

**Table 2:** Quantitative evidence synthesis for quality of life outcomes

Subgroup categories	No of SRs in MA	No of original studies in MA	No of participants in MA	Random-effects pooled SMD [95% CI]	I-squared [%]	Prediction interval	Median effect size [IQR]
**Type of QOL**							
Overall QOL	8	223	18,391	0.06 [−0.08, 0.20]	77.4	−0.36, 0.48	0.13 [−0.07, 0.26]
Health-related QOL	7	185	14,593	0.30 [0.21, 0.39]	2.8	0.18, 0.42	0.33 [0.17, 0.45]
**Disease type**							
Cancers	5	131	11,842	0.31 [0.21, 0.40]	0.0	0.19, 0.43	0.33 [0.17, 0.46]
CVDs	2	11	858	0.37 [−0.09, 0.84]	28.7	–	0.57 [0.26, 0.88]
Arthritis	3	77	5276	−0.00 [− 0.36, 0.36]	88.2	−4.44, 4.43	− 0.07 [− 0.25, 0.28]
Mental illness	1	39	2326	0.45 [0.07, 0.83]	–	–	–
Respiratory conditions	2	20	601	−0.97 [−1.43, 0.57]	87.8	–	−0.46 [− 0.97, 0.05]
Others	2	130	12,081	0.07 [0.04, 1.10]	0.0	−0.12, 0.26	0.13 [0.07, 0.15]
**Duration of intervention**							
Up to 1 year	8	103	7912	0.14 [−0.07, 0.34]	75.2	−0.53, 0.80	0.20 [0.05, 0.46]
More than 1 year	3	169	14,407	0.11 [0.01, 0.22]	27.9	−0.24, 0.47	0.14 [0.10, 0.30]
Not reported	4	136	10,665	0.25 [0.10, 0.40]	56.1	−0.33, 0.83	0.27 [0.10, 0.34]
**Session length**							
Up to 90 mins	2	53	4884	0.22 [−0.30, 0.74]	88.2	−6.22, 6.66	0.46 [−0.25, 0.48]
More than 90 mins	2	25	2209	0.24 [0.11, 0.37]	0.0	−0.58, 1.06	0.22 [0.10, 0.33]
Not reported	11	330	25,891	0.16 [0.04, 0.28]	74.2	−0.21, 0.53	0.16 [0.06, 0.34]
**Frequency per week**							
Up to 5 times	5	59	3997	−0.04 [−0.34, 0.26]	78.4	−1.00, 0.92	0.08 [−0.25, 0.22]
More than 5 times	4	104	7535	0.27 [0.07, 0.46]	74.5	−0.39, 0.93	0.33 [0.17, 0.46]
Not reported	6	245	21,452	0.24 [0.13, 0.35]	38.8	−0.03, 0.51	0.26 [0.13, 0.39]
**Evidence quality**							
Very low to low	8	124	12,431	0.15 [−0.02, 0.32]	63.8	−0.36, 0.66	0.16 [0.10, 0.33]
Moderate to high	7	284	20,553	0.19 [0.05, 0.33]	81.6	−0.24, 0.63	0.25 [0.06, 0.42]
**Risk of bias (RoB)**							
Low/unclear	4	97	7070	0.21 [0.10, 0.31]	23.6	−0.05, 0.46	0.22 [0.10, 0.26]
High	11	311	25,914	0.17 [0.03, 0.31]	77.1	−0.29, 0.63	0.17 [0.07, 0.45]
**Overall**	**15**	**408**	**32,984**	**0.18 [0.08, 0.28]**	**74.3**	**−0.18, 0.53**	**0.20 [0.07, 0.39]**

Footnote: *MA* meta-analysis, *SMD* standardised mean difference, *CI* confidence interval, *QOL* quality of life, *IQR* interquartile range

Adverse events (AEs) were reported in 100 (66.6%) CSRs; and not reported in 50 (33.3%). The number of AEs ranged from 0 to 84 in the CSRs. The number was inestimable in 83 (55.3%) CSRs. Ten (6.6%) reported no occurrence of AEs. Mild AEs were reported in 28 (18.6%) CSRs, moderate in 9 (6%) and serious/severe in 20 (13.3%). There were 10 deaths and in majority of instances, the causality was not attributed to exercise. For this outcome, we were unable to pool the data as effect sizes were too heterogeneous (Table 3).

**Table 3:** Quantitative evidence synthesis of AEs and adherence outcomes

Outcome type	No of SRs in MA	No of original studies in MA	No of participants in MA	Summary effect size	Random-effects pooled estimate [95% CI]	I-squared [%]	Prediction interval	Median effect size [IQR]
**A & E**	2	9	542	MD	0.20 [−0.23, 0.63]	69.0	–	0.27 [0.04, 0.27]
	3	73	4494	RD	0.03 [0.01, 0.05]	0.0	−0.09, 0.16	0.03 [0.02, 0.03]
	11	314	94,730	RR	1.09 [0.84, 1.34]	5.7	0.71, 1.47	1.25 [1.07, 3.77]
**Adherence**	3	65	3508	RD	−0.001 [−0.01, 0.01]	0.0	−0.06, 0.06	−0.01 [− 0.05, 0.00]
	15	235	16,399	RR	0.81 [0.66, 0.96]	92.8	0.32, 1.30	1.13 [0.93, 1.63]

Footnote: *MA* meta-analysis, *RR* relative risk, *MD* mean difference, *RD* risk difference, *CI* confidence interval, *IQR* interquartile range

In 38 CSRs, the total number of trials reporting withdrawals/non-adherence was inestimable. There were different ways of reporting it such as adherence or attrition (high in 23.3% of CSRs) as well as various effect estimates including %, range, total numbers, MD, RD, RR, OR, mean and SD. The overall pooled estimates are reported in Table 3.

Of all 16 domains of the AMSTAR-2 tool, 1876 (78.1%) scored ‘yes’, 76 (3.1%) ‘partial yes’; 375 (15.6%) ‘no’, and ‘not applicable’ in 25 (1%) CSRs. Ninety-six CSRs (64%) were scored as ‘no’ on reporting sources of funding for the studies followed by 88 (58.6%) failing to explain the selection of study designs for inclusion. One CSR (0.6%) each were judged as ‘no’ for reporting any potential sources of conflict of interest, including any funding for conducting the review as well for performing study selection in duplicate.

In 102 (68%) CSRs, there was predominantly a high risk of bias in RCTs. In 9 (6%) studies, this was reported as a range, e.g., low or unclear or low to high. Two CSRs used different terminology i.e., moderate methodological quality; and the risk of bias was inestimable in one CSR. Sixteen (10.6%) CSRs did not identify any studies (RCTs) at low risk of random sequence generation, 28 (18.6%) allocation concealment, 28 (18.6%) performance bias, 84 (54%) detection bias, 35 (23.3%) attrition bias, 18 (12%) reporting bias, and 29 (19.3%) other bias.

In 114 (76%) CSRs, limitation of studies was the main reason for downgrading the quality of the evidence followed by imprecision in 98 (65.3%) and inconsistency in 68 (45.3%). Publication bias was the least frequent reason for downgrading in 26 (17.3%) CSRs. Ninety-one (60.7%) CSRs reached equivocal conclusions, 49 (32.7%) reviews reached positive conclusions and 10 (6.7%) reached negative conclusions (as judged by the authors of CSRs).

## Discussion

In this systematic review of CSRs, we found a large body of evidence on the beneficial effects of physical activity/exercise on health outcomes in a wide range of heterogeneous populations. Our data shows a 13% reduction in mortality rates among 27,671 participants, and a small improvement in QOL and health-related QOL following various modes of physical activity/exercises. This means that both healthy individuals and medically compromised patients can significantly improve function, physical and mental health; or reduce pain and disability by exercising more [190]. In line with previous findings [191–194], where a dose-specific reduction in mortality has been found, our data shows a greater reduction in mortality in studies with longer follow-up (> 12 months) as compared to those with shorter follow-up (< 12 months). Interestingly, we found a consistent pattern in the findings, the higher the quality of evidence and the lower the risk of bias in primary studies, the smaller reductions in mortality. This pattern is observational in nature and cannot be over-generalised; however this might mean less certainty in the estimates measured. Furthermore, we found that the magnitude of the effect size was the largest among patients with mental health conditions. A possible mechanism of action may involve elevated levels of brain-derived neurotrophic factor or beta-endorphins [195].

We found the issue of poor reporting or underreporting of adherence/withdrawals in over a quarter of CSRs (25.3%). This is crucial both for improving the accuracy of the estimates at the RCT level as well as maintaining high levels of physical activity and associated health benefits at the population level.

Even the most promising interventions are not entirely risk-free; and some minor AEs such as post-exercise pain and soreness or discomfort related to physical activity/exercise have been reported. These were typically transient; resolved within a few days; and comparable between exercise and various control groups. However worryingly, the issue of poor reporting or underreporting of AEs has been observed in one third of the CSRs. Transparent reporting of AEs is crucial for identifying patients at risk and mitigating any potential negative or unintended consequences of the interventions.

High risk of bias of the RCTs evaluated was evident in more than two thirds of the CSRs. For example, more than half of reviews identified high risk of detection bias as a major source of bias suggesting that lack of blinding is still an issue in trials of behavioural interventions. Other shortcomings included insufficiently described randomisation and allocation concealment methods and often poor outcome reporting. This highlights the methodological challenges in RCTs of exercise and the need to counterbalance those with the underlying aim of strengthening internal and external validity of these trials.

Overall, high risk of bias in the primary trials was the main reason for downgrading the quality of the evidence using the GRADE criteria. Imprecision was frequently an issue, meaning the effective sample size was often small; studies were underpowered to detect the between-group differences. Pooling too heterogeneous results often resulted in inconsistent findings and inability to draw any meaningful conclusions. Indirectness and publication bias were lesser common reasons for downgrading. However, with regards to the latter, the generally accepted minimum number of 10 studies needed for quantitatively estimate the funnel plot asymmetry was not present in 69 (46%) CSRs.

Strengths of this research are the inclusion of large number of ‘gold standard’ systematic reviews, robust screening, data extractions and critical methodological appraisal. Nevertheless, some weaknesses need to be highlighted when interpreting findings of this overview. For instance, some of these CSRs analysed the same primary studies (RCTs) but, arrived at slightly different conclusions. Using, the Pieper et al. [39] formula, the amount of overlap ranged from 0.01% for AEs to 0.2% for adherence, which indicates slight overlap. All CSRs are vulnerable to publication bias [196] - hence the conclusions generated by them may be false-positive. Also, exercise was sometimes part of a complex intervention; and the effects of physical activity could not be distinguished from co-interventions. Often there were confounding effects of diet, educational, behavioural or lifestyle interventions; selection, and measurement bias were inevitably inherited in this overview too. Also, including CSRs only might lead to selection bias; and excluding reviews published before 2000 might limit the overall completeness and applicability of the evidence. A future update should consider these limitations, and in particular also including non-CSRs.

## Conclusions

Trialists must improve the quality of primary studies. At the same time, strict compliance with the reporting standards should be enforced. Authors of CSRs should better explain eligibility criteria and report sources of funding for the primary studies. There are still insufficient physical activity trends worldwide amongst all age groups; and scalable interventions aimed at increasing physical activity levels should be prioritized [197]. Hence, policymakers and practitioners need to design and implement comprehensive and coordinated strategies aimed at targeting physical activity programs/interventions, health promotion and disease prevention campaigns at local, regional, national, and international levels [198].

## Supplementary Information

Additional file 1: **Supplementary Table 1.** Main characteristics of included Cochrane systematic reviews evaluating the effects of physical activity/exercise on health outcomes (*n* = 150). **Supplementary Table 2.** Additional information from Cochrane systematic reviews of the effects of physical activity/exercise on health outcomes (*n* = 150). **Supplementary Table 3.** Conclusions from Cochrane systematic reviews “quote”. **Supplementary Table 4**. AEs reported in Cochrane systematic reviews. **Supplementary Table 5.** Summary of withdrawals/non-adherence. **Supplementary Table 6.** Methodological quality assessment of the included Cochrane reviews with AMSTAR-2. **Supplementary Table 7.** Number of studies assessed as low risk of bias per domain. **Supplementary Table 8.** GRADE for the review’s main comparison. **Supplementary Table 9.** Studies reporting quality of life outcomes as mean difference.

## Data Availability

Data sharing is not applicable to this article as no raw data were analysed during the current study. All information in this article is based on published systematic reviews.

## Abbreviations

AEs: Adverse events
CVD: Cardiovascular diseases
CDSR: Cochrane Database of Systematic Reviews
CSRs: Cochrane systematic reviews
CI: Confidence interval
GRADE: Grading of Recommendations Assessment, Development and Evaluation
HR: Hazard ratio
IQR: Interquartile range
MD: Mean difference
OR: Odds ratio
PI: Prediction interval
QOL: Quality of life
RCTs: Randomised controlled trials
RR: Relative risk
RD: Risk difference
ROB: Risk of bias
SE: Standard error
SMD: Standardised mean difference
WHO: World Health Organization

## References

[1] https://www.who.int/dietphysicalactivity/pa/en/. (Accessed 8 June 2020).

[2] Piercy KL, Troiano RP, Ballard RM, Carlson SA, Fulton JE, Galuska DA, George SM, Olson RD (2018). The physical activity guidelines for AmericansPhysical activity guidelines for AmericansPhysical activity guidelines for Americans. Jama.

[3] Kyu HH, Bachman VF, Alexander LT, Mumford JE, Afshin A, Estep K, Veerman JL, Delwiche K, Iannarone ML, Moyer ML (2016). Physical activity and risk of breast cancer, colon cancer, diabetes, ischemic heart disease, and ischemic stroke events: systematic review and dose-response meta-analysis for the global burden of disease study 2013. BMJ.

[4] Abell B, Glasziou P, Hoffmann T (2017). The contribution of individual exercise training components to clinical outcomes in randomised controlled trials of cardiac rehabilitation: a systematic review and meta-regression. Sports Med Open.

[5] Anderson D, Seib C, Rasmussen L (2014). Can physical activity prevent physical and cognitive decline in postmenopausal women? A systematic review of the literature. Maturitas.

[6] Barbaric M, Brooks E, Moore L, Cheifetz O (2010). Effects of physical activity on cancer survival: a systematic review. Physiother Can.

[7] Barlow PA, Otahal P, Schultz MG, Shing CM, Sharman JE (2014). Low exercise blood pressure and risk of cardiovascular events and all-cause mortality: systematic review and meta-analysis. Atherosclerosis.

[8] Aljawarneh YM, Wardell DW, Wood GL, Rozmus CL. A systematic review of physical activity and exercise on physiological and biochemical outcomes in children and adolescents with type 1 diabetes. J Nurs Scholarsh. 2019. 10.1111/jnu.12472 30895735

[9] Chastin SFM, De Craemer M, De Cocker K, Powell L, Van Cauwenberg J, Dall P, Hamer M, Stamatakis E (2019). How does light-intensity physical activity associate with adult cardiometabolic health and mortality? Systematic review with meta-analysis of experimental and observational studies. Br J Sports Med.

[10] Abdulla SY, Southerst D, Cote P, Shearer HM, Sutton D, Randhawa K, Varatharajan S, Wong JJ, Yu H, Marchand AA (2015). Is exercise effective for the management of subacromial impingement syndrome and other soft tissue injuries of the shoulder? A systematic review by the Ontario protocol for traffic injury management (OPTIMa) collaboration. Man Ther.

[11] Alanazi MH, Parent EC, Dennett E (2018). Effect of stabilization exercise on back pain, disability and quality of life in adults with scoliosis: a systematic review. Eur J Phys Rehabil Med.

[12] Adsett JA, Mudge AM, Morris N, Kuys S, Paratz JD (2015). Aquatic exercise training and stable heart failure: a systematic review and meta-analysis. Int J Cardiol.

[13] Adamson BC, Ensari I, Motl RW (2015). Effect of exercise on depressive symptoms in adults with neurologic disorders: a systematic review and meta-analysis. Arch Phys Med Rehabil.

[14] Abdin S, Welch RK, Byron-Daniel J, Meyrick J (2018). The effectiveness of physical activity interventions in improving well-being across office-based workplace settings: a systematic review. Public Health.

[15] Albalawi H, Coulter E, Ghouri N, Paul L (2017). The effectiveness of structured exercise in the south Asian population with type 2 diabetes: a systematic review. Phys Sportsmed.

[16] Sellami M, Gasmi M, Denham J, Hayes LD, Stratton D, Padulo J, Bragazzi N (2018). Effects of acute and chronic exercise on immunological parameters in the elderly aged: can physical activity counteract the effects of aging?. Front Immunol.

[17] Hagen KB, Dagfinrud H, Moe RH, Østerås N, Kjeken I, Grotle M, Smedslund G (2012). Exercise therapy for bone and muscle health: an overview of systematic reviews. BMC Med.

[18] Burton DA, Stokes K, Hall GM (2004). Physiological effects of exercise. Contin Educ Anaesth Crit Care Pain.

[19] Kraemer WJ, Ratamess NA (2005). Hormonal responses and adaptations to resistance exercise and training. Sports Med.

[20] Nystoriak MA, Bhatnagar A (2018). Cardiovascular effects and benefits of exercise. Front Cardiovasc Med.

[21] Vina J, Sanchis-Gomar F, Martinez-Bello V, Gomez-Cabrera MC (2012). Exercise acts as a drug; the pharmacological benefits of exercise. Br J Pharmacol.

[22] Warburton DER, Bredin SSD (2017). Health benefits of physical activity: a systematic review of current systematic reviews. Curr Opin Cardiol.

[23] Excellence NIfHaC: Cardiovascular disease prevention public health guideline [PH25] Published date: June 2010. Available at: https://www.nice.org.uk/guidance/ph25/resources/cardiovascular-disease-prevention-pdf-1996238687173.

[24] Excellence NIfHaC: Obesity prevention clinical guideline [CG43] published date: December 2006 Last updated: March 2015. Available at: https://www.nice.org.uk/guidance/cg43/resources/obesity-prevention-pdf-975445344709.

[25] Excellence NIfHaC: Physical activity for children and young people public health guideline [PH17] Published date: January 2009. Available at: https://www.nice.org.uk/guidance/ph17/resources/physical-activity-for-children-and-young-people-pdf-1996181580229.

[26] Higgins J, Green S. Cochrane Handbook for Systematic Reviews of Interventions. Version 6 [updated September 2018] edition. Available from www.cochrane-handbook.org: The Cochrane Collaboration, 2011. 2011.

[27] Bougioukas KI, Liakos A, Tsapas A, Ntzani E, Haidich A-B (2018). Preferred reporting items for overviews of systematic reviews including harms checklist: a pilot tool to be used for balanced reporting of benefits and harms. J Clin Epidemiol.

[28] Pollock M, Fernandes RM, Newton AS, Scott SD, Hartling L (2019). The impact of different inclusion decisions on the comprehensiveness and complexity of overviews of reviews of healthcare interventions. Syst Rev.

[29] Handoll H, Madhok R (2002). Quality of Cochrane reviews. Another study found that most Cochrane reviews are of a good standard. BMJ.

[30] Petticrew M, Wilson P, Wright K, Song F (2002). Quality of Cochrane reviews. Quality of Cochrane reviews is better than that of non-Cochrane reviews. BMJ.

[31] Shea B, Moher D, Graham I, Pham B, Tugwell P (2002). A comparison of the quality of Cochrane reviews and systematic reviews published in paper-based journals. Eval Health Prof.

[32] Shea BJ, Reeves BC, Wells G, Thuku M, Hamel C, Moran J, Moher D, Tugwell P, Welch V, Kristjansson E (2017). AMSTAR 2: a critical appraisal tool for systematic reviews that include randomised or non-randomised studies of healthcare interventions, or both. BMJ.

[33] Posadzki PP, Bajpai R, Kyaw BM, Roberts NJ, Brzezinski A, Christopoulos GI, Divakar U, Bajpai S, Soljak M, Dunleavy G (2018). Melatonin and health: an umbrella review of health outcomes and biological mechanisms of action. BMC Med.

[34] Bellou V, Belbasis L, Tzoulaki I, Evangelou E, Ioannidis JP (2016). Environmental risk factors and Parkinson's disease: an umbrella review of meta-analyses. Parkinsonism Relat Disord.

[35] Walter SD, Cook RJ (1991). A comparison of several point estimators of the odds ratio in a single 2 x 2 contingency table. Biometrics.

[36] Khan H, Sempos CT (1989). Statistical methods in epidemiology.

[37] DerSimonian R, Laird N (1986). Meta-analysis in clinical trials. Control Clin Trials.

[38] Higgins J, Thomas J, Chandler J, Cumpston M, Li T, Page MJ, Welch VA, editors. Cochrane Handbook for Systematic Reviews of Interventions version 6.0 (updated July 2019): Cochrane; 2019. Available from www.training.cochrane.org/handbook. 10.1002/14651858.ED000142 PMC10284251 31643080

[39] Pieper D, Antoine SL, Mathes T, Neugebauer EA, Eikermann M (2014). Systematic review finds overlapping reviews were not mentioned in every other overview. J Clin Epidemiol.

[40] Adeniyi FB, Young T. Weight loss interventions for chronic asthma. Cochrane Database Syst Rev. 2012;7. 10.1002/14651858.CD009339.pub2 PMC12075998 22786526

[41] Al-Khudairy L, Loveman E, Colquitt JL, Mead E, Johnson RE, Fraser H, Olajide J, Murphy M, Velho RM, O'Malley C, et al. Diet, physical activity and behavioural interventions for the treatment of overweight or obese adolescents aged 12 to 17 years. Cochrane Database Syst Rev. 2017;6. 10.1002/14651858.CD012691 PMC6481371 28639320

[42] Amorim Adegboye AR, Linne YM. Diet or exercise, or both, for weight reduction in women after childbirth. Cochrane Database Syst Rev. 2013;7. 10.1002/14651858.CD005627.pub3 PMC9392837 23881656

[43] Anderson L, Nguyen TT, Dall CH, Burgess L, Bridges C, Taylor RS. Exercise-based cardiac rehabilitation in heart transplant recipients. Cochrane Database Syst Rev. 2017;4. 10.1002/14651858.CD012264.pub2 PMC6478176 28375548

[44] Anderson L, Thompson DR, Oldridge N, Zwisler AD, Rees K, Martin N, Taylor RS. Exercise-based cardiac rehabilitation for coronary heart disease. Cochrane Database Syst Rev. 2016;1. 10.1002/14651858.CD001800.pub3 PMC6491180 26730878

[45] Andriolo RB, El Dib RP, Ramos L, Atallah Á, da Silva EMK. Aerobic exercise training programmes for improving physical and psychosocial health in adults with Down syndrome. Cochrane Database Syst Rev. 2010;5. 10.1002/14651858.CD005176.pub4 PMC12184876 20464738

[46] Araujo DN, Ribeiro CTD, Maciel ACC, Bruno SS, Fregonezi GAF, Dias FAL. Physical exercise for the treatment of non-ulcerated chronic venous insufficiency. Cochrane Database Syst Rev. 2016;12. 10.1002/14651858.CD010637.pub2 PMC6463841 27914110

[47] Ashworth NL, Chad KE, Harrison EL, Reeder BA, Marshall SC. Home versus center based physical activity programs in older adults. Cochrane Database Syst Rev. 2005;1. 10.1002/14651858.CD004017.pub2 PMC6464851 15674925

[48] Bartels EM, Juhl CB, Christensen R, Hagen KB, Danneskiold-Samsøe B, Dagfinrud H, Lund H. Aquatic exercise for the treatment of knee and hip osteoarthritis. Cochrane Database Syst Rev. 2016;3. 10.1002/14651858.CD005523.pub3 PMC9942938 27007113

[49] Beggs S, Foong YC, Le HCT, Noor D, Wood-Baker R, Walters JAE. Swimming training for asthma in children and adolescents aged 18 years and under. Cochrane Database Syst Rev. 2013;4. 10.1002/14651858.CD009607.pub2 PMC12193991 23633375

[50] Bergenthal N, Will A, Streckmann F, Wolkewitz KD, Monsef I, Engert A, Elter T, Skoetz N. Aerobic physical exercise for adult patients with haematological malignancies. Cochrane Database Syst Rev. 2014;11. 10.1002/14651858.CD009075.pub2 25386666

[51] Bidonde J, Busch AJ, Schachter CL, Overend TJ, Kim SY, Góes SM, Boden C, Foulds HJA. Aerobic exercise training for adults with fibromyalgia. Cochrane Database Syst Rev. 2017;6. 10.1002/14651858.CD012700 PMC6481524 28636204

[52] Bidonde J, Busch AJ, van der Spuy I, Tupper S, Kim SY, Boden C. Whole body vibration exercise training for fibromyalgia. Cochrane Database Syst Rev. 2017;9. 10.1002/14651858.CD011755.pub2 PMC6483692 28950401

[53] Bidonde J, Busch AJ, Webber SC, Schachter CL, Danyliw A, Overend TJ, Richards RS, Rader T. Aquatic exercise training for fibromyalgia. Cochrane Database Syst Rev. 2014;10. 10.1002/14651858.CD011336 PMC10638613 25350761

[54] Braam KI, van der Torre P, Takken T, Veening MA, van Dulmen-den Broeder E, Kaspers GJL. Physical exercise training interventions for children and young adults during and after treatment for childhood cancer. Cochrane Database Syst Rev. 2016;3. 10.1002/14651858.CD008796.pub3 PMC6464400 27030386

[55] Bradt J, Shim M, Goodill SW. Dance/movement therapy for improving psychological and physical outcomes in cancer patients. Cochrane Database Syst Rev. 2015;1. 10.1002/14651858.CD007103.pub3 PMC7204197 25565627

[56] Broderick J, Crumlish N, Waugh A, Vancampfort D. Yoga versus non-standard care for schizophrenia. Cochrane Database Syst Rev. 2017;9. 10.1002/14651858.CD012052.pub2 PMC6483630 28956893

[57] Broderick J, Knowles A, Chadwick J, Vancampfort D. Yoga versus standard care for schizophrenia. Cochrane Database Syst Rev. 2015;10. 10.1002/14651858.CD010554.pub2 PMC9446089 26488850

[58] Broderick J, Vancampfort D. Yoga as part of a package of care versus standard care for schizophrenia. Cochrane Database Syst Rev. 2017;9. 10.1002/14651858.CD012145.pub2 PMC6483611 28960019

[59] Brown J, Ceysens G, Boulvain M. Exercise for pregnant women with gestational diabetes for improving maternal and fetal outcomes. Cochrane Database Syst Rev. 2017;6. 10.1002/14651858.CD012202.pub2 PMC6481507 28639706

[60] Busch AJ, Barber KA, Overend TJ, Peloso PMJ, Schachter CL. Exercise for treating fibromyalgia syndrome. Cochrane Database Syst Rev. 2007;4. 10.1002/14651858.CD003786.pub2 PMC12832165 17943797

[61] Busch AJ, Webber SC, Richards RS, Bidonde J, Schachter CL, Schafer LA, Danyliw A, Sawant A, Dal Bello-Haas V, Rader T, et al. Resistance exercise training for fibromyalgia. Cochrane Database Syst Rev. 2013;12. 10.1002/14651858.CD010884 PMC6544808 24362925

[62] Cameron ID, Dyer SM, Panagoda CE, Murray GR, Hill KD, Cumming RG, Kerse N. Interventions for preventing falls in older people in care facilities and hospitals. Cochrane Database Syst Rev. 2018;9. 10.1002/14651858.CD005465.pub4 PMC6148705 30191554

[63] Carson KV, Chandratilleke MG, Picot J, Brinn MP, Esterman AJ, Smith BJ. Physical training for asthma. Cochrane Database Syst Rev. 2013;9. 10.1002/14651858.CD001116.pub4 PMC11930393 24085631

[64] Carvalho APV, Vital FMR, Soares BGO. Exercise interventions for shoulder dysfunction in patients treated for head and neck cancer. Cochrane Database Syst Rev. 2012;4. 10.1002/14651858.CD008693.pub2 PMC11537249 22513964

[65] Cavalheri V, Granger C. Preoperative exercise training for patients with non-small cell lung cancer. Cochrane Database Syst Rev. 2017;6. 10.1002/14651858.CD012020.pub2 PMC6481477 28589547

[66] Cavalheri V, Tahirah F, Nonoyama ML, Jenkins S, Hill K. Exercise training undertaken by people within 12 months of lung resection for non-small cell lung cancer. Cochrane Database Syst Rev. 2013;7. 10.1002/14651858.CD009955.pub2 23904353

[67] Ceysens G, Rouiller D, Boulvain M. Exercise for diabetic pregnant women. Cochrane Database Syst Rev. 2006;3. 10.1002/14651858.CD004225.pub2 PMC8406944 16856038

[68] Choi BKL, Verbeek JH, Tam WWS, Jiang JY. Exercises for prevention of recurrences of low-back pain. Cochrane Database Syst Rev. 2010;1. 10.1002/14651858.CD006555.pub2 PMC8078403 20091596

[69] Colquitt JL, Loveman E, O'Malley C, Azevedo LB, Mead E, Al-Khudairy L, Ells LJ, Metzendorf MI, Rees K. Diet, physical activity, and behavioural interventions for the treatment of overweight or obesity in preschool children up to the age of 6 years. Cochrane Database Syst Rev. 2016;3. 10.1002/14651858.CD012105 PMC6669248 26961576

[70] Connolly B, Salisbury L, O'Neill B, Geneen LJ, Douiri A, Grocott MPW, Hart N, Walsh TS, Blackwood B. Exercise rehabilitation following intensive care unit discharge for recovery from critical illness. Cochrane Database Syst Rev. 2015;6. 10.1002/14651858.CD008632.pub2 PMC6517154 26098746

[71] Cooney GM, Dwan K, Greig CA, Lawlor DA, Rimer J, Waugh FR, McMurdo M, Mead GE. Exercise for depression. Cochrane Database Syst Rev. 2013;9. 10.1002/14651858.CD004366.pub6 PMC9721454 24026850

[72] Corbetta D, Sirtori V, Castellini G, Moja L, Gatti R. Constraint-induced movement therapy for upper extremities in people with stroke. Cochrane Database Syst Rev. 2015;10. 10.1002/14651858.CD004433.pub3 PMC6465192 26446577

[73] Cramer H, Lauche R, Klose P, Lange S, Langhorst J, Dobos GJ. Yoga for improving health-related quality of life, mental health and cancer-related symptoms in women diagnosed with breast cancer. Cochrane Database Syst Rev. 2017;1. 10.1002/14651858.CD010802.pub2 PMC6465041 28045199

[74] Cramp F, Byron-Daniel J. Exercise for the management of cancer-related fatigue in adults. Cochrane Database Syst Rev. 2012;11. 10.1002/14651858.CD006145.pub3 PMC8480137 23152233

[75] Dal Bello-Haas V, Florence JM. Therapeutic exercise for people with amyotrophic lateral sclerosis or motor neuron disease. Cochrane Database Syst Rev. 2013;5. 10.1002/14651858.CD005229.pub3 PMC6769061 23728653

[76] Dale MT, McKeough ZJ, Troosters T, Bye P, Alison JA. Exercise training to improve exercise capacity and quality of life in people with non-malignant dust-related respiratory diseases. Cochrane Database Syst Rev. 2015;11. 10.1002/14651858.CD009385.pub2 PMC9297006 26544672

[77] Daley A, Stokes-Lampard H, Thomas A, MacArthur C. Exercise for vasomotor menopausal symptoms. Cochrane Database Syst Rev. 2014;11. 10.1002/14651858.CD006108.pub4 PMC10116337 25431132

[78] de Morton N, Keating JL, Jeffs K. Exercise for acutely hospitalised older medical patients. Cochrane Database Syst Rev. 2007;1. 10.1002/14651858.CD005955.pub2 PMC8906511 17253572

[79] Dobbins M, Husson H, DeCorby K, LaRocca RL. School-based physical activity programs for promoting physical activity and fitness in children and adolescents aged 6 to 18. Cochrane Database Syst Rev. 2013;2. 10.1002/14651858.CD007651.pub2 PMC7197501 23450577

[80] Doiron KA, Hoffmann TC, Beller EM. Early intervention (mobilization or active exercise) for critically ill adults in the intensive care unit. Cochrane Database Syst Rev. 2018;3. 10.1002/14651858.CD010754.pub2 PMC6494211 29582429

[81] Ekeland E, Heian F, Hagen KB, Abbott JM, Nordheim L. Exercise to improve self-esteem in children and young people. Cochrane Database Syst Rev. 2004;1. 10.1002/14651858.CD003683.pub2 PMC12935395 14974029

[82] Elbers RG, Verhoef J, van Wegen EEH, Berendse HW, Kwakkel G. Interventions for fatigue in Parkinson's disease. Cochrane Database Syst Rev. 2015;10. 10.1002/14651858.CD010925.pub2 PMC9240814 26447539

[83] Felbel S, Meerpohl JJ, Monsef I, Engert A, Skoetz N. Yoga in addition to standard care for patients with haematological malignancies. Cochrane Database Syst Rev. 2014;6. 10.1002/14651858.CD010146.pub2 PMC4869525 24919720

[84] Forbes D, Forbes SC, Blake CM, Thiessen EJ, Forbes S. Exercise programs for people with dementia. Cochrane Database Syst Rev. 2015;4. 10.1002/14651858.CD006489.pub4 PMC9426996 25874613

[85] Fransen M, McConnell S, Harmer AR, Van der Esch M, Simic M, Bennell KL. Exercise for osteoarthritis of the knee. Cochrane Database Syst Rev. 2015;1. 10.1002/14651858.CD004376.pub3 PMC10094004 25569281

[86] Fransen M, McConnell S, Hernandez-Molina G, Reichenbach S. Exercise for osteoarthritis of the hip. Cochrane Database Syst Rev. 2014;4. 10.1002/14651858.CD007912.pub2 PMC10898220 24756895

[87] Freitas DA, Holloway EA, Bruno SS, Chaves GSS, Fregonezi GAF, Mendonça K. Breathing exercises for adults with asthma. Cochrane Database Syst Rev. 2013;10. 10.1002/14651858.CD001277.pub3 24085551

[88] Furmaniak AC, Menig M, Markes MH. Exercise for women receiving adjuvant therapy for breast cancer. Cochrane Database Syst Rev. 2016;9. 10.1002/14651858.CD005001.pub3 PMC6457768 27650122

[89] Giangregorio LM, MacIntyre NJ, Thabane L, Skidmore CJ, Papaioannou A. Exercise for improving outcomes after osteoporotic vertebral fracture. Cochrane Database Syst Rev. 2013;1. 10.1002/14651858.CD008618.pub2 PMC5104540 23440829

[90] Gillespie LD, Robertson MC, Gillespie WJ, Sherrington C, Gates S, Clemson LM, Lamb SE. Interventions for preventing falls in older people living in the community. Cochrane Database Syst Rev. 2012;9. 10.1002/14651858.CD007146.pub3 PMC8095069 22972103

[91] Gorczynski P, Faulkner G. Exercise therapy for schizophrenia. Cochrane Database Syst Rev. 2010;5. 10.1002/14651858.CD004412.pub2 PMC4164954 20464730

[92] Grande AJ, Keogh J, Hoffmann TC, Beller EM, Del Mar CB. Exercise versus no exercise for the occurrence, severity and duration of acute respiratory infections. Cochrane Database Syst Rev. 2015;6. 10.1002/14651858.CD010596.pub2 26077724

[93] Grande AJ, Reid H, Thomas EE, Nunan D, Foster C. Exercise prior to influenza vaccination for limiting influenza incidence and its related complications in adults. Cochrane Database Syst Rev. 2016;8. 10.1002/14651858.CD011857.pub2 PMC8504432 27545762

[94] Grande AJ, Silva V, Andriolo BNG, Riera R, Parra SA, Peccin MS. Water-based exercise for adults with asthma. Cochrane Database Syst Rev. 2014;7. 10.1002/14651858.CD010456.pub2 PMC11252722 25032820

[95] Gross A, Kay TM, Paquin JP, Blanchette S, Lalonde P, Christie T, Dupont G, Graham N, Burnie SJ, Gelley G, et al. Exercises for mechanical neck disorders. Cochrane Database Syst Rev. 2015;1. 10.1002/14651858.CD004250.pub5 PMC9508492 25629215

[96] Hageman D, Fokkenrood HJP, Gommans LNM, van den Houten MML, Teijink JAW. Supervised exercise therapy versus home-based exercise therapy versus walking advice for intermittent claudication. Cochrane Database Syst Rev. 2018;4. 10.1002/14651858.CD005263.pub4 PMC6513337 29627967

[97] Han A, Judd M, Welch V, Wu T, Tugwell P, Wells GA. Tai chi for treating rheumatoid arthritis. Cochrane Database Syst Rev. 2004;3. 10.1002/14651858.CD004849 15266544

[98] Han S, Middleton P, Crowther CA. Exercise for pregnant women for preventing gestational diabetes mellitus. Cochrane Database Syst Rev. 2012;7. 10.1002/14651858.CD009021.pub2 PMC11534277 22786521

[99] Hartley L, Dyakova M, Holmes J, Clarke A, Lee MS, Ernst E, Rees K. Yoga for the primary prevention of cardiovascular disease. Cochrane Database Syst Rev. 2014;5. 10.1002/14651858.CD010072.pub2 PMC10075056 24825181

[100] Hartley L, Flowers N, Lee MS, Ernst E, Rees K. Tai chi for primary prevention of cardiovascular disease. Cochrane Database Syst Rev. 2014;4. 10.1002/14651858.CD010366.pub2 PMC11185961 24715694

[101] Hartley L, Lee MS, Kwong JSW, Flowers N, Todkill D, Ernst E, Rees K. Qigong for the primary prevention of cardiovascular disease. Cochrane Database Syst Rev. 2015;6. 10.1002/14651858.CD010390.pub2 PMC6956616 26068956

[102] Hassett L, Moseley AM, Harmer AR. Fitness training for cardiorespiratory conditioning after traumatic brain injury. Cochrane Database Syst Rev. 2017;12. 10.1002/14651858.CD006123.pub3 PMC6486048 29286534

[103] Hayden J, van Tulder MW, Malmivaara A, Koes BW. Exercise therapy for treatment of non-specific low back pain. Cochrane Database Syst Rev. 2005;3. 10.1002/14651858.CD000335.pub2 PMC10068907 16034851

[104] Hay-Smith EJC, Herderschee R, Dumoulin C, Herbison GP. Comparisons of approaches to pelvic floor muscle training for urinary incontinence in women. Cochrane Database Syst Rev. 2011;12. 10.1002/14651858.CD009508 22161451

[105] Heine M, van de Port I, Rietberg MB, van Wegen EEH, Kwakkel G. Exercise therapy for fatigue in multiple sclerosis. Cochrane Database Syst Rev. 2015;9. 10.1002/14651858.CD009956.pub2 PMC9554249 26358158

[106] Heiwe S, Jacobson SH. Exercise training for adults with chronic kidney disease. Cochrane Database Syst Rev. 2011;10. 10.1002/14651858.CD003236.pub2 PMC10183198 21975737

[107] Hemmingsen B, Gimenez-Perez G, Mauricio D, Roqué i Figuls M, Metzendorf MI, Richter B. Diet, physical activity or both for prevention or delay of type 2 diabetes mellitus and its associated complications in people at increased risk of developing type 2 diabetes mellitus. Cochrane Database Syst Rev. 2017;12. 10.1002/14651858.CD003054.pub4 PMC6486271 29205264

[108] Herbert RD, de Noronha M, Kamper SJ. Stretching to prevent or reduce muscle soreness after exercise. Cochrane Database Syst Rev. 2011;7. 10.1002/14651858.CD004577.pub3 21735398

[109] Heymans MW, van Tulder MW, Esmail R, Bombardier C, Koes BW. Back schools for non-specific low-back pain. Cochrane Database Syst Rev. 2004;4. 10.1002/14651858.CD000261.pub2 PMC12365949 15494995

[110] Holland AE, Hill CJ, Jones AY, McDonald CF. Breathing exercises for chronic obstructive pulmonary disease. Cochrane Database Syst Rev. 2012;10. 10.1002/14651858.CD008250.pub2 PMC11371308 23076942

[111] Howe TE, Rochester L, Neil F, Skelton DA, Ballinger C. Exercise for improving balance in older people. Cochrane Database Syst Rev. 2011;11. 10.1002/14651858.CD004963.pub3 PMC11493176 22071817

[112] Howe TE, Shea B, Dawson LJ, Downie F, Murray A, Ross C, Harbour RT, Caldwell LM, Creed G. Exercise for preventing and treating osteoporosis in postmenopausal women. Cochrane Database Syst Rev. 2011;7. 10.1002/14651858.CD000333.pub2 PMC12744941 21735380

[113] Hurkmans E, van der Giesen FJ, Vliet Vlieland TPM, Schoones J, Van den Ende E. Dynamic exercise programs (aerobic capacity and/or muscle strength training) in patients with rheumatoid arthritis. Cochrane Database Syst Rev. 2009;4. 10.1002/14651858.CD006853.pub2 PMC6769170 19821388

[114] Hurley M, Dickson K, Hallett R, Grant R, Hauari H, Walsh N, Stansfield C, Oliver S. Exercise interventions and patient beliefs for people with hip, knee or hip and knee osteoarthritis: a mixed methods review. Cochrane Database Syst Rev. 2018;4. 10.1002/14651858.CD010842.pub2 PMC6494515 29664187

[115] Jones M, Harvey A, Marston L, O'Connell NE. Breathing exercises for dysfunctional breathing/hyperventilation syndrome in adults. Cochrane Database Syst Rev. 2013;5. 10.1002/14651858.CD009041.pub2 PMC11379427 23728685

[116] Katsura M, Kuriyama A, Takeshima T, Fukuhara S, Furukawa TA. Preoperative inspiratory muscle training for postoperative pulmonary complications in adults undergoing cardiac and major abdominal surgery. Cochrane Database Syst Rev. 2015;10. 10.1002/14651858.CD010356.pub2 PMC9251477 26436600

[117] Kendrick D, Kumar A, Carpenter H, Zijlstra GAR, Skelton DA, Cook JR, Stevens Z, Belcher CM, Haworth D, Gawler SJ, et al. Exercise for reducing fear of falling in older people living in the community. Cochrane Database Syst Rev. 2014;11. 10.1002/14651858.CD009848.pub2 PMC7388865 25432016

[118] Kramer MS, McDonald SW. Aerobic exercise for women during pregnancy. Cochrane Database Syst Rev. 2006;3. 10.1002/14651858.CD000180.pub2 PMC7043271 16855953

[119] Lahart IM, Metsios GS, Nevill AM, Carmichael AR. Physical activity for women with breast cancer after adjuvant therapy. Cochrane Database Syst Rev. 2018;1. 10.1002/14651858.CD011292.pub2 PMC6491330 29376559

[120] Lane R, Harwood A, Watson L, Leng GC. Exercise for intermittent claudication. Cochrane Database Syst Rev. 2017;12. 10.1002/14651858.CD000990.pub4 PMC6486315 29278423

[121] Larun L, Brurberg KG, Odgaard-Jensen J, Price JR. Exercise therapy for chronic fatigue syndrome. Cochrane Database Syst Rev. 2017;4. 10.1002/14651858.CD003200.pub7 PMC6419524 28444695

[122] Larun L, Nordheim LV, Ekeland E, Hagen KB, Heian F. Exercise in prevention and treatment of anxiety and depression among children and young people. Cochrane Database Syst Rev. 2006;3. 10.1002/14651858.CD004691.pub2 PMC12742371 16856055

[123] Lauret GJ, Fakhry F, Fokkenrood HJP, Hunink MGM, Teijink JAW, Spronk S. Modes of exercise training for intermittent claudication. Cochrane Database Syst Rev. 2014;7. 10.1002/14651858.CD009638.pub2 24993079

[124] Lawrence M, Celestino Junior FT, Matozinho HHS, Govan L, Booth J, Beecher J. Yoga for stroke rehabilitation. Cochrane Database Syst Rev. 2017;12. 10.1002/14651858.CD011483.pub2 PMC6486003 29220541

[125] Lin CWC, Donkers NAJ, Refshauge KM, Beckenkamp PR, Khera K, Moseley AM. Rehabilitation for ankle fractures in adults. Cochrane Database Syst Rev. 2012;11. 10.1002/14651858.CD005595.pub3 23152232

[126] Liu CJ, Latham NK. Progressive resistance strength training for improving physical function in older adults. Cochrane Database Syst Rev. 2009;3. 10.1002/14651858.CD002759.pub2 PMC4324332 19588334

[127] Long L, Anderson L, Dewhirst AM, He J, Bridges C, Gandhi M, Taylor RS. Exercise-based cardiac rehabilitation for adults with stable angina. Cochrane Database Syst Rev. 2018;2. 10.1002/14651858.CD012786.pub2 PMC6491173 29394453

[128] Loughney LA, West MA, Kemp GJ, Grocott MPW, Jack S. Exercise interventions for people undergoing multimodal cancer treatment that includes surgery. Cochrane Database Syst Rev. 2018;12. 10.1002/14651858.CD012280.pub2 PMC6517034 30536366

[129] Macedo LG, Saragiotto BT, Yamato TP, Costa LOP, Menezes Costa LC, Ostelo R, Maher CG. Motor control exercise for acute non-specific low back pain. Cochrane Database Syst Rev. 2016;2. 10.1002/14651858.CD012085 PMC8734597 26863390

[130] Macêdo TMF, Freitas DA, Chaves GSS, Holloway EA, Mendonça K. Breathing exercises for children with asthma. Cochrane Database Syst Rev. 2016;4. 10.1002/14651858.CD011017.pub2 PMC7104663 27070225

[131] Martin A, Booth JN, Laird Y, Sproule J, Reilly JJ, Saunders DH. Physical activity, diet and other behavioural interventions for improving cognition and school achievement in children and adolescents with obesity or overweight. Cochrane Database Syst Rev. 2018;3. 10.1002/14651858.CD009728.pub4 PMC5865125 29499084

[132] McKeough ZJ, Velloso M, Lima VP, Alison JA. Upper limb exercise training for COPD. Cochrane Database Syst Rev. 2016;11. 10.1002/14651858.CD011434.pub2 PMC6464968 27846347

[133] McNamara RJ, McKeough ZJ, McKenzie DK, Alison JA. Water-based exercise training for chronic obstructive pulmonary disease. Cochrane Database Syst Rev. 2013;12. 10.1002/14651858.CD008290.pub2 PMC12075999 24353107

[134] McNeely ML, Campbell K, Ospina M, Rowe BH, Dabbs K, Klassen TP, Mackey J, Courneya K. Exercise interventions for upper-limb dysfunction due to breast cancer treatment. Cochrane Database Syst Rev. 2010;6. 10.1002/14651858.CD005211.pub2 PMC12861582 20556760

[135] Mead E, Brown T, Rees K, Azevedo LB, Whittaker V, Jones D, Olajide J, Mainardi GM, Corpeleijn E, O'Malley C, et al. Diet, physical activity and behavioural interventions for the treatment of overweight or obese children from the age of 6 to 11 years. Cochrane Database Syst Rev. 2017;6. 10.1002/14651858.CD012651 PMC6481885 28639319

[136] Meekums B, Karkou V, Nelson EA. Dance movement therapy for depression. Cochrane Database Syst Rev. 2015;2. 10.1002/14651858.CD009895.pub2 PMC8928931 25695871

[137] Meher S, Duley L. Exercise or other physical activity for preventing pre-eclampsia and its complications. Cochrane Database Syst Rev. 2006;2. 10.1002/14651858.CD005942 PMC8900135 16625645

[138] Mehrholz J, Kugler J, Pohl M. Water-based exercises for improving activities of daily living after stroke. Cochrane Database Syst Rev. 2011;1. 10.1002/14651858.CD008186.pub2 PMC6464732 21249701

[139] Mehrholz J, Kugler J, Pohl M. Locomotor training for walking after spinal cord injury. Cochrane Database Syst Rev. 2012;11. 10.1002/14651858.CD006676.pub3 PMC11848707 23152239

[140] Mehrholz J, Thomas S, Elsner B. Treadmill training and body weight support for walking after stroke. Cochrane Database Syst Rev. 2017;8. 10.1002/14651858.CD002840.pub4 PMC6483714 28815562

[141] Mishra SI, Scherer RW, Geigle PM, Berlanstein DR, Topaloglu O, Gotay CC, Snyder C. Exercise interventions on health-related quality of life for cancer survivors. Cochrane Database Syst Rev. 2012;8. 10.1002/14651858.CD007566.pub2 PMC7387117 22895961

[142] Mishra SI, Scherer RW, Snyder C, Geigle PM, Berlanstein DR, Topaloglu O. Exercise interventions on health-related quality of life for people with cancer during active treatment. Cochrane Database Syst Rev. 2012;8. 10.1002/14651858.CD008465.pub2 PMC7389071 22895974

[143] Montgomery P, Dennis JA. Physical exercise for sleep problems in adults aged 60+. Cochrane Database Syst Rev. 2002;4. 10.1002/14651858.CD003404 PMC7017641 12519595

[144] Morris NR, Kermeen FD, Holland AE. Exercise-based rehabilitation programmes for pulmonary hypertension. Cochrane Database Syst Rev. 2017;1. 10.1002/14651858.CD011285.pub2 PMC6464618 28099988

[145] Muktabhant B, Lawrie TA, Lumbiganon P, Laopaiboon M. Diet or exercise, or both, for preventing excessive weight gain in pregnancy. Cochrane Database Syst Rev. 2015;6. 10.1002/14651858.CD007145.pub3 PMC9428894 26068707

[146] Ngai SPC, Jones AYM, Tam WWS. Tai chi for chronic obstructive pulmonary disease (COPD). Cochrane Database Syst Rev. 2016;6. 10.1002/14651858.CD009953.pub2 PMC8504989 27272131

[147] Norton C, Cody JD. Biofeedback and/or sphincter exercises for the treatment of faecal incontinence in adults. Cochrane Database Syst Rev. 2012;7. 10.1002/14651858.CD002111.pub3 PMC11365095 22786479

[148] O'Brien K, Nixon S, Glazier R, Tynan AM. Progressive resistive exercise interventions for adults living with HIV/AIDS. Cochrane Database Syst Rev. 2004;4.10.1002/14651858.CD004248.pub215495092

[149] O'Brien K, Nixon S, Tynan AM, Glazier R. Aerobic exercise interventions for adults living with HIV/AIDS. Cochrane Database Syst Rev. 2010;8. 10.1002/14651858.CD001796.pub3 PMC7061352 20687068

[150] Østerås N, Kjeken I, Smedslund G, Moe RH, Slatkowsky-Christensen B, Uhlig T, Hagen KB. Exercise for hand osteoarthritis. Cochrane Database Syst Rev. 2017;1. 10.1002/14651858.CD010388.pub2 PMC6464796 28141914

[151] Page MJ, Green S, Kramer S, Johnston RV, McBain B, Chau M, Buchbinder R. Manual therapy and exercise for adhesive capsulitis (frozen shoulder). Cochrane Database Syst Rev. 2014;8. 10.1002/14651858.CD011275 PMC10882424 25157702

[152] Page MJ, Green S, McBain B, Surace SJ, Deitch J, Lyttle N, Mrocki MA, Buchbinder R. Manual therapy and exercise for rotator cuff disease. Cochrane Database Syst Rev. 2016;6. 10.1002/14651858.CD012224 PMC8570640 27283590

[153] Page MJ, O'Connor D, Pitt V, Massy-Westropp N. Exercise and mobilisation interventions for carpal tunnel syndrome. Cochrane Database Syst Rev. 2012;6. 10.1002/14651858.CD009899 PMC11536321 22696387

[154] Panebianco M, Sridharan K, Ramaratnam S. Yoga for epilepsy. Cochrane Database Syst Rev. 2017;10. 10.1002/14651858.CD001524.pub3 PMC6485327 28982217

[155] Perry A, Lee SH, Cotton S, Kennedy C. Therapeutic exercises for affecting post-treatment swallowing in people treated for advanced-stage head and neck cancers. Cochrane Database Syst Rev. 2016;8. 10.1002/14651858.CD011112.pub2 PMC7104309 27562477

[156] Radtke T, Nevitt SJ, Hebestreit H, Kriemler S. Physical exercise training for cystic fibrosis. Cochrane Database Syst Rev. 2017;11. 10.1002/14651858.CD002768.pub4 PMC6485991 29090734

[157] Regnaux JP, Lefevre-Colau MM, Trinquart L, Nguyen C, Boutron I, Brosseau L, Ravaud P. High-intensity versus low-intensity physical activity or exercise in people with hip or knee osteoarthritis. Cochrane Database Syst Rev. 2015;10. 10.1002/14651858.CD010203.pub2 PMC9270723 26513223

[158] Ren J, Xia J. Dance therapy for schizophrenia. Cochrane Database Syst Rev. 2013;10. 10.1002/14651858.CD006868.pub3 PMC11440587 24092546

[159] Rietberg MB, Brooks D, Uitdehaag BMJ, Kwakkel G. Exercise therapy for multiple sclerosis. Cochrane Database Syst Rev. 2005;1. 10.1002/14651858.CD003980.pub2 PMC6485797 15674920

[160] Risom SS, Zwisler AD, Johansen PP, Sibilitz KL, Lindschou J, Gluud C, Taylor RS, Svendsen JH, Berg SK. Exercise-based cardiac rehabilitation for adults with atrial fibrillation. Cochrane Database Syst Rev. 2017;2. 10.1002/14651858.CD011197.pub2 PMC6464537 28181684

[161] Romano M, Minozzi S, Bettany-Saltikov J, Zaina F, Chockalingam N, Kotwicki T, Maier-Hennes A, Negrini S. Exercises for adolescent idiopathic scoliosis. Cochrane Database Syst Rev. 2012;8. 10.1002/14651858.CD007837.pub2 PMC7386883 22895967

[162] Ryan JM, Cassidy EE, Noorduyn SG, O'Connell NE. Exercise interventions for cerebral palsy. Cochrane Database Syst Rev. 2017;6. 10.1002/14651858.CD011660.pub2 PMC6481791 28602046

[163] Saragiotto BT, Maher CG, Yamato TP, Costa LOP, Menezes Costa LC, Ostelo R, Macedo LG. Motor control exercise for chronic non-specific low-back pain. Cochrane Database Syst Rev. 2016;1. 10.1002/14651858.CD012004 PMC8761501 26742533

[164] Saunders DH, Sanderson M, Hayes S, Kilrane M, Greig CA, Brazzelli M, Mead GE. Physical fitness training for stroke patients. Cochrane Database Syst Rev. 2016;3. 10.1002/14651858.CD003316.pub6 PMC6464717 27010219

[165] Schulzke SM, Kaempfen S, Trachsel D, Patole SK. Physical activity programs for promoting bone mineralization and growth in preterm infants. Cochrane Database Syst Rev. 2014;4. 10.1002/14651858.CD005387.pub3 PMC11008108 24752440

[166] Seron P, Lanas F, Pardo Hernandez H, Bonfill Cosp X. Exercise for people with high cardiovascular risk. Cochrane Database Syst Rev. 2014;8. 10.1002/14651858.CD009387.pub2 PMC6669260 25120097

[167] Shaw KA, Gennat HC, O'Rourke P, Del Mar C. Exercise for overweight or obesity. Cochrane Database Syst Rev. 2006;4. 10.1002/14651858.CD003817.pub3 PMC9017288 17054187

[168] Shepherd E, Gomersall JC, Tieu J, Han S, Crowther CA, Middleton P. Combined diet and exercise interventions for preventing gestational diabetes mellitus. Cochrane Database Syst Rev. 2017;11. 10.1002/14651858.CD010443.pub3 PMC6485974 29129039

[169] Sibilitz KL, Berg SK, Tang LH, Risom SS, Gluud C, Lindschou J, Kober L, Hassager C, Taylor RS, Zwisler AD. Exercise-based cardiac rehabilitation for adults after heart valve surgery. Cochrane Database Syst Rev. 2016;3. 10.1002/14651858.CD010876.pub2 26998683

[170] Silva IS, Fregonezi GAF, Dias FAL, Ribeiro CTD, Guerra RO, Ferreira GMH. Inspiratory muscle training for asthma. Cochrane Database Syst Rev. 2013;9. 10.1002/14651858.CD003792.pub2 PMC7163283 24014205

[171] States RA, Pappas E, Salem Y. Overground physical therapy gait training for chronic stroke patients with mobility deficits. Cochrane Database Syst Rev. 2009;3. 10.1002/14651858.CD006075.pub2 PMC6464905 19588381

[172] Strike K, Mulder K, Michael R. Exercise for haemophilia. Cochrane Database Syst Rev. 2016;12. 10.1002/14651858.CD011180.pub2 PMC6463808 27992070

[173] Takken T, Van Brussel M, Engelbert RH, van der Net JJ, Kuis W, Helders P. Exercise therapy in juvenile idiopathic arthritis. Cochrane Database Syst Rev. 2008;2. 10.1002/14651858.CD005954.pub2 PMC8903819 18425929

[174] Taylor RS, Sagar VA, Davies EJ, Briscoe S, Coats AJS, Dalal H, Lough F, Rees K, Singh SJ, Mordi IR. Exercise-based rehabilitation for heart failure. Cochrane Database Syst Rev. 2014;4. 10.1002/14651858.CD003331.pub4 PMC6485909 24771460

[175] Thomas D, Elliott EJ, Naughton GA. Exercise for type 2 diabetes mellitus. Cochrane Database Syst Rev. 2006;3. 10.1002/14651858.CD002968.pub2 PMC8989410 16855995

[176] Ussher MH, Taylor AH, Faulkner GEJ. Exercise interventions for smoking cessation. Cochrane Database Syst Rev. 2014;8. 10.1002/14651858.CD002295.pub5 25170798

[177] Valentín-Gudiol M, Mattern-Baxter K, Girabent-Farrés M, Bagur-Calafat C, Hadders-Algra M, Angulo-Barroso RM. Treadmill interventions in children under six years of age at risk of neuromotor delay. Cochrane Database Syst Rev. 2017;7. 10.1002/14651858.CD009242.pub3 PMC6483121 28755534

[178] van der Heijden RA, Lankhorst NE, van Linschoten R, Bierma-Zeinstra SMA, van Middelkoop M. Exercise for treating patellofemoral pain syndrome. Cochrane Database Syst Rev. 2015;1. 10.1002/14651858.CD010387.pub2 PMC10898323 25603546

[179] Vloothuis JDM, Mulder M, Veerbeek JM, Konijnenbelt M, Visser-Meily JMA, Ket JCF, Kwakkel G, van Wegen EEH. Caregiver-mediated exercises for improving outcomes after stroke. Cochrane Database Syst Rev. 2016;12. 10.1002/14651858.CD011058.pub2 PMC6463929 28002636

[180] Voet NBM, van der Kooi EL. Riphagen, II, Lindeman E, van Engelen BGM, Geurts ACH: strength training and aerobic exercise training for muscle disease. Cochrane Database Syst Rev. 2013;7. 10.1002/14651858.CD003907.pub4 23835682

[181] White CM, Pritchard J, Turner-Stokes L. Exercise for people with peripheral neuropathy. Cochrane Database Syst Rev. 2004;4. 10.1002/14651858.CD003904.pub2 PMC12767756 15495069

[182] Wieland LS, Skoetz N, Pilkington K, Vempati R, D'Adamo CR, Berman BM. Yoga treatment for chronic non-specific low back pain. Cochrane Database Syst Rev. 2017;1. 10.1002/14651858.CD010671.pub2 PMC5294833 28076926

[183] Williams AD, Bird ML, Hardcastle SGK, Kirschbaum M, Ogden KJ, Walters JAE. Exercise for reducing falls in people living with and beyond cancer. Cochrane Database Syst Rev. 2018;10. 10.1002/14651858.CD011687.pub2 PMC6517115 30320433

[184] Williams MA, Srikesavan C, Heine PJ, Bruce J, Brosseau L, Hoxey-Thomas N, Lamb SE. Exercise for rheumatoid arthritis of the hand. Cochrane Database Syst Rev. 2018;7. 10.1002/14651858.CD003832.pub3 PMC6513509 30063798

[185] Yamamoto S, Hotta K, Ota E, Matsunaga A, Mori R. Exercise-based cardiac rehabilitation for people with implantable ventricular assist devices. Cochrane Database Syst Rev. 2018;9. 10.1002/14651858.CD012222.pub2 PMC6513315 30270428

[186] Yamato TP, Maher CG, Saragiotto BT, Hancock MJ, Ostelo R, Cabral CMN, Menezes Costa LC, Costa LOP. Pilates for low back pain. Cochrane Database Syst Rev. 2015;7. 10.1002/14651858.CD010265.pub2 PMC8078578 26133923

[187] Yang ZY, Zhong HB, Mao C, Yuan JQ, Huang YF, Wu XY, Gao YM, Tang JL. Yoga for asthma. Cochrane Database Syst Rev. 2016;4. 10.1002/14651858.CD010346.pub2 PMC6880926 27115477

[188] Young J, Angevaren M, Rusted J, Tabet N. Aerobic exercise to improve cognitive function in older people without known cognitive impairment. Cochrane Database Syst Rev. 2015;4. 10.1002/14651858.CD005381.pub4 PMC10554155 25900537

[189] Zainuldin R, Mackey MG, Alison JA. Optimal intensity and type of leg exercise training for people with chronic obstructive pulmonary disease. Cochrane Database Syst Rev. 2011;11. 10.1002/14651858.CD008008.pub2 PMC8939846 22071841

[190] Mok A, Khaw K-T, Luben R, Wareham N, Brage S (2019). Physical activity trajectories and mortality: population based cohort study. Bmj.

[191] Ekelund U, Brown WJ, Steene-Johannessen J, Fagerland MW, Owen N, Powell KE, Bauman AE, Lee IM (2019). Do the associations of sedentary behaviour with cardiovascular disease mortality and cancer mortality differ by physical activity level? A systematic review and harmonised meta-analysis of data from 850 060 participants. Br J Sports Med..

[192] Ekelund U, Steene-Johannessen J, Brown WJ, Fagerland MW, Owen N, Powell KE, Bauman A, Lee IM (2016). Does physical activity attenuate, or even eliminate, the detrimental association of sitting time with mortality? A harmonised meta-analysis of data from more than 1 million men and women. Lancet.

[193] Lear SA, Hu W, Rangarajan S, Gasevic D, Leong D, Iqbal R, Casanova A, Swaminathan S, Anjana RM, Kumar R (2017). The effect of physical activity on mortality and cardiovascular disease in 130000 people from 17 high-income, middle-income, and low-income countries: the PURE study. Lancet.

[194] Sattelmair J, Pertman J, Ding EL, Kohl HW, Haskell W, Lee IM (2011). Dose response between physical activity and risk of coronary heart disease: a meta-analysis. Circulation.

[195] Heyman E, Gamelin FX, Goekint M, Piscitelli F, Roelands B, Leclair E, Di Marzo V, Meeusen R (2012). Intense exercise increases circulating endocannabinoid and BDNF levels in humans--possible implications for reward and depression. Psychoneuroendocrinology.

[196] Horton R (2019). Offline: the gravy train of systematic reviews. Lancet.

[197] Guthold R, Stevens GA, Riley LM, Bull FC (2018). Worldwide trends in insufficient physical activity from 2001 to 2016: a pooled analysis of 358 population-based surveys with 1.9 million participants. Lancet Glob Health.

[198] Fletcher GF, Landolfo C, Niebauer J, Ozemek C, Arena R, Lavie CJ (2018). Promoting physical activity and exercise: JACC health promotion series. J Am Coll Cardiol.

