# Supplementary material for: Exercise/physical activity and health outcomes: an overview of Cochrane systematic reviews
Source: BMC Public Health. 2020 Nov 16;20:1724. doi: 10.1186/s12889-020-09855-3 (PMC7670795; doi:10.1186/s12889-020-09855-3)
Supplement: Supplementary file 1 — Supplementary Table 1. Main characteristics of included Cochrane systematic reviews evaluating the effects of physical activity/exercise on health outcomes (n = 150). Supplementary Table 2. Additional information from Cochrane systematic reviews of the effects of physical activity/exercise on health outcomes (n = 150). Supplementary Table 3. Conclusions from Cochrane systematic reviews “quote”. Supplementary Table 4. AEs reported in Cochrane systematic reviews. Supplementary Table 5. Summary of withdrawals/non-adherence. Supplementary Table 6. Methodological quality assessment of the included Cochrane reviews with AMSTAR-2. Supplementary Table 7. Number of studies assessed as low risk of bias per domain. Supplementary Table 8. GRADE for the review’s main comparison. Supplementary Table 9. Studies reporting quality of life outcomes as mean difference. [file 12889_2020_9855_MOESM1_ESM.docx]

**Supplementary Table 1.** **Main characteristics of included Cochrane systematic reviews evaluating the effects of physical activity/exercise on health outcomes (n=150)**

| **Reference** | **Assessed as up to date** | **Number of RCTs/**  **total N*** | **Subjects/condition** | **Gender distribution** | **Participants ages** | **Intervention (dose, frequency, intensity and duration)** | **Control** | **Confounders/**  **Co-interventions** |
| --- | --- | --- | --- | --- | --- | --- | --- | --- |
| (39) | Mar 2012 | 4/197 | Overweight or obese subjects with chronic asthma | / | Adults (18 and over) | A 10-week of combination of aerobic activities and resistance training. The dose of the interventions: 1 hour, at least 3 times a week | 1. Calorie restricted diet  2. Combination of the low-calorie diet and exercise program (the same as intervention group) | All groups received usual care for asthma,  exercise program |
| (40) | Jul 2016 | 44/4,781 | Overweight or obese adolescents | Females range: 33% to 77% | Range: 12 to 17.5 | Five trials focused solely on physical activity. The duration ranged from 6 weeks to 2 years, and follow-up: from 6 months to 2 years | No treatment, usual care or a concomitant therapy providing it was also included in the intervention group | Diet, education, behavioural components |
| (41) | Apr 2013 | 12/910 | Overweight or obese females during and after pregnancy (up to 24 months) | Females: 100% | Adults (18 and over) | Regular recreational exercises (e.g., walking, jogging, sports). Type, intensity, frequency, duration and timing varied between studies | Usual care | Diet |
| (42) | Jun 2016 | 10/300 | Participants after heart transplantation | Females:  < 25% | Mean: 54.4 | Mainly aerobic exercise (walking, running or cycling) but also anaerobic (resistance training, n=2). The duration ranged from 8 to 52 weeks, frequency (1-5 days a week), session length (28-50 minutes) and intensity (85-95% of maximal heart rate; 60-80% of maximal oxygen uptake; Borg rating of 11 to 14 | No exercise or in one study interval training with continuous moderate-intensity exercise | None in nine studies |
| (43) | Jul 2014 | 63/14,486 | Coronary heart disease | Females:  <15% | Mean range: 49.3 to 71 | Aerobic exercise (static cycling, walking or circuit training). The duration ranged from 1 to 48 months, frequency (1-7 sessions/week), session length (20-90 minutes) and intensity (50- 85% of max HR; 50-95% of maximum oxygen uptake; Borg rating of 11 to 15 | Usual care | Smoking cessation programs, psychotherapy, counselling, education, advice, psychosocial support, medication |
| (44) | Aug 2009 | 3/56 | Adults with Down syndrome | / | Range:17 to 65 | Walking/jogging and rowing, as aerobic exercise. The duration ranged from 10 to 25 weeks, session length (25 to 45 minutes, with 5 to 10 minutes warm-up in 2 studies) | Non-exercising group with unchanged lifestyles, any non-aerobic exercise programme | Behavioural components,  instruction in health education or health awareness |
| (45) | May 2016 | 2/54 | Chronic venous insufficiency | 17 Females, 37 Males | Mean: 62.3 | Study 1: 60 minutes of exercise twice a week (20 minutes in an exercise bath, and followed by 25 minutes of floor exercises)  Study 2: 1 hour of individualised therapy focusing on leg strengthening with progressed repetitions | No exercise | Compression stockings and  compression therapy |
| (46) | Sep 2002 | 6/224 | Elderly with chronic conditions (or at risk of developing ones) | / | At least 50 | Mainly high intensity supervised exercise (60 minutes of walking or jogging or cycling). Intensity: 73-88% of max HR, 3 times a week | ‘Centre based’ physical activity programs and usual care only | / |
| (47) | Apr 2015 | 13/1,190 | Knee or hip OA | 75% Females | Mean: 68 | Aquatic exercise (range of motion, strength, aerobics). Typically, session of 1 hour, twice a week (both supervised and unsupervised) for a total of 84 sessions, Mean duration: 12 weeks | Exercises (also land based), usual care, education, social attention (twice weekly for 1-hour sessions during a 12-week), telephone call, waiting list | / |
| (48) | Jul 2012 | 8/262 | Children and adolescents with asthma | Males range: 88-44% | 18 and under | In 7 studies swimming training ranged from 30 to 90 minutes, 2 to 3 times a week, over 6 to 12 weeks. In one study training involves 30 minutes, 6 times a week | Usual care and golf (n=1) | Inhaled corticosteroids, golf |
| (49) | Jan 2014 | 9/818 | Haematological malignancies | / | 18 and over | Mostly, walking intervention programmes plus usual care | No physical exercise (usual care alone group) | In one study erythropoietin was administered to 102 of 135 study participants |
| (50) | Jun 2016 | 13/839 | Fibromyalgia | 61.5% of studies included females only | Mean: 41 (range: 32 to 56) | Supervised aerobic exercises (walking, cycling, running, and doing low-impact aerobics and aquacise). The average length: 15 weeks (from 6 to 24 weeks); average frequency: 2-3 times a week for 35 minutes each session | Treatment as usual, wait list control, continuation of daily activities including physical activity | Medication |
| (51) | Dec 2016 | 4/150 | Fibromyalgia | Females | Middle-aged | Vibration exercise in a static position, on an oscillating platform or platforms | Placebo or sham intervention, usual care, wait list control and other active interventions including physical therapies or non-exercise interventions | / |
| (52) | Oct 2013 | 16/881 | Fibromyalgia | Females: 98.3% | Range: 40 to 50 | Aquatic exercise training with calisthenics | No exercise | / |
| (53) | Nov 2014 | 6/171 | Cancer patients or survivors | Boys 55%; Girls 45% | Mean: 8 (range: 5 to 12) | A home-based exercise programme with guidance from a therapist, included strength exercises. The duration ranged from 10 weeks to 2 years (15-60 minutes per session) | Usual care, no exercise | / |
| (54) | Jul 2014 | 3/207 | Cancer | Females: 100% | Mean: 53 | Dance/movement therapy;  Duration range: 3-12 weeks, once-twice a week (50-180 minutes per session) | Usual care alone or usual care combined with other therapies | / |
| (55) | Mar 2017 | 6/586 | Schizophrenia | / | Range: 18 to 60 | Yoga, duration: 2 weeks (at least 10 sessions) to 12 weeks (3 times a week) or one month (25 sessions) or 3 weeks (5 days a week) | Non-usual care incorporating physical postures with breathing exercises | / |
| (56) | Jan 2015 | 8/457 | Schizophrenia | / | Range: 18 to 60 | Yoga, duration: 2 weeks (at least 10 sessions) to 12 weeks (3 times a week) or one month (25 sessions) or 3 weeks (5 days a week) | Usual care control for schizophrenia or exercise: brisk walking, jogging, and exercise in standing or receiving no yoga | / |
| (57) | Mar 2017 | 3/193 | Schizophrenia | Primarily males (range not reported) | Range: 20 to 50 | Yoga (duration 3-8 weeks) | Usual care including medication and occupational therapy (n=1) | Pharmacotherapy (in two studies) |
| (58) | Aug 2016 | 11/638 | Pregnant females with a diagnosis of gestational diabetes mellitus | Females: 100% | Age range: 18-50 | Range from aerobic exercise to yoga. Duration: 8-10 weeks (20-50 minutes per session), Frequency: 3-5 times a week | Usual care | An individually prescribed diet, mindfulness eating |
| (59) | Jul 2005 | 34/2,276 | Fibromyalgia | Females: 96.4% | Mean age ranged from 27.5 to 60.2 | Cardiorespiratory endurance, muscle strength and/or flexibility as treatment mixed exercise plus medication, Aerobic exercise | No exercise | / |
| (60) | Mar 2013 | 5/219^a^ | Fibromyalgia | Females | Range: 37 to 60 | Moderate- and high intensity resistance training | Control or other physical activity | / |
| (61) | Aug 2017 | 95/138,164 | Elderly in care facilities or hospitals | Females: 75.3% | Mean: 83.5 | Active falls prevention intervention included exercises, medication, surgery | Usual care | / |
| (62) | Jan 2013 | 21/772 | Asthma | / | 8 or over, range:8 to 71 | Physical activity; Duration range: 6 to 16 weeks (20-30 minutes per session), frequency: 2-3 times a week | No intervention (wait-list), usual care | Patient education, medications |
| (63) | Jul 2011 | 3/104 | Head and neck cancer | Males (in n=2 studies) | Mean range: 52 to 61 (in n=2) | Active and passive stretching exercises, resistance exercises, proprioceptive neuromuscular facilitation | No treatment, usual care, placebo, sham exercises and pharmacological interventions, routine postoperative physiotherapy care in the hospital | / |
| (64) | Nov 2016 | 5/167 | Lung resection for non-small cell lung cancer | / | Mean range: 54 to 72.5 | Preoperative exercise training, aerobic exercises, breathing exercises, stretching. The frequency and duration of intervention varied from 3 times per day for one week, to 5 times per week for 4 weeks | No exercise training, usual care with no formal exercise training | / |
| (65) | Feb 2013 | 3/178 | Lung resection for non-small cell lung cancer | Males: 63%, females: 37% | Mean range:58 to 65 | Varied from twice-daily inpatient exercise for 5 days plus 12 weeks of home-based exercises to out-patient programmes (twice a week for 12 weeks) | Usual care | Pain medication |
| (66) | Oct 2009 | 4/114 | Pregnant females with gestational diabetes | Females:  100% | / | 3 times a week for 20 to 45 minutes | Usual physical activity, no exercise, insulin therapy or diet only | Dietary advice |
| (67) | Jul 2009 | 9/1,520 | Back pain | / | Range: 18 to 60 | General and specific exercises led by a physiotherapist, promoting physical activity | No intervention or sham ultra-sound therapy or usual care or educational booklet and a mini-back school | / |
| (68) | Mar 2015 | 7/923 | Overweight or obesity | / | 6 and under | Moderate and vigorous physical activity at least 60 minutes | Usual (or enhanced) care, education | Nutritional and behavioural counselling |
| (69) | May 2014 | 6/483 | Adult survivors of critical illness | Males: 62%, Females: 38% | 18 and over | Exercise rehabilitation or training initiated after intensive care unit discharge; duration range: 6-12 weeks (40-90 minutes per session) | Usual care | Usual care comprised of rehabilitation-related  interventions |
| (70) | Mar 2013 | 39/2,326 | Depression | / | Mean range: 22 to 87.9 | Aerobic, resistance exercise or mixed aerobic and resistance; duration range: 10 days-16 weeks | Placebo (n=2); no treatment, waiting list, usual care or self-management (n=17); treatment (n=6); stretching, meditation or  relaxation (n=6); occupational intervention, health education or casual conversation (n=4) | Cognitive behavioural therapy, pharmacological treatments |
| (71) | Jun 2015 | 42/1,453 | Hemiparesis after stroke | Males: 64% | Range: 37 to 87 | Constraint- induced movement therapy of the affected upper limb, range: 1-6 hours per day, 3-7 days per week, for 2-6 weeks | Usual care, no treatment or other physical exercises | / |
| (72) | Jan 2016 | 24/2,166 | Breast cancer | FemalesL  100% | Range: 44 to 62.9 (median= 54) | Different types of yoga,  Range: 6-12 weeks, 1-4 times weekly for 45-120 minutes | Attention control, wait-list control, treatment as usual, no therapy and any other active therapy | / |
| (73) | Mar 2011 | 56/4,068 | Cancer (mainly breast cancer) | / | 18 or over | Aerobic exercise, strength training and flexibility exercises ^b^ | No exercise, a usual care group or an alternative treatment or exercise regime for fatigue associated with cancer | / |
| (74) | Jul 2012 | 2/52 | Amyotrophic lateral sclerosis or motor neuron disease | / | Range: 41 to 80 | Progressive resistance or strengthening exercise, endurance or aerobic exercise ^b^ | No exercise or standard rehabilitation management | / |
| (75) | Feb 2015 | 2/40 | Respiratory diseases | Males: 100% | Range: 55 to 86 | Aerobic exercises or aerobic exercises and resistance training (2-3 times per week for 8 weeks in the outpatient setting) | Usual medical management (n=1), weekly telephone calls (n=1) | / |
| (76) | Mar 2014 | 5/733 | Symptomatic perimenopausal and postmenopausal females | Females: 100% | Range: 40 to 62 | Walking, cardiovascular conditioning, yoga ^b^ | No intervention, diet | / |
| (77) | Feb 2006 | 9/4,223 | Elderly medical patients | Females: ≈60% | 65 or older | Strength exercise, walking, exercises that were individually tailored by a physiotherapist ^b^ | Usual hospital care, control group, usual hospital physiotherapy or exercise/mobility care | / |
| (78) | Oct 2011 | 44/36,593 | Children and adolescents | / | Range: 6 to 18 | School-based intervention  educational, promotion of physical activity and fitness ^b^ | Usual, currently existing physical education programs | / |
| (79) | Aug 2017 | 4/690 | Critically ill adults in the intensive care unit | / | Mean or median range: 56 to 62 | Early mobilization or active exercise, cycle ergometer, active-assisted exercises, active ROM exercises, bed mobility activities, self-care tasks; frequency range: 1-3 per day (30-32 minutes per day) | Usual care (no mobilization/ active exercise) | / |
| (80) | May 2003 | 23/1,821 | Healthy children and adolescents | / | Range: 3-20 | Gross motor, energetic activity (e.g. running, swimming, ball games and out-door play of moderate to high intensity, or strength training). The minimum duration of 4 weeks | No intervention, waiting list, regular physical education classes | / |
| (81) | Apr 2015 | 11/1,817 | Parkinson’s disease | Male 60% | Over 60 | Physical exercise programmes consisted of aerobic training or strength training, or both ^b^ | Placebo, no treatment, usual care, or a waiting list control | Dopaminergic agents, psychostimulants and antidepressants |
| (82) | Feb 2014 | 1/39 | Haematological malignancies | Female: 63.2%, Male: 36.8% | Mean: 51 | Tibetan yoga (over 7 weeks, 1 session per week plus individual training) | Waiting list | / |
| (83) | Oct 2013 | 17/1,067 | Dementia | / | Over 65 | Exercises are the combination of aerobic-, strength-, or balance-training (from 2 weeks to 18 months, twice to week to everyday exercise; from 20 to 75 minutes) | Usual care with no additional interventions, social interactions | / |
| (84) | May 2013 | 54/3,537 | Knee OA | / | Adults (18 and over) | Land-based non-perioperative therapeutic exercise regimens aimed at relieving the symptoms of OA ^b^ | No exercise intervention, no treatment, waiting list | / |
| (85) | Feb 2013 | 10/549 | Hip OA | / | Adults (18 and over) | Land-based non-perioperative therapeutic exercise regimens aimed at relieving the symptoms of OA ^b^ | No exercise, no treatment or waiting list | / |
| (86) | Jan 2013 | 13/906 | Asthma | / | Adults (18 and over) | Breathing exercises, often as part of yoga training; duration range: 2-16 weeks (10-75 minutes per session); frequency: 3 times per day-3 times per week | Education or no intervention | Self-regulated bronchodilators  and injections; lifestyle modification and stress management |
| (87) | Mar 2015 | 32/2,626 | Breast cancer | Females 100% | / | Aerobic or resistance exercise interventions ^b^ | Usual care or no exercise | / |
| (88) | Nov 2011 | 7/488 | Osteoporotic fractures | Females ≈100% | Over 40 | Muscle strengthening or resistance training, aerobic exercises, balance training, Tai Chi, or individualized exercise prescribed by a physical therapist ^b^ | No exercise, no intervention or placebo | / |
| (89) | Mar 2012 | 159/79,193 | Elderly living in a community | Females 70% | 60 or over | Physical exercise (physical activity, Tai Chi, Comm-ex group, extended physiotherapy, Tele-ex and Home-ex groups) ^b^ | No exercise | / |
| (90) | Dec 2008 | 3/86 | Schizophrenia or related conditions | Males 67.4% | Mean: 43 | Walking or resistance exercise and aerobic training. Duration: 12-16 weeks | Usual care, waiting list, yoga with breathing practice and relaxation techniques | / |
| (91) | Jul 2014 | 11/904 | Acute respiratory conditions | Females range: 52-100% | Range: 18 to 85 | Aerobic exercise including bicycle riding, treadmill or walking; Duration range: 7 days to 12 months (30-45 minutes per session; frequency: 3-7 times a week) | Usual care, no exercise, stretching | Didactic instruction, medications |
| (92) | Nov 2015 | 6/599 | Participants before receiving influenza vaccination | Females range: 50 to 82% | Range: 18 to 80 | Walking or endurance exercises and resistance activities (25-50 minutes per session) | Quiet rest, sitting, reading or no exercise | / |
| (93) | May 2014 | 3/136 | Adult asthmatics | Males 55.9% | Mean: 34 | Water-based exercise (40-60 minutes, 3 to 5 times a week; for 10 to 24 weeks in 2 studies | Land-based exercise | Medication and  asthma education |
| (94) | May 2014 | 27/2,485 | Acute, subacute or chronic neck disorders | / | 18 or over | Exercise, exercise combined with another intervention ^b^ | Placebo treatment, no treatment, manipulation, education/advice, acupuncture, massage, heat or medications | / |
| (95) | Dec 2016 | 21/1,400 | Intermittent claudication | Males 65% | Mean: 66 | Supervised exercise therapy or home-based exercise therapy (3 sessions per week). Follow-up ranged from 6 weeks to 2 years | Walking | / |
| (96) | Dec 2003 | 4/206 | RA | / | Range: 16 to 72 | Tai Chi 60 or 90 minutes, once or twice per week for 8-10 weeks | No therapy, sham therapy | Hot compresses, massage, herbs, dance program |
| (97) | April 2012 | 5/922 | Pregnant females | Females 100% | Range: 18 to 45 | Exercise and lifestyle management for pregnant Females for preventing GDM (land aerobic and aquatic activities session, cycling) ^b^ | Usual antenatal care with normal daily activities (n=4) | / |
| (98) | Nov 2013 | 11/800 | Healthy adults and those at moderate to high risk of CVD | / | Range: 18 to 70 | Yoga, duration range: 3-8 months | No intervention, minimal intervention | / |
| (99) | Dec 2013 | 13/1,552 | Healthy adults or adults at high risk of CVD | / | 18 or over | Tai chi, duration range: 3-12 months | No intervention, minimal intervention | / |
| (100) | Nov 2014 | 11/1,369 | Adults at high risk of CVD | Primarily males (range not reported) | 18 or over | Qigong (20-60 minutes per session; frequency: twice per day-at least 3 times per week) | No intervention, medications | Antihypertensives |
| (101) | Aug 2017 | 8/399 | Traumatic brain injury | Primarily males (range not reported) | / | Fitness training included exercising on a fixed cycling machine, in water, on gym equipment such as a treadmill, home-based exercise, and a fitness group in the military according to ACSM recommendations. Duration: at least 3 times a week for at least 20 minutes | No exercise (n=2), no intervention, usual care | / |
| (102) | Oct 2004 | 61/6,390 | Non-specific low-back pain | Males: 49% | Mean: 41 (range: 39 to 42) | A routine practice (therapy) or physical training (directed to physical health) | No treatment, placebo treatment, other conservative therapy, another exercise group | / |
| (103) | May 2011 | 21/1,490 | Urinary incontinence | Females 100% | Mean: 50 (range: NR) | Pelvic floor muscle training; mean duration: 12 weeks (15-45 minutes); frequency: daily to 3 times a week | Alternative approaches to pelvic floor muscle training e.g., Pilates | In 13 trials the experimental group received some additional intervention e.g., phone calls |
| (104) | Oct 2014 | 45/2,250 | Multiple sclerosis | / | 18 or over | Endurance training (23 interventions), muscle power training (9 interventions), task-oriented training (5 interventions), mixed training (15 interventions), or other (e.g. yoga; 17 interventions) | No exercise therapy, other interventions, wait list control | / |
| (105) | May 2010 | 45/1,863 | Chronic kidney disease and kidney transplant patients | / | Age (mean): 36 to 71 | Regular physical exercise training (e.g., cardiovascular, resistance training, yoga); duration: 2 to 18 months (20-110 minutes per session), intensity was varied | No exercise or co-intervention, i.e. physical exercise training plus erythropoietin treatment versus erythropoietin treatment | / |
| (106) | Sep 2017 | 12/5,238 | People at increased risk of developing type 2 diabetes mellitus | / | Mean age range: 45 to 63 | Diet plus physical activity. Duration: 2 to 6 years | Usual care, no treatment | / |
| (107) | Feb 2010 | 12/2,577 | delayed‐onset muscle soreness after exercise | / | Mean: 39.9 | Any pre-exercise or post-exercise stretching technique designed to prevent delayed-onset muscle soreness. Ten studies were conducted. | No stretching, no recovery procedures, control leg | / |
| (108) | Nov 2004 | 19/3,584 | Low-back pain | / | Mean age range: 18 to 70 | A back school was defined as “consisting of an educational and skills acquisition program, including exercises, in which all lessons were given to groups of patients and supervised by a paramedical therapist or medical specialist” | Exercises, manipulation, myofascial therapy, advice, placebo or waiting list controls | / |
| (109) | Oct 2011 | 16/1,233 | Chronic obstructive pulmonary disease | / | Mean age range: 51 to 73 | Breathing exercises, either supervised or unsupervised. The duration of the interventions varied from a single session to 20 months. The number of supervised sessions ranged from zero to three times per week | No breathing exercises, another intervention (e.g. oral placebo), another intervention versus no breathing exercises, usual care | / |
| (110) | Feb 2011 | 94/9,821 | Older people | / | Mean age range: 60 to 75 | Coordination and functional tasks, strengthening exercise, power training, Tai Chi, dance, yoga, walking or cycling, balance training, vibration training | Usual activities (n=64), education sessions (n=9) | / |
| (111) | Dec 2010 | 43/4,320 | Healthy postmenopausal females | Females: 100% | Range: 45 to 70 | Static and dynamic weight bearing, walking, Tai chi, jogging, jumping, running, dancing and vibration training, strength training, Frequency: 2-3 times per week | Usual therapy (e.g. usual activity or placebo with or without pharmacological consumption) | / |
| (112) | June 2009 | 8/575 | Rheumatoid arthritis | Females: 79.9% | Mean:  60.3 (range: 48 to 67) | Short-term aerobic capacity and muscle strength training (land based), bicycling and strengthening exercises, bicycle and weight-bearing exercises (2 to 3 times per week; 3-12 months) | No exercise, ROM plus isometric exercises | / |
| (113) | April 2018 | 21/2,372 | Chronic knee or hip joint pain from OA | Females: 72% | Mean:  66 (range: 57 to 79 | 1. Combined strength training with different forms of aerobic exercise, Tai Chi, Water-based exercise  (Duration range: 4-24 weeks; intensity: 20-60 minutes; frequency: 0.5-5 times per week) | No treatment, waiting list or no exercise | / |
| (114) | Feb 2013 | 1/45 | Dysfunctional breathing/hyperventilation syndrome (DB/HVS) | / | / | Relaxation therapy and breathing exercise: 8 one-hour training sessions twice weekly | Relaxation therapy | Partially supervised |
| (115) | Oct 2015 | 12/695 | Cardiac or major abdominal surgery | Females: 37% (range 20 to 100) | Mean:  64 (range 34.8 to 71.1) | Simple preoperative inspiratory muscle training (IMT) with threshold device or mixed training program that consisted of IMT with threshold-loading device and exercise training of trunk and extremity. (duration: 2-4 weeks; 1-3 times a day, 5-7 times per week, 15-30 minutes per session) | Usual care or no treatment, sham training, home-based exercise advice | Partially supervised |
| (116) | Oct 2015 | 30/2,878 | Community-dwelling people not restricted to medical condition | Females: 73% (range 44 to 100%) | Mean: 76 (range: 68 to 85) | 25% classified as 3D (Tai Chi, Yoga); 53% classified as gait, balance, co- ordination, functional tasks; and 22% classified as strength and resistance-based interventions. Duration 12 to 26 weeks; 1 to 4 or more times a week) | No intervention and alternative no exercise | Partially supervised |
| (117) | Jan 2010 | 14/1,014 | Healthy pregnant Females | Females: 100% | / | (Aerobic) exercise or hydrotherapy; Duration: 3-5 times per week, 8 to 32 weeks, 15 to 60 minutes per session) | No exercise, exercise with other intensity, relaxation and stretching sessions, normal activity without aerobic exercise | Partially supervised |
| (118) | Jan 2018 | 63/5,761 | Females with breast cancer | Females: 100% | Mean:  54 (range: 46 to 63) | 11% of trials included a separate resistance training condition with no form of aerobic activity; 33% of trials involved an intervention arm that combined aerobic activity and resistance training; 44% of trials consisted of an aerobic activity-only condition; 13% included a yoga-only arm and one study provided each of the following intervention arms: Pilates only, (tai chi only and qigong (similar to tai chi) only, duration: 2-7 days per week; 4 weeks to 24 months; 15 minutes to 95 minutes per session) | Usual care, no intervention | Partially supervised |
| (119) | Dec 2017 | 32/1,835 | Atherosclerotic disease | Females: 39% (range 0 to 51.5%) | Mean:  67 years (range: 53 to 76) | Types of exercise varied from strength training to pole striding and upper or lower limb exercises; duration: 2 per day to 2 per week; 15 to 90 minutes per session; 14 days to 2 years) | Usual care, medication | Mostly supervised |
| (120) | April 2017 | 8/1,518 | Chronic fatigue syndrome | Females:  76% (range: 65 to 83.3) | Mean:  40 years (range: 33 to 45) | Variations of aerobic exercise therapy such as walking, swimming, cycling or dancing provided at mixed levels in terms of intensity of the aerobic exercise from very low to quite rigorous, or anaerobic exercise; duration 6 per week to every 2 weeks; 5 to 45 minutes per session; 12 to 26 weeks) | Passive control; e.g. treatment as usual, relaxation, flexibility) or CBT, cognitive therapy, supportive listening, pacing, pharmacological treatment and combination treatment | Mostly supervised, strategies to achieve behavioral changes and to improve self-help (such as leaflets), (placebo) drugs |
| (121) | May 2006 | 16/1,191 | Children and young people aged 0 to 20 years, with or without anxiety and depression | Females:  42% (range: 0 to 100%) | Mean:  18 (range 13 to 20) | Different aerobic exercise such as walking, running, aerobics or weightlifting; Duration: 2 to 4 times per week; 5 to 90 minutes; 6 to 40 weeks | No intervention, low intensity, psychosocial interventions | Partially supervised, nutritional education and sessions with behavioral modifications |
| (122) | July 2014 | 5/135 | Intermittent claudication | / | Range: 62 to 71.7 | Supervised walking exercise; duration: 6 to 24 weeks | Cycling, strength training, and upper-arm ergometry | Supervised |
| (123) | Dec 2017 | 2/72 | Stroke survivors (Mean time post stroke ranged from 51 months to 81.6 | Females:  43% (range 0 to 73) | Mean: 60 (range: 56 to 64) | Yoga (8 to 10 weeks; group sessions weekly to bi-weekly 60 to 90 minutes per group class and daily 40 minutes home practice to 3 or more yoga plus groups per week with 20 minutes) | No intervention, waiting list | Supervision / classes by yoga instructors |
| (124) | Nov 2012 | 38/1,896 | Ankle fracture | Females:  52% (range 30 to 88%) | Mean: 42 (range: 26 to 57) | Different interventions of participants after conservative orthopedic management or during the period of immobilization, weight-bearing during the immobilization period, active or passive ankle range of motion, strengthening and functional exercises | No exercise, usual care | Partially supervised |
| (125) | July 2009 | 121/6,700 | Older people, resident in institutions or at home in the community | / | Mean age: 60 to >80 | Progressive resistance strength training: high intensity programs; with specialized exercise machines or low-intensity to moderate-intensity training, with most using elastic tubing or bands; duration: 2 to 7 times per week; 2 to 104 weeks | No exercise; usual care | Mostly supervised |
| (126) | Feb 2018 | 7/581 | Coronary heart disease with stable angina | Females:  13% (range: 0 to 28.8%) | Mean: 59 (range: 50 to 66) | Exercise-based cardiac rehabilitation; duration: 6 weeks to 12 months; 11 to 90 minutes per session, daily sessions | Usual care, percutaneous coronary intervention | 3 studies included complex interventions such as additional educational interventions and behavior change techniques; and medication |
| (127) | Dec 2018 | 11/1,067 | Cancer (7 studies investigating breast cancer, one stomach cancer, 2 combined breast and colon cancer; another study included 21 cancer types) | Females:  89% (range: 31 to 100%) | Mean: 51 (range: 40 to 70) | Exercise interventions (containing cycle ergometer, strength exercises on machines, body awareness and restoring training and massage, yoga exercises, treadmill and bicycling, walking, walking and home-based exercises for strength balance and mobility or walking and strength-resistant exercises; duration: 5 weeks to 12 months; 15 to 90 minutes per session; 2 to 6 times per week | Usual care | Supervision; encouragement; telephone conversations |
| (128) | Feb 2016 | 3/197 | Acute non-specific low back pain | / | Median: 36 (range: 31 to 38.4) | Motor control exercise: specific exercises for stabilization of muscles; manual therapy techniques; instructions on repeated range of motion exercises; program of trunk strengthening and stabilization exercises; exercises were designed to re-educate the multifidus muscle in its stabilizing role; duration: 4 to 6 weeks; daily to twice weekly | Other types of exercise including general exercise; specific exercise; spinal manipulative therapy | Partially supervised |
| (129) | April 2016 | 3/112 | Children with asthma | Females: 58% (range 42 to 72%) | Mean 10 (range 10 to 11) | Breathing exercises as part of a more complex intervention; duration: twice per day to twice per week; 2 to 30 days; 50 to 60 minutes per session | Placebo, educational program and doctor appointments | All breathing exercises were parts of complex interventions |
| (130) | March 2018 | 18/2,384 | Children and adolescents with overweight or obesity | Females: 50% (range 19 to 72%) | Mean 11 (range 4 to 17) | Eight studies delivered physical activity interventions (group aerobic exercise, group co-ordination skills exercise, physically active academic lessons, extracurricular individual or small group physical activity; duration:10 weeks to 18 months; 3 to 5 per week; 20 to 40 minutes per session | Waiting list, attention placebo program, same program with different intensity | Mostly supervised |
| (131) | Nov 2016 | 15/425 | Chronic obstructive pulmonary disease (COPD) | Females: 27 % (range 0 to 61%) | Mean age 67 (range 57 to 72) | Upper limb exercise training including resistance training and endurance training and a combination of both supported and unsupported endurance training; duration: 1 to 5 times per week; 4 to 16 weeks | No training or sham intervention, lower limb training alone, another type of upper limb training | The upper limb training was in addition to a prescribed lower limb training program; supervision |
| (132) | Dec 2013 | 5/176 | Chronic obstructive pulmonary disease (COPD) | Females: 38.5% (range 0 to 73%) | Mean 66 (range 57 to 73) | Water-based exercise training: Upper limb and/ or lower limb endurance exercise training, strength training and use of weights or floats; duration: 4 to 12 weeks; 2 to 3 times per week; 35 to 90 minutes per session | Land-based exercise training, no exercise training | Supervised |
| (133) | June 2010 | 24/2,132 | Upper limb dysfunction after breast cancer treatment | Females: 100% | Range 46.3 to 62.1 | Exercise intervention: home or group-based exercise programs; duration: 14 to 1 per week; 1 to 12 weeks; 30 to 60 minutes per session | Delayed upper-limb exercise intervention, usual care | Partly supervised |
| (134) | June 2017 | 70/8,461 | Overweight or obese children | Females: <30-100% | Median age: 10 (range 6.2 to 11.9) | Diet, physical activity and behavioral interventions; duration: 10 days to 2 years | No treatment, usual care | Supervised |
| (135) | Feb 2015 | 3/147 | Depression | Females: 66% (range 37.5-100%) | Mean 27 (range 16 to 32) | Dance movement therapy in group format; duration: 4-12 weeks; 2 to 5 times per week; 45 minutes to 2 hours per session | No treatment, usual care | Supervised |
| (136) | Jan 2010 | 2/45 | Females at risk of pre-eclampsia | Females: 100% | / | Moderate intensity regular aerobic exercise; duration: 30-45 minutes per session; 3-4 times per week; 10 weeks | Usual physical activity | Supervision, dietary counselling |
| (137) | Jan 2011 | 4/94 | Stroke | / | Mean: 58 (range 50 to 67) | Water-based exercises | No exercise, other exercise; or only 1 component from multicomponent intervention (land therapy) | / |
| (138) | Nov 2012 | 5/309 | Traumatic spinal cord injury | Females: 28% | Range: 18 to 68 | Locomotor training: robotic-assisted device; bodyweight supported treadmill training; duration: 2-5 times per week; 4 to 12 weeks; 30 to 60 minutes per session | Other types of exercise | / |
| (139) | Aug 2017 | 56/3,105 | Stroke | Females: 0 to 75% | Mean: 60 | Treadmill training (with and without) and body weight support | Another physiotherapy intervention; no intervention; sham intervention; treadmill training only | Partially body weight support |
| (140) | Aug 2012 | 40/3,694 | Cancer survivors (including breast, colorectal, head and neck, and other cancer) | Females: 22 to 100% | Mean: 39 to 68 | Exercise interventions: mode of the exercise intervention included strength training, resistance training, walking, cycling, yoga, Qigong, or Tai Chi; duration: 3 weeks to 1 year; once per week to daily; 20 to 90 minutes | Usual care or no intervention | Mainly supervised |
| (141) | Aug 2012 | 56/4,826 | Cancer during active treatment (breast, prostate, gynecologic, hematologic, and other cancer) | Females: 0 to 100% | Mean: 40 to 71 | Exercise interventions: included walking by itself or in combination with cycling, resistance training, or strength training; resistance training; strength training; cycling; yoga; or Qigong; duration: 3 to 26 weeks; 12 to 120 minutes per session, once per week to daily | Usual care or no intervention | Partly supervised |
| (142) | Oct 2002 | 1/43 | Older people (>60) with sleep problems | / | Mean: 62 | Physical exercise: community-based exercise training (low impact aerobics; brisk walking; duration: 16 weeks; moderate intensity; 4 per week; 30 to 40 minutes per session | Wait-list | / |
| (143) | Jan 2017 | 6/206 | Pulmonary hypertension | Females: 67% (range 51 to 100%) | Mean: 54 (range 47 to 57) | Exercise-based rehabilitation programs: incorporated both upper and lower limb exercise; duration: 2-7 times per week; 3-12 weeks; 30-90 minutes | Usual care, education alone, no exercise | Partially supervised |
| (144) | June 2015 | 65/11,444 | Pregnancy | Females: 100% | Range: 19 to 35 | Diet or exercise or both; water-based or land-based exercise; including walking; strength exercise; aerobic and relaxation exercise; duration: 9 to 33 weeks; 30 to 60 minutes per session; 2 to 7 times per week | Usual care | Partly supervised; partly including diet and pedometer for monitoring |
| (145) | June 2016 | 12/984 | Chronic obstructive pulmonary disease (COPD) | Females range 8 to 44% | Mean range: 61 to 74 | Tai Chi alone or in addition to another intervention; duration: 6 weeks to 1 year; 15 to 60 minutes per session; 5 to 7 days per week | Usual care; breathing exercise alone; exercise alone | Partly supervised |
| (146) | July 2012 | 21/1525 | Fecal incontinence in adults | Females range 50 to 100% | Mean age range: 30 to 74 | Anal sphincter exercises/pelvic muscle floor training (PFMT) with or without biofeedback; duration: 4 weeks to 9 months; 1 per week to twice daily; 20 to 120 minutes per session | No intervention; any other treatment such as electrical stimulation, sacral nerve stimulation, surgery, education, habit re-training; exercise with or without biofeedback; anal sphincter exercise/pelvic muscle training without biofeedback or with another type of biofeedback | / |
| (147) | Oct 2004 | 7/294 | Adults living with HIV/AIDS | Females: 15% (range 0 to 100%) | / | Progressive resistive exercise (PRE) interventions; PRE only; or a combination of PRE and aerobic exercise; duration: 6 to 16 weeks; 3 times per week | No exercise | Supervised |
| (148) | Aug 2010 | 14/545 | Adults living with HIV/AIDS | Females: 30% (range 0 to 100%) | / | Aerobic exercise (constant or interval); duration: 3 times per week; 5-24 weeks; 20-120 minutes per session; low to heavy intensity | No exercise | Supervised |
| (149) | Jan 2017 | 7/534 | Hand OA | Females: 90% | Mean age range: 60 to 81 | Exercise aimed to improve muscle strength and joint stability or function; duration: 4 times daily to 2 times per week; moderate to vigorous; 6 weeks to 12 months | No exercise including sham intervention, other type of exercise | Partly supervised |
| (150) | Aug 2014 | 32/1,836 | Adhesive capsulitis (frozen shoulder) | Females: 54% | Mean: 55 | Manual therapy and exercise: Maitland’s mobilization techniques Codman’s pendulum exercises, active and passive ROM exercises, pulley exercises and shoulder wheel exercises; duration: 1 to 7 per week 1 to 18 weeks | Glucocorticoid injection; sham ultrasound, oral NSAID; another type of exercise (or exercise with other additional components of interventions), no intervention | Supervised; |
| (151) | June 2016 | 60/3,620 | Rotator cuff disease – mean duration of symptoms was 11 months | Females: 52% | Mean: 51 | Strengthening exercise, stretching exercise, range of motion exercise, progressive resistance training; duration: 1 to 24 weeks; 1 to 7 per week | no intervention; usual care | Supervised |
| (152) | Jun 2012 | 16/741 | Carpal tunnel syndrome | Females: 84% | Mean age range: 27 to 52 | Exercise mobilization intervention other than nerve mobilization (for example yoga or chiropractic treatment); duration: 3 weeks to 6 months; 5 per day to 1 per week; 10 minutes to 90 minutes per session) | No treatment, another mobilization technique, non-surgical intervention | Partially supervised |
| (153) | Jan 2017 | 2/50 | Epilepsy (all types) | Females: 72%  Range: 30 to 100% | Mean 22.9  Range: 19.7 to 25.8 | Any type of classical Indian yoga  The treatment protocols consisted of 12 hours of professional therapy distributed in 2 individual sessions, 2 group sessions during a 5-week period, and booster sessions at 6- and 12-months post-treatment | Acceptance and Commitment Therapy (ACT)  In 2 sessions (individual session and group session),  No intervention | The use of antiepileptic drugs was permitted in both the treatment and the control groups |
| (154) | July 2016 | 6/326 | Advanced head and neck cancer | Percentage female 20.55%  Range 4 to 39% | Mean 59.4  Range 54 to 63 | Therapeutic swallowing exercises (4 -5 swallowing exercises as 5-10 repetitions over 4 cycles, each of 10-15 minutes’ duration. 5-7 days a week for 6 weeks, practicing 3-5 times per day.  Partly additional home practice: 3 times/day for at least 5 minutes (weekly, for 30 weeks) with weekly telephone monitoring | Usual care or Sham (about 10 repetitions over 4 cycles, each of 10 minutes’ duration) | Curative treatments (primary or postoperatively) chemo or radiotherapy |
| (155) | Aug 2017 | 15/487 | Cystic fibrosis | / | Range 6 to 35.5 | Aerobic and resistance: daily activity to exercises 3-5times per week for about 20 -  60 minutes for 13 days to 24 months | Normal daily activities/  Free access to a fitness centre for 1 year/ Standardized CF protocol/ Inspiratory muscle training only at a low intensity/ Normal hospital treatment | / |
| (156) | June 2014 | 6/656 | Knee or hip OA | Females: 66.9% (range 44.7 to 100% | Mean 61.3  Range 54.1 to 71 | Exercise program with different levels of strength resistance. (High-low resistance), frequency: 2-5 times/week; 8-24 weeks; session duration: 25-60 min | No exercise | / |
| (157) | July 2012 | 1/45 | Schizophrenia | Females: 51.1%  (range – NA) | Range 20 to 55 | Body-oriented psychological therapy (BPT) + routine care: Frequency: 60-90 minutes/session,  20 sessions over 10 weeks | Usual care | / |
| (158) | March 2004 | 9/260 | Multiple Sclerosis (MS) | Females 67% (range 30 to 85%) | Mean 41.1 (range 39.9 to 49.8) | Outpatient supervised general aerobic, strengthening and flexibility exercise training, outpatient physiotherapy, inpatient bicycle exercise training, inpatient physical rehabilitation programme or home-based, lower-extremity resistance training, 30-60 min., 1-5 times per week, for 3-15 weeks | No physical intervention, participants were asked to maintain their normal activity level or self-executed exercise program at home | / |
| (159) | July 2016 | 6/421 | Adults with atrial fibrillation or those who had been treated for atrial fibrillation | Females: 28.8%  Range 12 to 53% | Mean 62.4 Range 59 to 70.2 | Different physical exercises like aerobic training, with and without cool down and warm-up periods, or Qigong (slow movements with a focus on breathing), or cycling, walking on stairs, running, fitness training on physioballs, and interval training, or graduated cardiovascular training.  15-90 min., 1-2 times a day, 3-7 sessions/week for 8-16 weeks | No physical activity, normal daily activity habits | 2 hours of training on best practices for cardiovascular risk management (1 study) |
| (160) | March 2011 | 2/154 | Adolescents with idiopathic scoliosis (AIS) | Female 61.7% (range 53.8 to 70.3%) | Mean 13.75  Range 12.4-15 | Individual education (1.5 hours session every 2-3 months), and gymnastic exercises performed by the patient daily to twice a week at home or at a gym | Usual physiotherapy | Electro-stimulation on the lateral body surface by a therapeutic apparatus |
| (161) | June 2016 | 29/926 | Children, adolescents and adults with cerebral palsy | Female range: 33 to 71% | Mean age range:5 to 45 | Resistance training/ physical education, physical therapy/ occupational therapy/ aerobic exercises such as cycling, wheelchair driving, running, swimming/ training on a “flying saucer” and mat exercises/ exercises for the hip flexors, hip extensors, hip abductors, knee flexors and knee extensors bilaterally  20- 70 min,  2-3 times per week,  6 to 20 weeks | Physical  education, physical therapy, occupational therapy, speech therapy, and typical school  classes,  conventional therapy/ normal activity | Ongoing medication therapy |
| (162) | April 2015 | 29/2,431 | Chronic non-specific low back pain | / | / | 12- 18 sessions of an exercise program, 30-180 minutes, duration of 6 -12 weeks, 2-4 sessions per week:  Motor control exercise, general exercise, specific spinal stabilization exercise (spinal stabilization physiotherapy), individual physiotherapy, pain management, stabilization exercise | Conventional exercise/conventional treatment, general exercise | / |
| (163) | Feb 2015 | 58/2,797 | Stroke patients | Females: 12.5 to 85% | Mean age range 47 to 76 | Resistance interventions (13 trials, 432 participants), mixed training interventions (17 trials, 957 participants):  resistance training, treadmill training:  4-16-weeks intervention,  3-5 times/week,  each session lasting 30- 90 minutes | No intervention | / |
| (164) | March 2013 | 11/324 | Preterm infants with low bone mineralization or metabolic bone disease | / | Mean Gestational Age: 28 to 33 weeks | Systematic physical activity programs consisting of extension and flexion, range-of-motion exercises of the infant’s upper and lower limbs, administered for several minutes at a time several times a week for at least two weeks | No organized physical activity programs | / |
| (165) | Nov 2013 | 4/823 | People with high cardiovascular risk | - | - | Exercise: treadmill, aerobic exercise (walking, jogging, dance, football, basketball, kick boxing etc.), advice on lifestyle, fitness club; duration: 20- 60 minutes, 2-4 times a week, 16 weeks - 6 months | Advice on lifestyle, no exercise or alternative intervention, diet intervention | Participants received counselling from a nutritionist |
| (166) | Jan 2006 | 43/3,476 | Adults with overweight or obesity | Females: 0 to 100% | Mean age range 30.9 to 66 | Exercise (cycle ergometer, aerobic,  walking or aqua jogging), 4-5 times a week, for 20-60 minutes a session, 6-52 weeks; or low fat, low calorie diet plus structured exercise | Light exercise by flexibility stretching, slow cycling,  slow walking,  Exercise without diet, diet only, no intervention | Diet |
| (167) | Nov 2016 | 23/8,918 | Pregnant Females, for preventing Gestational Diabetes Mellitus (GDM) | Females: 100% | / | Moderate-intensity activity/ mild physical activity program, duration: 8-12-weeks, 3-7 times per week, 30-40 minutes | Routine pregnancy care | Diet |
| (168) | March 2015 | 2/148 | Patients after heart valve surgery | Females: 35% | Mean: 35.6 | Combined physical exercise, breathing exercises and psychological intervention: Physical exercise (limb stretch/joint exercises and aerobic exercises), aerobic exercise 2 - 7 times per week; duration: 3 to 5 minutes limb stretch/joint exercises and 20 to 30 minutes aerobic exercise/session or 3-4 hours simple exercises; 4 weeks | No intervention | / |
| (169) | Sept 2013 | 5/113 | Asthma | Females: 59% | Mean: 32 | Inspiratory muscle training; duration: 10 to 30 minutes per session; 3 to 25 weeks; 2 per day to 3 per week | Sham intervention, no intervention | / |
| (170) | July 2009 | 9/499 | Chronic stroke patients with mobility deficits (the average time since stroke onset ranged from 6.1 months to 64 months) | Females: 30 to 50% | Mean range: 57 to 74 | Physical therapy gait training: re-education; circuit program; cardiorespiratory exercise; progressive resistance training; duration: 1 to 3 per week; 4 weeks to 6 months; 30 to 60 minutes | No treatment, sham exercise (seated exercises for the upper extremities) | Supervised |
| (171) | Dec 2016 | 8/233 | Hemophilia | Females:0% | Range 8 to 49 | Exercise included resistance exercises, isometric exercises, bicycle ergometry, treadmill walking and hydrotherapy; duration: 4 to 12 weeks; 2 to 5 per week; 40 to 115 minutes per session | No intervention, no exercise, other type of exercise, exercise plus electro physical modality | / |
| (172) | April 2008 | 3/212 | Juvenile idiopathic arthritis | Females: 56% | Age range to19 years | Exercise therapy: combined hydrotherapy and land-based physiotherapy; high intensity aerobic training, aquatic training; duration: 60 minutes per session; 10 weeks to 6 months; 1-3 times per week | Land-based physiotherapy alone, low intensity aerobic training | Partially supervised |
| (173) | April 2014 | 33/4,740 | Heart failure | Females: 13% (median) | Mean age range: 51 to 81 | Exercise-based rehabilitation: All trials evaluated an aerobic intervention and 11 also included resistance training; duration: 15 to 120 minutes per session; 1 to 7 per week; 15 to 120 weeks | Usual care; education; continue their previous lifestyle; disease management | Supervised, group based educational session; preparation of low-fat meals, strategies from social-learning theory to improve adherence |
| (174) | March 2006 | 14/377 | Type 2 diabetes mellitus | Females: (range 0 to 100%) | Mean age range: 45 to 67 | Exercise: Progressive resistance training or moderate aerobic exercise such as walking or cycling, or controlled endurance training combined with muscle strength training; duration: 8 weeks to 6 months; 30 to 120 minutes per session; 1 to 7 per week) | No exercise (e.g. weight loss diet alone, no intervention) | Supervised |
| (175) | Aug 2014 | 20/5,870 | Smoking cessation | Females: (range 0 to 100%) | Mean age range: 17 to 59 | Exercise: Mostly group based cardiovascular-type exercise; home-based exercise, telephone-based physical activity counselling, and web-based fitness program, or individual program of resistance exercise (i.e. weight training) or yoga intervention (15 to 60 minutes per session; every second to third week to 3 per week; 4 to 19 weeks) | Smoking cessation program without exercise components | Mostly supervised, financial incentives to attend, pedometer |
| (176) | July 2017 | 7/175 | Risk of neuromotor delay | Females: 30% | Mean age 13 years | Treadmill intervention; duration: 6 to 20 minutes per session; 2 to 6 times per week; 6 to 12 weeks | Different type of intensity, no treadmill, treadmill with vs treadmill without orthoses | Supervised |
| (177) | Jan 2015 | 31/1,690 | Patellofemoral pain syndrome (duration of complaints ranged from four weeks to nine years) | Females: 100% | Mean age range: 18 to 41 | Exercise: Kinetic chain exercise, hip and knee exercise; duration: range 3 weeks to 4 months; 2 per day to 3 per week | No treatment, health educational material, other conservative interventions (e.g. taping), different exercises | Partly supervised, medial patellar taping, education, ultrasonic therapy, written information about patellofemoral pain syndrome and general instructions for home exercises |
| (178) | Dec 2016 | 6/456 couples | Stroke (mean time since onset of symptoms ranged from 15 days to 10 years) | Females: 39% | Mean: 60 | Caregiver-mediated exercises: 2 trials aimed at lower body, 5 at upper body, 2 both at upper and lower body (partly in addition to usual care); duration: 2 per week to daily; 30 minutes to 3 hours per session; 14 days to 6 months | Usual care (educational therapy and conventional physiotherapy), no intervention | Supervision, exercise book |
| (179) | July 2013 | 5/170 | Muscle disease | - | - | Strength training and aerobic exercise training: two strength training trials, one aerobic exercise trial and two strength training combined with aerobic exercise trials | No training | Supervised |
| (180) | Oct 2004 | 3/82 | Peripheral neuropathy | / | Range: 16 to 80 | Exercise: Strengthening exercise, lower limb strengthening and balance exercise, home exercise; duration: 7 per week; 3 to 24 weeks | No exercise, upper limb strengthening exercise | / |
| (181) | Jan 2017 | 12/1,080 | Chronic non-specific low back pain | Females: range 45 to 83% | Range: 34 to 48 | Yoga: Most trials used Iyengar, Hatha, or Viniyoga forms of yoga & included meditation, relaxation, or breathing exercises in addition to physical yoga poses; duration: 1 to 7 per week, 45 to 120 minutes per session | Usual care, exercise or and no exercise | Supplementary written  advice or educational material |
| (182) | Oct 2018 | 11/835 | People living with and beyond cancer | Females: 68% | Range: 46 to 73 | Exercise: strength training alone, strength training combined with cardiovascular exercise, combination of strength and mobility training, aerobic walking, unspecified physical exercise training and sensorimotor exercises, balance training; duration: 2 per day to 2 per week; 20 to 90 minutes per session4 weeks to 24 months | Usual care | Partial supervision |
| (183) | July 2018 | 7/841 | Hand rheumatoid arthritis | Females: 78% | Range: 20 to 94 | Hand exercise involving a combination of different exercise types (e.g. strengthening, stretching, and dexterity); duration: 7-3 per week; 3 weeks to 48 months | No exercise (joint protection education and advice) | Partly supervised |
| (184) | Sept 2018 | 2/40 | Implantable ventricular assist device | Females: 25% | Mean: 52 (range 40 to 60) | Exercise-based cardiac rehabilitation (aerobic using treadmill (and cycle ergometer) or resistance training or both); duration: 3 per week; 6 to 8 weeks | Usual care | / |
| (185) | July 2015 | 10/510 | Low back pain | Females: 0 to 100% | Mean: 38 (Range 22 to 50) | Pilates; duration: 10 to 90 days; 1 to 7 per week; 15 to 60 minutes per session) | Minimal intervention or no intervention | Partially supervised |
| (186) | April 2016 | 15/1,048 | Asthma (mild to moderate) | Females: 0 to 100% | Range: 25 to 51 | Yoga: yoga breathing alone, or yoga interventions that included breathing, posture, and meditation; duration: 2 weeks to 54 months; 15 to 180 minutes; 2 per day to 2 per week | sham yoga, usual care | Partly supervised, partly part of a multicomponent program including stress management and lifestyle modification |
| (187) | April 2015 | 12/754 | Older people without known cognitive impairment | Females: 40 to 100% | Range: 60 to 91 | Aerobic exercise; duration: 8 to 26 weeks; 30 to 90 minutes per session; 2 to 6 per week | Active intervention such as stretching and toning; no intervention | Partially supervised |
| (188) | Nov 2011 | 11/598 | Chronic obstructive pulmonary disease (COPD) | Females: 0 to 85% | Range: 52 to 72 | Leg exercise training (interval or high intensity training): cycle training & treadmill-walking exercise; duration: 27 to 72 minutes per session; 2 to 5 times per week; 3 to 16 weeks | Continuous training; low intensity training (both cycle training plus treadmill-walking exercise) | Breathing and relaxation exercises, resistance training, supervision |
| **Total** | / | 2,888/  485,110 | / | / | / | / | / | / |

**Table 1 Footnote:**

ACSM: American College of Sports Medicine, CVI: chronic venous insufficiency, CVD: cardiovascular disease, GDM: gestational diabetes mellitus, HR: heart rate, OA: osteoarthritis, RA: rheumatoid arthritis, RaR: rate ratio, RR: risk ratio, ROM: range of motion, VO2max: maximal oxygen uptake

- Not applicable

/ Not reported

a- Ninety-five females did resistance training; b- Considerable diversity in the frequency, intensity, and duration of interventions.

*Number of RCTs/total N = RCTs also denotes qRCTs, cluster etc. as per the authors’ inclusion criteria.

**Supplementary Table 2. Additional information from Cochrane systematic reviews of the effects of physical activity/exercise on health outcomes (n=150)**

| **Reference** | **ROB in RCTs** | **Meta-analyses (y/n)** | **Type of health outcomes** | **Effects (estimates)/overall result** | **Comment** |
| --- | --- | --- | --- | --- | --- |
| (39) | High | n | 1. Symptom scores on St. George’s Respiratory Questionnaire  2. FVC (forced vital capacity) and  FEV1 (forced expiratory volume in second)  4. Change in BMI or weight | 1. −10 units, 95% CI -18 to−1, p = 0.02, N=1  2-3. Weight loss was associated with some improvement in FEV1 and FVC (N=1)  4. Significant weight loss in the treatment group compared to controls (N=1) | Too heterogeneous data |
| (40) | High | y | 1. Body mass index (BMI)  2. Body weight | 1. MD -1.18 kg/m2, 95% CI -1.67 to -0.69 or -0.13 units, 95% CI -0.21 to -0.05  2. MD -3.67 kg, 95% CI -5.21 to 2.13 | The studies contributing most information to the outcomes had a low risk of bias |
| (41) | High | y | 1. Change in body weight (kg)  2. Change in percentage of body fat (%)  3. Change in fat-free mass (kg)  4. Change in cardiorespiratory fitness (VO2 max, mL/kg/minute) | 1. MD -0.10 kg, 95% CI 1.90 to 1.71; MD -1.70 kg, 95% CI -2.08 to -1.32 (Females who took part in a diet), MD -1.93 kg; 95% CI -2.96 to -0.89 (diet plus exercise programme); MD 0.30 kg, 95% CI -0.06 to 0.66 (diet alone and diet plus exercise group)  2. MD -2.19% body fat, 95% CI 3.52 to -0.86  3. –  4. MD 3.76 mL/kg/minute, 95% CI 1.46 to 6.07 | The small sample sizes, the small number of studies, and heterogeneity of the interventions |
| (42) | Low or unclear | y | 1. VO2 max  2. Health- related quality of  life | 1. MD 2.49 mL/kg/min, 95% CI 1.63 to 3.36  2. Difference in 18 of 21 domains reported | Random sequence generation, allocation concealment or blinding of outcome assessors were poorly described in ≥50% of included studies |
| (43) | High | y | 1. Total mortality  2. Cardiovascular mortality  3. Fatal and/or non-fatal myocardial infraction  4. Coronary artery bypass graft  5. Percutaneous coronary intervention  6. Hospital admissions | 1. RR 0.96, 95% CI 0.88 to 1.04  2. RR 0.74, 95% CI 0.64 to 0.86  3. RR 0.90, 95% CI 0.79 to 1.04  4. RR 0.96, 95% CI 0.80 to 1.16  5. RR 0.85, 95% CI 0.70 to 1.04  6. RR 0.82 95% CI 0.70 to 0.96 | The quality of the evidence ranged from low to moderate |
| (44) | High | y | 1. Work performance  2. VO2 max  3. Peak heart rate | 1. WMD 4.26, 95%CI 2.06, 6.45  2. MD 0.30, 95%CI 3.17, 3.77  3. MD 2.84, 95% CI 5.05, 10.73 | The quality of the evidence was not evaluated using GRADE criteria |
| (45) | High | n | 1. Ejection fraction  2. Half venous refilling time  3. Total venous refilling time | 1. MD 4.88%, 95% CI 3.16 to 6.60  2. MD 4.20 seconds, 95% CI 3.28 to 5.12  3. MD 9.40 seconds, 95% CI 7.77 to 11.03 | The overall quality of evidence was very low |
| (46) | Low-high | n | Measures of functional activity | After 6 months, most studies showed that exercise programmes, improve physical function, quality of life, blood cholesterol levels, walking speed and leg pain after walking | Studies were too heterogeneous for pooling; small number of studies and participants |
| (47) | High | y | 1. Pain  2. Disability  3. Quality of life | 1. SMD −0.31, 95% CI −0.47 to −0.15  2. SMD −0.32, 95% CI −0.47 to −0.17  3. SMD −0.25 95% CI −0.49 to −0.01 | The evidence was downgraded due to high risk of bias |
| (48) | High | y | 1. Quality of life  2. Asthma symptoms  3. Exacerbations  4. Urgent physician visits  5. FEV1  6. VO2 max | 1. MD 0.26, 95% CI -1.05 to 1.58  2. MD 0.06, 95% CI -0.58 to 0.47  3. –  4. MD 0.08, 95% CI -0.25 to 0.42  5. MD 0.10, 95% CI 0.00 to 0.20  6. MD 9.67, 95% CI 5.84, 13.51 | The quality of the evidence ranged from low to high |
| (49) | Unclear | y | 1. Overall survival  2. Quality of life  3. Subscales physical functioning  4. Depression  5. Subscale anxiety  6. Fatigue  7. Severity of adverse events  8. Adverse events | 1. RR 0.93, 95% CI 0.59 to 1.47 2. SMD 0.26, 95% CI 0.03 to 0.49  3. SMD 0.33, 95% CI 0.13 to 0.52  4. SMD 0.25, 95% CI -0.00 to 0.50  5. SMD -0.18, 95% CI -0.64 to 0.28  6. SMD 0.24; 95% CI 0.08 to 0.40  7. RR 1.44, 95% CI 0.96 to 2.18  8. RR 7.23; 95% CI 0.38 to 137.05 | There was a tendency to over-emphasise the findings based on statistical significance |
| (50) | High | y | 1. Health-related quality of life  2. Pain intensity  3. Fatigue  4. Stiffness  5. Physical function  6. Withdrawals  7. Adverse events | 1. MD -7.89, 95% CI -13.23 to -2.55  2. MD -11.06, 95% CI -18.34 to -3.77  3. MD -6.06, 95% CI -12.41 to 0.30  4. MD -7.96, 95% CI -14.95 to -0.97  5. MD -10.16, 95% CI -15.39 to -4.94  6. RR 1.25, 95% CI 0.89 to 1.77  7.- | Absolute change ranged from 5% for withdrawals to 11% for pain intensity |
| (51) | High | y | 1. HRQL  2. Pain intensity  3. Fatigue  4. Stiffness | 1. MD -3.73 95% CI -10.81 to 3.35;  2. MD -28.22 95% CI -43.26 to -13.18  3. MD -33 95% CI -49 to -16  4. MD -26.27 95% CI -42.96 to -9.58 | Small number of studies with small samples; the quality of the evidence was very low |
| (52) | Low | y | 1. Multidimensional function  2. Pain  3. Stiffness  4. Muscle strength  5. Cardiovascular fitness (six-minute walk test) | 1. MD -5.97, 95% CI -9.06 to -2.88  2. MD -6.59, 95% CI -10.71 to -2.48  3. MD -18.34, 95% CI -35.75 to -0.93  4. SMD 0.63, 95% CI 0.20 to 1.05  5. MD 37.03 95% CI 4.14 to 69.92 | / |
| (53) | High | y | 1. Cardiorespiratory fitness  a. 9-minute run-walk  b. timed up-and-down stairs  2. Body composition  a. bone mineral density  b. body mass index  3. Flexibility (passive ankle dorsiflexion)  4. Muscle strength | 1a. SMD 0.69, 95% CI 0.02 to 1.35  1b. SMD -0.54, 95% CI -1.77 to 0.70  2a. SMD 1.07, 95% CI 0.48 to 1.66  2b. SMD 0.59, 95% CI -0.23 to 1.41  3. SMD 0.69, 95%CI 0.12 to 1.25  4. SMD 1.41, 95% CI 0.71 to 2.11 | The quality of the evidence was predominantly low or very low |
| (54) | High | y | 1. Depression  2. Stress  3. Fatigue  4. Body image  5. Anxiety | 1. SMD 0.02, 95% CI -0.28 to 0.32  2. SMD -0.18, 95% CI -0.48 to 0.12  3. SMD -0.36, 95%-1.26 to 0.55  4. SMD -0.13, 95% CI -0.61 to 0.34  5. SMD 0.21, 95% CI -0.09 to 0.51 | The quality of the evidence was very low for all outcomes |
| (55) | High | y | 1. Mental state  2. Social functioning  3. Quality of life (mental health)  4. Physical health  5. Leaving the study early | 1. RR 0.81 95% CI 0.62 to 1.07  2. RR 0.90 95% CI 0.78 to 1.04  3. MD -5.30 95% CI -17.78 to 7.18  4. MD 9.22 95% CI -0.42 to 18.86  5. RR 0.64 95% CI 0.49 to 0.83 | The quality of the evidence was predominantly low |
| (56) | High | y | 1. Mental state  2. Social functioning  3. Quality of life (mental health)  4. Quality of life (physical health)  5. Leaving the study early | 1. RR 0.70, 95% CI 0.55 to 0.88  2. RR 0.88, 95% CI 0.77 to 1  3. MD 15.50, 95% CI 4.27 to 26.73  4. MD 6.60, 95% CI -2.44 to 15.64  5. RR 0.91, 95% CI 0.6 to 1.37 | The quality of the evidence was predominantly moderate |
| (57) | High | y | 1. Quality of life  2. Leaving the study early | 1. MD 22.93 95%CI 19.74 to 26.12  2. RD 0.06, 95%CI -0.01 to 0.13 | Clinically important data were not available |
| (58) | Unclear | y | 1. Preeclampsia  2. Caesarean section  3. Postnatal weight  4. Induction of labour | 1. RR 0.31, 95% CI 0.01 to 7.09  2. RR 0.86, 95% CI 0.63 to 1.16  3. MD 0.11, 95% CI -1.04 to 1.26  4. RR 1.38, 95% CI 0.71 to 2.68 | Development of type 2 diabetes, perineal trauma and postnatal depression were not reported in the included studies |
| (59) | / | y | 1. Global well-being  2. Physical function  3. Pain  4. Tender points | 1. SMD 0.49, 95% CI 0.23 to 0.75  2. SMD 0.66, 95% CI 0.41 to 0.92  3. SMD 0.65, 95% CI: -0.09 to 1.39  4. SMD 0.23, 95% CI: -0.18 to 0.65 | The mean quality scores for internal validity were 5.06- moderate quality |
| (60) | High | y | 1. Multidimensional function  2. Self-reported physical function  3. Pain  4. Tenderness  5. Muscle strength  6. Attrition rates  7. Adverse effects | 1. MD -16.75, 95% CI -23.31, -10.19  2. MD -6.29, 95% CI -10.45 to -2.13  3. MD -3.3, 95% CI -6.35 to -0.26  4. MD -1.84, 95% CI -2.6 to -1.08  5. MD 27.32, 95% CI 18.28 to 36.36  6. RR 3.50, 95% CI 0.79 to 15.49  7. - | Incomplete description of the exercise protocols, inadequate small sample sizes, and inadequate documentation of adherence to exercise prescriptions |
| (61) | High | y | 1. Rate of falls  2. Risk of falling  3. Risk of fracture  4. Adverse effects | 1. RaR 0.93, 95% CI 0.72 to 1.20  2. RR 1.02, 95% CI 0.88 to 1.18  3. RR 0.88, 95% CI 0.25 to 3.14  4. - | The quality of the evidence was predominantly very low |
| (62) | High | y | 1. Asthma symptoms  2. Quality of life  3. Exercise tolerance  4. Peak expiratory flow rate  5. Minute ventilation at maximal exercise  6. VO2 max  7. HR max | 1. Qualitative synthesis  2. Qualitative synthesis  3. Qualitative synthesis  4. Qualitative synthesis  5. MD 3.08, 95% CI -0.63 to 6.79  6. MD 4.92, 95% CI 3.98 to 5.87  7. MD 3.67, 95% CI 0.90 to 6.44 | The quality of the evidence was predominantly very low or low |
| (63) | High | y | 1. Shoulder pain  2. Shoulder disability  3. Shoulder Pain and Disability Index  4. Quality of life | 1. MD -6.26, 95% CI -12.20 to -0.31  2. MD - 8.48, 95% CI -15.07 to -1.88  3. MD 5.77, 95% CI -14.00 to 2.46  4. MD 5.05, 95% CI -3.01 to 13.12 | Very small number of patients and primary studies |
| (64) | High | y | 1. Risk of postoperative pulmonary complication  2. Number of days patients needed intercostal catheter  3. Postoperative length of hospital stay  4. Post-intervention capacity  5. Forced vital capacity | 1. RR 0.33, 95% CI 0.17 to 0.61  2. MD -3.33, 95% CI -5.35 to -1.30  3. MD -4.24, 95% CI -5.43 to -3.06  4. MD 18.23, 95% CI 8.50 to 27.96  5. MD 2.97%, 95% CI 1.78 to 4.16 | The quality of the evidence was predominantly low |
| (65) | High | y | 1.Exercise capacity  2. Health-related quality  of life  3. Lung function | 1. MD 50.35, 95% CI 15.45, 85.24  2. SMD 0.17, 95%CI -0.16 to 0.49  3. MD -0.13, 95% CI -0.36 to 0.11 | The quality of the evidence was low for all outcomes |
| (66) | Unclear | y | 1.Perinatal outcomes  2.Pregnancy complications  3.Maternal morbidity | 1.-  2.-  3.- | Outcomes were not reported in any of the studies |
| (67) | High | y | 1. Recurrence of LBP  2. Number of recurrences  3. Time to LBP recurrence  4. Number of recurrences of LBP | 1. RR 0.50, 95% CI 0.34 to 0.73  2. MD -0.35, 95% CI -0.60 to -0.10  3. HR 0.43, 95% CI 0.21 to 0.87  4. MD -0.35, 95% CI -0.60 to 0.1 | The quality of the evidence was moderate for all outcomes under main comparison |
| (68) | High | y | 1. Changes in BMI z score  2. Adverse events  3. Health related quality of life  4. All- cause mortality  5. Morbidity  6. Parent- child relationship  7. Socioeconomic effects | 1. MD -0.4, 95% CI -0.6 to -0.2  2.-  3.-  4.-  5.- 6.-  7.- | Low quality evidence; poorly reported outcomes |
| (69) | High | n | 1. Functional exercise capacity  2. Health-related quality of life  3. Withdrawals  4. Adherence  5. Mortality  6. Loss to follow-up  7. Adverse events | 1. MD 1.8, 95% CI 0.4 to 3.2 (1 study)  2. No difference  3. No difference  4. See table 5  5. No difference  6. No difference  7. No difference | Individual study findings were inconsistent and poorly reported (often by 3 levels) |
| (70) | High | y | 1. Symptoms of depression  2. Symptoms of depression (long-term)  3. Adverse events  4. Acceptability of treatment  5. Quality of life (physical domain)  6. Quality of life (other domains) | 1. SMD -0.62, 95%CI: -0.81 to -0.42  2. SMD -0.33, 95%CI: -0.63 to -0.03  3. See table 4.  4. RR 1.00, 95% CI 0.97 to 1.04  5. SMD 0.45, 95% CI 0.06 to 0.83  6. No difference | The effect size was ’moderate’ for 1. and small for 2. (using Cohen’s rule of thumb); mainly moderate certainty evidence |
| (71) | Low-unclear | y | 1. Disability  2. Arm motor function  3. Arm Motor Impairment  4. Quality of life  5. Dexterity | 1. SMD 0.24, 95% CI -0.05 to 0.52  2. SMD 0.34, 95% CI 0.12 to 0.55  3. SMD 0.82, 95% CI 0.31 to 1.34  4. MD 6.54, 95%CI -1.2 to 14.28  5. SMD 0.42, 95% CI 0.04 to 0.79 | The quality of the evidence was low for disability and very low for the ability to use the affected arm |
| (72) | Low-unclear | y | 1. Health-related quality of life  2. Depression  3. Anxiety  4. Fatigue  5. Sleep disturbances | 1. SMD 0.22, 95% CI 0.04 to 0.40  2. SMD -0.13, 95% CI -0.31 to 0.05  3. SMD -0.53, 95% CI -1.10 to 0.04  4. SMD -0.48, 95% CI -0.75 to -0.20  5. SMD -0.25, 95% CI -0.40 to -0.09 | The quality of the evidence ranged from very low to moderate |
| (73) | High | y | 1.Fatigue (all data)  2.Quality of life  3.Anxiety  4.Depression  5.Self-efficacy | 1. SMD -0.27, 95% CI -0.37 to -0.17  2.No difference  3.Qualitative synthesis  4.Qualitative synthesis  5. No difference | Missing data for 32% of the studies |
| (74) | Low (n=1) and high (n=1) | y | 1. Functional Rating Scale  2. Quality of life  3. Fatigue Severity Scale  4. Manual muscle strength  5. Adverse effects | 1. MD 3.21, 95% CI 0.46 to 5.96  2. MD 2.70, 95% CI -3.10 to 8.50  3. MD -6.25, 95% CI -13.82 to 1.31  4. MD -10.90 95% CI -23.56 to 1.76  5.- | The quality of the evidence was predominantly moderate |
| (75) | Low | y | 1. Exercise capacity (walking test)  2. Maximal exercise capacity  3. Health-related quality of life | 1. MD 53.8, 95% CI 34.36 to 73.26  2. MD 9.54, 95% CI -0.38 to 19.47  3. MD 2.58, 95% CI 0.72 to 4.44 | The quality of the evidence was very low for all outcomes |
| (76) | Unclear to high | y | 1. Change in hot flushes | 1. SMD -0.10, 95% CI -0.33 to 0.13 | Three studies, 454 participants, low quality evidence |
| (77) |  | y | 1. Functional status  2. Mortality  3. ICU admission  4. Length of hospital stay | 1. SMD, 0.17, 95% CI -0.06 to 0.40  2. RR 1.98, 95% CI 0.64 to 6.18  3. RR 1.06 95% CI 0.04 to 30.44  4.WMD 0.01, 95%CI -1.23 to 1.26 | Study quality range: 4 to 8; mean: 6/10 |
| (78) | Moderate | y | 1. Television viewing  2. Physical activity rates  3. Physical activity duration  4. Blood pressure  5. Body mass index | 1. Range: 85-285 vs 89-288  2. Range: 53-92 vs 44-91  3. Range: 3-158 vs 3-143  4. Range: 96-138 / 50-73 vs 97-139 / 50-76  5. Range: 11.3-26 vs 12-26.1 | Results synthesized qualitatively; lack of blinding, consistency in measuring outcomes |
| (79) | High | n | 1. Physical function  2. Physical performance  3. Adverse events | 1. RR 1.71, 95% CI 1.11 to 2.64  2. MD 6.10; 95%CI -11.85 to 24. 05  3. See table 4. | Summary of findings for the main comparison based on one trial (expect for adverse events); low certainty of the evidence for all outcomes |
| (80) | High | y | 1. Children’s self-esteem | 1. SMD 0.49, 95% CI 0.16 to 0.81 | Eight trials were pooled with 380 subjects; effect size was even larger for studies at low risk of bias |
| (81) | Low | y | 1. Subjective impact of fatigue on ADL, fatigue severity  2. Health-related quality of life | 1. SMD -0.45, 95% CI -1.21 to 0.32  2. SMD -0.08, 95% CI -0.60, 0.45 | Based on two trials (n=57); and low certainty of the evidence |
| (82) | High | y | 1. Distress  2. Fatigue  3. Anxiety  4. Depression  5. Quality of sleep  6. Adverse events | 1. MD -0.30, 95% CI -5.55 to 4.95  2. MD 0.00, 95% CI -0.94 to 0.94  3., MD 0.30, 95% CI -5.01 to 5.61  4. MD -0.70, 95% CI -3.21 to 1.81  5. MD -2.30, 95% CI -3.78 to -0.82  6. - | Very low certainty of the evidence; data from one study with very small sample |
| (83) | High | y | 1. Cognition  2. ADLs  3. Depression  4. Neuropsychiatric symptoms | 1. SMD 0.43, 95% CI -0.05 to 0.92  2. SMD 0.68, 95% CI 0.08 to 1.27  3. SMD 0.14, 95% CI -0.07 to 0.36  4. MD -0.60, 95% CI -4.22 to 3.02 | Effect sizes ranged from small to moderate |
| (84) | Low | y | 1. Pain  2. Physical function  3. Quality of life  4. Study withdrawals or dropouts | 1. SMD -0.49, 95% CI -0.39 to -0.59  2. SMD -0.52, 95% CI 0.39 to -0.64  3. SMD 0.28, 95% CI 0.15 to 0.40  4. see Table 5 | Predominantly high quality evidence |
| (85) | Low | y | 1. Pain  2. Physical function  3. Quality of life  4. Study withdrawals or dropouts  5. Adverse effects | 1. SMD 0.38, 95% CI -0.55 to -0.20  2. SMD -0.38, 95% CI -0.54 to -0.05  3. SMD -0.07, 95% CI -0.23 to 0.36  4. see Table 5  5. - | Low to high quality evidence |
| (86) | Unclear or high | y | 1. Change in Asthma Quality of Life Questionnaire  2. Change in St George’s Respiratory Questionnaire  3. Asthma symptoms  4. Number of acute exacerbations  5. Inpatient hospitalisation  6. Lung function (FEV1)  7. Days off work | 1. MD 0.79; 95%CI 0.50 to 1.08  2. MD -5.9; 95%CI -12.64, 0.84  3. MD -3.22; 95%CI -6.31 to -0. 13  4. Between group difference (p < 0.005)  5. –  6. No between group difference (p= 0.07)  7. - | The quality of the evidence was very low for all reported outcomes |
| (87) | High | y | 1. Physical fitness  2. Fatigue  3. Cancer- specific QOL  4. Health-related QOL  5. Depression  6. Cognitive function  7. Lymphoedema | 1. SMD 0.42, 95% CI 0.25 to 0.59  2. SMD -0.28, 95% CI -0.41 to -0.16  3. SMD 0.12 95%CI 0.00 to 0.25  4. MD 1.10, 95% CI -5.28 to 7.48  5. SMD -0.15, 95% CI -0.30 to 0.01  6. MD -11.55, 95% CI -22.06 to -1.05  7. RR 0.71 95%CI 0.35 to 1.45 | Very serious risk of bias in all included studies |
| (88) | High | y | 1. Fractures  2. Adverse events  3. Falls  4. Pain  5. Physical Function: Mobility  6. QOL | 1. -  2. Please see table 4  3.-  4. Qualitative synthesis  5. MD -1.13, 95% CI -1.85 to -0.42  6. Qualitative synthesis | Very low quality of the evidence was for all reported outcomes |
| (89) | Low to high | y | 1. Rate of falls (multiple-component exercise)  2. Risk of falling (as above)  3. Rate of falls (for Tai Chi)  4. Risk of falling (as above) | 1. RaR 0.71, 95% CI 0.63 to 0.82  2. RR 0.85, 95% CI 0.76 to 0.96  3. RaR 0.72, 95% CI 0.52 to 1.00  4. RR 0.71, 95% CI 0.57 to 0.87 | Some trials failed to adhere to CONSORT |
| (90) | High | n | 1. Mental state (Mental Health Inventory Depression)  2. Mental state (Positive and Negative Syndrome Scale)  3. Physical health  4. Physical quality of life scores | 1. MD 17.50 CI 6.70 to 28.30  2. MD -8.50 CI -11.11 to -5.89  3. MD 79.50 CI 33.82 to 125.18  4. MD -9.22 CI -18.86 to 0.42 | Data from a single study (not pooled) |
| (91) | High | y | 1. Number of acute respiratory infections per year  2. Proportion of participants who experienced at least one acute respiratory infection over the study period  3. Global severity over 8 weeks  4. Number of symptom days in 12 weeks follow-up  5. Number of symptom days per episode of illness over 12 weeks | 1. RR 0.91; 95% CI 0.59 to 1.42  2. RR 0.76, 95% CI 0.57 to 1.01  3. MD -110, 95% CI -324 to 104  4. MD -2.1, 95% CI -4.4 to 0.3  5. MD -1.1, 95% CI -1.7 to -0.5 | The quality of the evidence ranged from low to moderate (predominantly low) |
| (92) | High | y | 1. Incidence of influenza  2. Complications  3. Adverse effects of vaccination  4. Numbers of working days or days lost | 1. RR 0.22, CI 0.01 to 4.40  2. –  3. –  4. – | 1.Very low certainty  2-4: no data |
| (93) | High | n | 1. QOL  2. Exacerbations  3. Lung function  4. Medication usage  5. Adverse events | 1. -  2. RR 1.65, 95% CI 0.17 to 16.33  3. qualitative synthesis  4.-  5. - | Very low certainty evidence; poor reporting |
| (94) | High | y | 1. Pain  2. Function  3. QOL  4. Patient Satisfaction  5. Global Perceived Effect  6. Adverse Effects | 1. MD -0.67, 95% CI -1.32 to -0.02  2. MD -2.80, 95% CI-6.36 to 0.76  3. SMD -0.18, 95% CI -0.48 to 0.13  4. SMD -0.93, 95% CI -1.35 to -0.52  5. SMD -0.42, 95% CI -0.81 to -0.03  6. See table 4 | Predominantly moderate certainty evidence |
| (95) | High | y | 1. Maximal treadmill walking distance  2. Pain-free treadmill walking distance  3. Quality of life (physical)  4. Self-reported functional impairment | 1. SMD 0.37, 95% CI 0.12 to 0.62  2. SMD 0.51, 95% CI 0.21 to 0.81  3. MD 0.0, 95% CI -4.79 to 4.79  4. MD -5.0, 95%CI -19.19 to 9.19 | The quality of the evidence ranged from very low to moderate |
| (96) | High | y | 1. Functional assessment  2. Time to complete a 50-foot walking test  3. Joint tenderness  4. Swollen joints  5. Ankle plantar flexion  6. Lower extremity flexion  7. Shoulder flexion  8. Shoulder internal and external rotation  9. Upper extremity ROM  10. Grip strength | 1. WMD 0.01, 95% CI -2.94, 2.97  2. WMD 0.35, 95%CI, -1.14 to 1.84  3. WMD -0.83, 95%CI, -3.30 to 1.64  4. WMD 2.45, 95% CI, -0.45 to 5.36  5. WMD 24.00, 95% CI, 3.34 to 44.66  6. WMD: 34.00, 95% CI, 10.79 to 57.21  7. WMD 21.00, 95% CI, -17.56 to 59.56  8. WMD 42.00 degrees, 95%CI, -7.97 to 91.97)  9. WMD 56.00 degrees, 95%CI: -63.90 to 175.90 10. WMD -0.08, 95% CI, -0.26 to 0.10 | Controlled clinical trials were included |
| (97) | High | y | 1. Incidence of gestational diabetes  2. Caesarean section  3. Vaginal birth | 1. RR 1.10, 95% CI 0.66 to 1.84  2. RR 1.33, 95% CI 0.97 to 1.84  3. RR 0.83, 95% CI 0.58 to 1.17 | In two studies, baseline imbalances were noted in maternal education level, parity, exercise habits before gestation |
| (98) | High | y | 1. Change in blood pressure  2. Change in LDL cholesterol  3. Change in HDL cholesterol  4. Change in triglycerides | 1. MD -2.90, 95%CI -4.52 to -1.28  2. MD -0.09, 95%CI -0.48 to 0.30  3. MD 0.08, 95%CI 0.02 to 0.14  4. MD -0.27, 95%CI -0.44 to -0.11 | Cardiovascular mortality, all-cause mortality were not reported |
| (99) | Unclear | n | 1. Systolic blood pressure  2. Diastolic blood pressure  3. Total cholesterol  4. LDL cholesterol  5. HDL cholesterol  6. Triglycerides | 1. Qualitative synthesis  2. Qualitative synthesis  3. Qualitative synthesis  4. Qualitative synthesis  5. Qualitative synthesis  6. Qualitative synthesis | Effect sizes ranged considerably: 5.20 to -22.0 for e.g., systolic blood pressure |
| (100) | Mainly unclear | n | 1. Mortality  2. Stroke mortality  3. Stroke incidence  4. Change in blood pressure  5. Change in total cholesterol  6. Change in LDL-C cholesterol  7. Change in HDL-C cholesterol  8. Change in triglycerides | 1. Qualitative synthesis  2. Qualitative synthesis  3. RR 0.56; 95% CI 0.38 to 0.83  4. Qualitative synthesis  5. Qualitative synthesis  6. Qualitative synthesis  7. Qualitative synthesis  8. Qualitative synthesis | Considerable clinical and statistical heterogeneity |
| (101) | High | y | 1. Cardiorespiratory fitness  2. Depression  3. Cognition  4. Fatigue  5. Community integration  6. Safety | 1. MD 35.47, 95% CI 2.53 to 68.41  2. SMD -0.43, 95% CI -0.92 to 0.06  3. -  4. SMD -0.32, 95% CI -0.90 to 0.26  5. -  6. - | Predominantly very low certainty evidence |
| (102) | High | y | 1. Pain intensity  2. Function | 1. MD -7.29, 95% CI -10.91to -3.67  2. MD -4.31, 95% CI -7.41 to -1.20 | Only 8 (13.1%) trials had high internal validity |
| (103) | Mainly unclear | y | 1. Patients’ perception of change 2. Condition-specific QOL | 1. RR 0.89, 95% CI 0.78 to 1.03 (not cured)  2. - | There were 27 different comparisons and analyses |
| (104) | High | y | 1. Fatigue | 1. SMD -0.35, 95% CI -0.57 to -0.13 | Moderate certainty evidence |
| (105) | High | y | 1. Physical fitness  a. aerobic capacity  b. walking capacity  2. Cardiovascular dimensions  a. resting diastolic blood pressure  b. resting systolic blood pressure  3. Heart rate  4. Nutritional parameters  a. albumin  b. pre-albumin  c. energy intake | 1a. SMD -0.56, 95% CI -0.70 to -0.42  1b. SMD -0.36, 95% CI-0.65 to -0.06  2a. MD 2.32, 95% CI 0.59 to 4.05  2b. MD 6.08, 95% CI 2.15 to 10.12  3. MD 6, 95% CI 10 to 2  4a. MD -2.28, 95% CI -4.25 to -0.32  4b. MD - 44.02, 95% CI -71.52 to -16.53  4c. SMD -0.47, 95% CI -0.88 to -0.05 | 49% of the trials has a high risk of bias |
| (106) | Low | y | 1. All-cause mortality  2. Incidence of type 2 diabetes  3. Serious adverse events  4. Cardiovascular mortality  5. Non- fatal myocardial infarction/stroke  6. Health- related quality of life  7. Socioeconomic effects | 1. RR 1.12, 95% CI 0.50 to 2.50  2. RR 0.57, 95% CI 0.50 to 0.64  3. See table 4  4.RR 0.94 (0.24 to 3.65)  5.-  6.-  7.- | The quality of the evidence ranged from (predominantly) very low to moderate |
| (107) | High | y | 1. Muscle soreness (day one)  2. Muscle soreness (day two) | 1.MD -0.52, 95% CI -11.30 to 10.26  2.MD 0.72, 95% CI -11.20 to 12.64 | Low certainty evidence |
| (108) | High | y | 1.Pain  2. Functional status  3. Return to work | 1.MD -0.33, 95% CI -1.29 to 0.64  2.MD 0.17, 95% CI -0.36, 0.71  3. - | I^2^ =66%; poor reporting under 3. |
| (109) | High | y | 1. Dyspnoea during exercise  2. Dyspnoea during daily life  3. Walking capacity  4.Health- related quality of life | 1. MD -1.00, 95% CI -2.10 to 0.10  2. MD -10, 95% CI -28.99 to 8.89  3. MD 10.10, 95% CI 37.21 to 62.99  4. MD -12.94, 95% CI -22.29 to -3.60 | Low certainty evidence for all outcomes |
| (110) | High | y | 1. Timed Up & Go test  2. Walking speed  3. Berg Balance Scale | 1. MD -0.82, 95% CI -1.56 to -0.08  2. SMD 0.43, 95% CI 0.11 to 0.75  3. MD 3.48, 95% CI 2.01 to 4.95 | Large review with >100 outcomes |
| (111) | High | y | 1. Total number of fractures  2. Bone mineral density % change  3. Adverse events: Falls | 1. OR 0.61, 95% CI 0.23 to 1.64  2. MD 0.85; 95% CI 0.62 to 1.07  3. See table 4 | High certainty evidence (chosen outcome with the largest number of studies) |
| (112) | High | y | 1. Functional ability  2. Muscle strength  3. Self-reported pain  4. Disease activity  5. Radiological damage | 1. SMD -0.54, 95% CI -1.11, 0.02  2. SMD: 0.47 (0.01 to 0.93)  3. SMD: -0.53 (- 1.09 to 0.04)  4. Qualitative synthesis  5. - | Moderate certainty evidence |
| (113) | High | y | 1. Pain  2. Physical function  3. Self- efficacy  4. Depression  5. Anxiety  6. Quality of life (social function)  7. Adverse effects | 1. SMD -0.33; 95% CI -0.46 to -0.21  2. SMD -0.27; 95%CI -0.37 to -0.17  3. SMD 0.46; 95%CI 0.34 to 0.58  4. SMD -0.16; 95% CI-0.29 to -0.02  5. SMD -0.11; 95%CI -0.26 to 0.05  6. MD 58.30; 95%CI 34.58, 82.02  7.- | The quality of the evidence ranged from low to moderate (predominantly moderate) |
| (114) | Unclear | n | 1. Quality of life  2. Symptoms | 1. –  2. Not significant | No numerical data presented; a single RCT included at unclear risk of bias |
| (115) | High | y | 1. Postoperative atelectasis  2. Postoperative pneumonia  3. Mechanical ventilation > 48 hours  4. All-cause mortality  5. Adverse events  6. Duration of hospital stay | 1. RR 0.53, 95% CI 0.34 to 0.82  2. RR 0.45, 95% CI 0.26 to 0.77  3. RR 0.55, 95% CI 0.03 to 9.2  4. RR 0.4, 95% CIK 0.04 to 4.23  5. See table 4.  6. MD 1.33, 95% CI -2.53 to -0.13 | The quality of the evidence ranged from very low to moderate (predominantly low) |
| (116) | High | y | 1. Fear of falling postintervention  2. Fear of falling <6 months  3. Occurrence of at least 1 fall  4. Depressive symptoms  5. Anxiety:  6. Physical activity | 1. SMD 0.37, 95% CI 0.18 to 0.56  2. SMD 0.17, 95% CI -0.05 to 0.38  3. RR 0.85, 95% CI 0.74-0.98  4. SMD -0.08, 95% CI -0.28 to 0.13  5. No difference  6. MD 3.44, 95% CI -1.65 to 8.54 | Effect sizes ranged from small to moderate |
| (117) | High | y | 1. Preterm birth  2. Mean gestational age | 1. RR 1.82, 95% CI 0.35 to 9.57  2. MD 0.10, 95% CI -0.11 to 0.30 | Inconsistent results; small number of studies and participants |
| (118) | High | y | 1. Health related QoL  2. Emotional function/mental  3. Perceived physical function  4. Anxiety  5. Depression  6. Fatigue  7. Cardiorespiratory fitness | 1. SMD 0.39 (0.21 to 0.57)  2. SMD 0.21 (0.10 to 0.32)  3. SMD 0.33 (0.18 to 0.49)  4. SMD -0.57 (-0.95 to -0.19)  5. SMD -0.34 (-0.62 to -0.05)  6. SMD -0.32 (-0.47 to -0.18)  7. SMD 0.44 (0.30 to 0.58) | The quality of the evidence was predominantly moderate |
| (119) | Low or unclear | y | 1. Pain-free walking distance  2. Maximum walking distance  3. Ankle brachial index  4. Mortality  5. Amputation  6. Quality of Life (physical) | 1. MD 82.11, 95% CI 71.73 to 92.48  2. MD 120.36, 95% CI 50.79 to 189. 92  3. MD 0. 04, 95% CI 0.00 to 0.08  4. RR 0.92, 95% CI 0.39 to 2.17  5. RR 0.20, 95% CI 0.01 to 4.15  6. MD 2.15, 95% CI 1.26 to 3.04 | Predominantly high or moderate quality evidence |
| (120) | High | y | 1. Fatigue  2. Participants with serious adverse reaction  3. QOL (physical)  4. Depression  5. Sleep  6. Self-perceived changes in overall health  7. Drop-outs | 1. MD 2.82, 95% CI 4.07 to 1.57  2. RR 0.99, 95% CI 0.14 to 6.97  3. MD 13.10, 95% CI 1. 98 to 24.22  4. MD 1.63, 95% CI 3.50 to 0. 23  5. MD -1.49, 95%CI -2.95 to -0.02  6. RR 1.83, 95% CI 1.39 to 2.40  7. RR 1.63, 95% C 0.77 to 3.43 | Very low to moderate evidence |
| (121) | High | y | 1. Anxiety  2. Depression | 1. SMD -0.48, 95% CI -0.97 to 0.01  2. SMD -0.66, 95% CI -1.25 to -0.08 | All trials were of low quality and were highly heterogeneous |
| (122) | Low | y | 1.Maximum walking distance  2.Pain- free walking distance  3.Health related QOL | 1. MD 8.15, 95% CI -2.63 to 18.94  2. MD 6.42, 95% CI -1.52 to 14.36  3. MD 26.50, 95% CI 2.67, 50.33 | Skewed data and the very small sample sizes |
| (123) | High | y | 1. Quality of life Stroke Impact Scale  2. Quality of life  3. Balance  4. Gait  5. Depression  6. Anxiety:  7. Disability | 1.MD 2.00, 95% CI -17.70 to 21.70  2.MD 2.80, 95% CI -2.03 to 7.63  3.MD 2.38, 95% CI -1.41 to 6.17  4.MD 1.32, 95% CI -1.35 to 3.99  5.MD -2.10, 95% CI -4.70 to 0.50  6.MD -6.70, 95% CI -15.35 to 1.95  7.OR 2.08, 95% CI 0.50 to 8.60 | Very low-quality evidence for all outcomes |
| (124) | High | y | 1.Pain  2. Adverse effects  3. Range of motion | 1. RR 0.39, 95% CI 0.22 to 0.68  2. RR 2.30, 95% CI 1.49 to 3.56  3. MD 6.17, 95% CI 0.14 to 12.20 | Unclear risk of bias and small median sample size |
| (125) | High | y | 1. Physical ability  2. Gait speed  3. Getting out of a chair  4. Muscle strength  5. Pain | 1. SMD 0.14, 95% CI 0.05 to 0.22  2. MD 0.08, 95% CI 0.04 to 0.12  3. SMD -0.94, 95% CI -1.49 to -0.38  4. SMD 0.84, 95% CI 0.67 to 1.00  5. SMD -0.30, 95% CI -0.48 to -0.13 | Poor methodological quality and low internal validity |
| (126) | High | y | 1. All-cause mortality  2. Acute myocardial infarction  3. Exercise capacity  4. Cardiovascular-related hospital admissions  5. Health-related QOL  6. Adverse events | 1.RR 1.01,95% CI 0.18 to 5.67  2.RR 0.33, 95% CI 0.07 to 1.63  3.SMD 0.45, 95% CI 0.20 to 0.70  4.RR 0.14, 95% CI 0.02 to 1.1  5.-  6.- | Only very low to low quality of evidence |
| (127) | High | y | 1.Physical fitness:  2. Safety  3. Health related QOL  4. Fatigue | 1. MD 0.05, 95% CI -0.03 to 0.13  2. See table 4  3. MD 2.29, 95% CI -1.06 to 5.65  4. MD - 1.05, 95% CI -1.83 to -0.28 | Low to moderate quality evidence |
| (128) | High | y | 1. Pain  2. Disability  3. Adverse events | 1. MD 6.00 [-4.76 to 16.70]  2. MD 1.30 [-5.90 to 8.50];  3. - | Low quality evidence; very small number of studies and participants |
| (129) | Unclear or high | y | 1. Quality of life  2. Asthma symptoms  3. Serious adverse events | 1. No between-group comparisons  2. No between-group comparisons  3. See Table 4. | Very small number of studies and overall sample size |
| (130) | High | y | 1. School achievement Mathematics  a. Reading  2. Composite executive functions  a. Inhibition control  3. Adverse events | 1. SMD 0.49, 95% CI -0.04 to 1.01  a. 0.10, 95% CI -0.30 to 0.49  2. MD 5.0, 95% CI 0.68 to 9.32  b. MD -1.55, 95% CI -5.85 to 2.75  3. See Table 4. | High to very low-quality evidence |
| (131) | High | y | 1. Symptoms of dyspnea  2. Health-related quality of life  3. Peak upper limb exercise capacity (supported)  4. peak upper limb exercise (unsupported)  5. Endurance upper limb exercise capacity (supported)  6. endurance upper limb exercise capacity (unsupported)  7. Upper limb strength | 1. MD 0.37, 95% CI 0.02 to 0.72  2. SMD 0.05, 95% CI −0.31 to 0.40  3. SMD 0.17, 95% CI −0.43 to 0.77  4. MD 21.23, 95% CI −20.45 to 62.92  5. SMD 0.25, 95% CI −0.46 to 0.96  6. SMD 0.66, 95% CI 0.19 to 1.13  7. SMD 0.01, 95% CI -0.70 to 0.73 | Low to moderate quality evidence (predominantly low) |
| (132) | High | y | 1. Exercise capacity - functional  2. Exercise capacity - peak  3. Exercise capacity - endurance  4. QOL | 1. MD 62, 95% CI 44 to 80  2. MD 50, 95% CI 20 to 80  3. MD 371, 95% CI 121 to 621  4. SMD -0.97, 95% CI -0.37 to -1.57 | Low to high quality evidence |
| (133) | High | y | 1.Shoulder flexion ROM  2.Wound drainage volume  3.Wound drainage duration | 1. WMD, 10.6, 95% CI 4.51 to 16.6  2. SMD, 0.31; 95% CI 0.13 to 0.49  3. WMD, 1.15, 95% CI 0.65 to 1.65 | Estimates for early versus delayed exercise |
| (134) | High | y | 1. Changes in BMI  2. BMI z score  3. Change in weight  4. Adverse events  5. Change in health related QOL  6. Child- reported measures  7. All-cause mortality  8. Morbidity  9. Socioeconomic effects | 1. MD -0.53, 95% CI -0.82 to - 0.24  2. MD -0.06, 95% CI -0.10 to -0.02  3. MD-1.45, 95% CI -1.88 to -1.02  4. RR 0.57, 95% CI 0.17 to 1.93  5. SMD 0.13, 95% CI -0.06 to 0.32  6. SMD 0.15, 95% CI -0.34 to 0.64  7.-  8.-  9.- | Low to very low-quality evidence |
| (135) | High | y | 1. Depression  2. Drop-outs  3. Social and occupational functioning  4. Quality of life  5. Self esteem | 1. SMD -0.67 95% CI -1.40 to 0.05  2. OR 1.82, 95% CI 0.35 to 9.45  3. MD -6.80 95 % CI -11.44 to -2.16  4. MD 0.30 95% CI -0.60 to 1.20  5. MD 1.70 95% CI -2.36 to 5.76 | Low to very low-quality evidence |
| (136) | Low | y | 1.Pre-eclampsia  2. Gestational hypertension  3. Caesarean section  4. Preterm birth  5. Small-for-gestational age | 1.RR 0.31, 95% CI 0.01 to 7.09  2.RR 1.0, 95% CI 0.07 to 13.37  3. RR 0.93, 95% CI 0.22 to 3.88  4. RR 1.0, 95% CI 0.07 to 13.37  5. RR 3.0, 95% CI 0.14 to 64.26 | Two trials only were included |
| (137) | Unclear | y | 1. Activities of daily living  2. Muscle strength  3. Ability to walk  4. Postural control  5. Aerobic fitness | 1. MD 13.20, 95% CI 8.36 to 18.04  2. MD 1.01, 95% CI 0.19 to 1.83  3. MD 0.14, 95% CI -0.32 to 0.606  4. MD 3.05, 95% CI -3.41 to 9.52  5. MD 3.6, 95% CI -0.53 to 7.73 | Small number of trials; total sample size <100 |
| (138) | Unclear | y | 1. Walking speed  2. Walking capacity  3. Adverse events  4. Drop-outs | 1. MD 0.06, 95% CI - 0.01 to 0.13  2. MD 10.29 95% CI 0.15 to 20.43  3. RD 0.00, 95% CI -0.07 to 0.07  4. RD 0.01, 95% CI -0.13 to 0.15 | Inconclusive results |
| (139) | Low-unclear | y | 1.Drop-outs  2.Walking speed  3.Walking endurance | 1. RD 0.00, 95% CI -0.01 to 0.01  2. MD 0.06, 95% CI 0.03 to 0.09  3. MD 14.19, 95% CI 2.92 to 25.46 | Low to moderate certainty evidence |
| (140) | High | y | 1. Health related QOL  2. Overall QOL change score  3. Anxiety change  4. Emotional wellbeing  5.Fatigue change  6. Pain follow-up  7. Sexuality change  8. Sleep disturbance  9. Social functioning | 1. SMD 0.48; 95%CI 0.16 to 0.81  2. SMD 0.46; 95%CI 0.09 to 0.84  3. SMD -0.26; 95% CI -0.44 to -0.07  4. SMD 0.33; 95%CI 0.05 to 0.61  5. SMD -0.82; 95% CI -1. 50 to -0.14  6. SMD -0.29; 95% CI -0. 55 to -0.04  7. SMD 0.40; 95%CI 0.11 to 0.68  8. SMD -0.46; 95% CI -0. 72 to -0.20  9. SMD 0.45; 95%CI 0.02 to 0.87 | Very low to moderate certainty evidence |
| (141) | High | y | 1. Overall HR QoL change score  2. Overall HR QoL  3. Anxiety  4.Depression  5. Fatigue change score  6. Overall physical function change score | 1. SMD 0.47; 95%CI 0.16 to 0.79  2. SMD 0.33; 95% CI 0.12 to 0.55  3. SMD -0.46; 95% CI -0. 81 to -0.11  4. SMD -0.55; 95% CI -0. 87 to -0.22  5. SMD -0.73; 95% CI -1. 14 to -0.31  6. SMD 0.69; 95% CI 0.16 to 1.22 | Predominantly very low certainty evidence |
| (142) | Moderate | n | 1.Pittsburgh Sleep Quality Index  2. Sleep diary (duration) | 1. MD 3.4, 95% CI 1.9 to 5.4  2. Average 42 minutes (p=0.05) | Findings from one trial only |
| (143) | High | y | 1. Change in functional exercise capacity  2. Exercise capacity: mean peak oxygen uptake  3. Exercise capacity: mean peak power  4. HR QoL: physical  5. HR QoL: mental | 1. MD 60.12, 95% CI 30.17 to 90.07  2. MD 2.4, 95% CI 1.4 to 3.4  3. MD 16.4, 95% CI 10.9 to 22.0  4. MD 4.63, 95% CI 0.80 to 8.47  5. MD 4.17, 95%CI 0.01 to 8.34 | Low certainty evidence |
| (144) | Low or unclear | y | 1. Excessive weight gain  2.Low weight gain  3.Preterm birth  4.Pre-eclampsia  5.Caesarean delivery  6. Infant birthweight > 4000 g | 1. RR 0.80, 95% CI 0.73, 0.87  2. RR 1.14, 95% CI 1.02 to 1.27  3. RR 0.91, 95% CI 0.68 to 1.22  4. RR 0.95, 95% CI 0.77 to 1.16  5. RR 0.95, 95% CI 0.88 to 1.03  6. RR 0.93, 95% CI 0.86 to 1.02 | Predominantly high-quality evidence |
| (145) | High | y | 1.Dyspnea  2.Functional capacity  3.Aerobic capacity  4.Pulmonary function  5.Quality of life St. George’s Respiratory Questionnaire | 1. MD -0.20, 95% CI -0.67 to 0.27  2. MD 29.64, 95% CI 10.52 to 48.77  3. MD 3.2, 95% CI -5.76 to 1.76  4. MD 0.11 L, 95% CI 0.02 to 0.20  5. MD 7.85, 95%CI -16.53 to 0.83 | Predominantly low-quality evidence |
| (146) | Low | y | 1. Full continence | 1. RR 0.70, 95% CI 0.52 to 0.94 | Studies compared different combinations of treatments and different outcome measures |
| (147) | Unclear | y | 1. CD4 count  2. Mean body weight  3. Mean girth  4. Maximum heart rate | 1.MD 48.32, 95% CI -6.60 to 103.23  2.MD 3.54, 95%CI 2.21to 4.87  3.MD 7.91, 95%CI 2.18 to 13.65  4.MD -13.02, 95%CI -26.67 to 0. 64 | Participant blinding was not achieved in the trials |
| (148) | High | y | 1. CD4 count  2. Viral load  3. VO2 max  4. Maximum heart rate | 1. MD 18.08, 95%CI -11.82 to 47. 99  2. MD 0.40, 95%CI -0.28 to 1.07  3. MD 2.63, 95%CI 1.19 to 4.07  4. MD -9.81, 95%CI -26.28 to 6.67 | Hawthorne effect suspected |
| (149) | High | y | 1.Hand pain  2.Hand function  3. Quality of life  4. Finger joint stiffness  5. Adverse events  6. Withdrawals due to adverse events | 1. SMD -0.27, 95%CI -0.47 to -0. 07  2. SMD -0.28, 95%CI -0.58 to 0. 02  3. MD 0.30, 95%CI -3.72 to 4.32  4. SMD -0.36, 95%CI -0.58, -0.15  5. RR 4.55, 95%CI 0.53 to 39.31  6. RR 2.88, 95%CI 0.30 to 27.18 | Very low or low certainty evidence |
| (150) | High | y | 1. Overall pain  2. Function  3. Global treatment success  4. Adverse events | 1. MD -26, 95%CI -36.80 to -15.20  2. MD -25.00, 95% CI -35.24 to -14.76  3. RR 0.6, 95%CI 0.44 to 0.83  4. RR 1.07, 95%CI 0.76 to 1.49 | Moderate certainty evidence |
| (151) | High | n | 1. Overall pain  2. Function  3. Pain on motion  4. Global treatment success  5. QOL  6. Adverse events | 1. MD 6.8, 95% CI -0.70 to 14.30  2. MD 7.1, 95% CI 0.30 to 13.90  3. MD 0. 9, 95% CI-0.03 to 1.7  4. RR 1.39, 95% CI 0.94 to 2.03  5. MD 0.07, 95% CI 0.04 to 0.1  6. RR 3.77, 95% CI 1.49 to 9.54 | High quality evidence |
| (152) | High | n | 1. Short- term overall improvement  2. Adverse effects  3. Short term improvement pain  4. Short- term improvement in functional ability  5. Short- term improvement in neurophysiologic parameters  6. Long- term improvement in symptoms  7. Long- term improvement in functional ability  8. Need for surgery | 1.RR 15, 95% CI 1.02 to 220.92  2. See table 4.  3.MD -0.57, 95% CI -1.73 to 0.59  4. RR 9, 95% CI 0.59 to 137.65  5. -  6. RR 0.33, 95% CI 0.1 to 1.12  7.-  8. RR 0.33, 95% CI 0.1 to 1.12 | Very low certainty evidence |
| (153) | High | n | 1. Seizure free | 1. OR 14.54, 95% CI 0.67 to 316.69 | Low certainty evidence |
| (154) | High | n | 1. Swallowing function  2. Adverse event  3. Aspiration  4. Penetration | 1. MD -8.06, 95% CI -25.37 to 9.25  2. RR 0.62, 95% CI 0.22 to 1.71)  3. 18.18% versus 7.69%  4. 45.45% versus 23.08% | Very low certainty evidence |
| (155) | Unclear | y | 1. Exercise capacity  2. Pulmonary function  3. HRQoL  4. Pulmonary exacerbations  5. Adverse events | 1.Qualitative synthesis  2. Qualitative synthesis  3. Qualitative synthesis  4. Qualitative synthesis  5. See table 4. | Very low to moderate certainty evidence |
| (156) | High | y | 1. Pain  2.Function  3. Quality of life  4. Adverse events  5. Serious adverse events | 1.MD -0.84, 95% CI - 1.63 to -0.04  2. MD -2.65, 95% CI -5.29 to -0.01  3. MD 4.3, 95% CI -6.5 to 15.2  4. OR 1.72, 95% CI 0.51 to 5.81  5. See table 4. | Very low to low certainty evidence |
| (157) | High | y | 1. Clinical global response  2. Mental state  3. Satisfaction  4. Quality of life | 1. RR 0.44, 95%CI 0.04 to 4.49  2. MD -4.40, 95%CI -8.15 to -0.65  3. MD 0.40, 95%CI -0.78 to 1.58  4. MD 0.00 95% CI -0.48 to 0.48 | High drop-out rate; moderate certainty evidence |
| (158) | Low | n | 1. Activities limitation  2. Health-related QOL | 1.Qualitative synthesis  2. Qualitative synthesis | Strong evidence in favour of exercise in terms of muscle power function, exercise tolerance functions and mobility-related activities |
| (159) | High | y | 1. Mortality  2. Serious adverse events  3. Health-related QOL  4. Exercise capacity | 1.RR 1.00, 95% CI 0.06 to 15.78  2.RR 1.01, 95% CI 0.98 to 1.05  3.MD 1.96, 95% CI -2.50 to 6.42  4.SMD 0.86, 95% CI 0.46 to 1.26 | Mainly very low certainty evidence |
| (160) | High | n | 1. Progression of scoliosis (lumbar)  2. Progression of scoliosis (thoracic) | 1. MD 9.00 95% CI 5.47 to 12.53  2. MD 8.00 95% CI 5.08 to 10.92 | Low certainty evidence |
| (161) | High | y | 1.Activity Gross motor function  2. Activity Gait speed | 1. SMD 0.53, 95% CI 0.02 to 1.04  2. MD 0.09, 95% CI −0.11 to 0.28 | Low to very low certainty evidence |
| (162) | Low | y | 1.Pain  2.Disability  3.Adverse events | 1. MD 7.43, 95% CI 10.47 to 4.40  2. MD 4.84, 95% CI 7.02 to 2.65  3. See table 4. | Mainly high certainty evidence |
| (163) | High | y | 1. Case fatality  2. Disability  3. Physical fitness  4. Mobility | 1. See table 4.  2. SMD 0.21, 95%CI -0.10 to 0.52  3. MD 2.86, 95% CI 1.76 to 3.96  4. MD 6.71, 95%CI 2.73 to 10.69 | Mainly high certainty evidence |
| (164) | Unclear | y | 1.Bone area  2.Bone mineral content (forearm)  3. Body weight gain  4. Head circumference | 1.MD 1.38, 95% CI 0.70 to 2.07  2.MD 130.91, 95% CI 55.35 to 206. 47  3.MD 2.21, 95% CI 1.23 to 3.19  4.MD -0.03, 95%CI -0.14 to 0.08 | / |
| (165) | High | n | 1. Total cardiovascular risk  2. Total cholesterol  3. LDL and HDL cholesterol  4.Blood pressure  5.BMI | 1. Qualitative synthesis  2. Qualitative synthesis  3. Qualitative synthesis  4. Qualitative synthesis  5. Qualitative synthesis | Poor reporting; half of the trials failed to describe randomisation method used |
| (166) | High | y | 1. Weight change  2. Change in BMI  3. Change in systolic blood pressure  4. Change in diastolic blood pressure  5. Change in fasting serum glucose | 1. MD -2.03, 95% CI -2.82 to -1.23  2. MD -0.73, 95% CI -0.99 to -0.46  3. MD -0.59, 95% CI -2.66 to 1.49  4. MD -2.09, 95%CI -3.68 to -0.51  5. MD -0.17, 95% CI -0.30 to -0.05 | Higher intensity exercise resulted in greater reduction in fasting serum glucose than lower intensity exercise |
| (167) | Unclear | y | 1. Gestational diabetes  2. Pre-eclampsia  3. Pregnancy-induced hypertension/hypertension  4. Caesarean section  5. Perineal trauma  6. Gestational weight gain | 1.RR 0.85, 95%CI 0.71 to 1.01  2. RR 0.98, 95%CI 0.79 to 1.22  3. RR 0.78, 95%CI 0.47 to 1.27  4. RR 0.95, 95%CI 0.88 to 1.02  5. RR 1.27, 95%CI 0.78 to 2.05  6. MD -0.89, 95%CI -1.39 to -0.40 | Very low to moderate certainty evidence |
| (168) | High | y | 1. Mortality  2. Serious adverse events  3. Exercise capacity  4. Return to work | 1. RR 4.46, 95%CI 0.22 to 90.78  2. RR 1.15, 95%CI 0.37 to 3.62  3. SMD -0.47, 95%CI -0.81 to -0.13  4. RR 0.55, 95%CI 0.19 to 1.56 | Mainly very low certainty evidence |
| (169) | High | y | 1.Inspiratory muscle strength  2.Exacerbations  3.PEmax  4.FEV1  5.Dyspnoea  6.Use of beta2-agonist | 1.MD 13.34, 95%CI 4.7 to 21.98  2.-  3. MD 14.46, 95%CI -2.93 to 31.84  4.-  5.-  6.- | Mainly very low certainty evidence |
| (170) | Low | y | 1.Gait speed  2.Timed-up –and-go test  3.Six-minute-walk test  4. Barthel index | 1. MD 0.07, 95% CI 0.05 to 0.10  2. MD 1.81, 95% CI -2.29 to -1.33  3. MD 26.06, 95% CI 7.14 to 44.97  4. MD -0.07, 95% CI -0.68 to 0.53 | Total sample size <500 participants |
| (171) | Unclear | y | 1.Joint health  2.Pain  3.Range of motion  4.Strength | 1. MD -1.21, 95% CI - 2.14 to -0.28  2. Qualitative synthesis  3. Qualitative synthesis  4. Qualitative synthesis | Mainly very low certainty evidence |
| (172) | Low | y | 1.Functional ability  2.Quality of life  3.Aerobic capacity | 1. WMD -0.07, 95% CI -0.22 to 0.08  2. WMD -3.96, 95% CI -8.91 to 1.00  3. WMD 0.04, 95% CI -0.11 to 0.19 | All three trials met at least seven criteria on the PEDro scale |
| (173) | Low-unclear | y | 1.Mortality  2. Overall hospitalization  3.Health related QOL | 1.RR 0.93, 95% CI 0.69 to 1.27  2.RR 0.75, 95% CI 0.62 to 0.92  3.MD-5.8, 95% CI -9.2 to -2.4 | Large treatment effect for 3. in studies judged to be overall higher risk of bias |
| (174) | Unclear to high | y | 1. Glycated haemoglobin levels  2. Whole body mass  3. Visceral adipose tissue  4. Insulin response  5. Plasma triglycerides | 1. MD -0.62, 95% CI -0.91 to -0.33  2. MD -0.04, 95% CI -3.83 to 3.76  3. -  4. MD -0.71, 95% CI -4.13 to 2.71  5. MD -0.25, 95% CI -0.48 to -0.02 | / |
| (175) | Unclear to low | n | 1. Smoking cessation at longest follow-up | 1. Qualitative synthesis | Most studies favoured exercise |
| (176) | High | y | 1. Age of onset of independent walking  2. Age of onset of walking with assistance  3. Gross motor function  4. Gross motor function related to standing  5. Gross motor function related to walking, running and jumping  6. Velocity | 1.MD -2.08, 95% CI -5.38 to 1.22  2.MD -38.54, 95% CI -106.13 to 29.05  3.MD 0.88, 95% CI -4.54 to 6.30  4.MD 5.41, 95% CI -1.64 to 12.43  5.MD 4.51, 95% CI 0.29 to 8.73  6.MD 0.23, 95% CI 0.08 to 0.37 | High to low certainty evidence |
| (177) | High | y | 1.Pain during activity  2.Functional ability  3. Recovery | 1. MD -1.46, 95% CI -2.39 to -0.54  2. SMD 1.10, 95% CI 0.58 to 1.63  3. RR 1.35, 95% CI 0.99 to 1.84 | Very low certainty evidence for all outcomes |
| (178) | High | y | 1. ADL measures  2. Caregiver Strain Index  3. Gait speed  4. Mood and QoL  5. Length of stay  6. Adverse outcomes | 1.SMD 0.21, 95% CI -0.02 to 0.44  2.MD -0.50, 95% CI -1.81 to 0.81  3.MD 0.08, 95% CI -0.03 to 0.18  4.MD 18.2, 95% CI 7.54 to 28.86  5.MD 12.0, 95% CI -10.88 to 34.88  6.MD 0.04, 95% CI -0.10 to 0.18 | Very low to moderate certainty evidence |
| (179) | Unclear | n | 1. Dynamic muscle strength  2. Aerobic capacity | 1. MD 1.17, 95% CI 0.18 to 2.16  2. MD 14.6, 95% CI -0.96 to 30.16 | Moderate certainty evidence |
| (180) | High | n | 1. Change in time taken for 6m comfortable walk  2. Change in isokinetic knee extension torque  3. Change in endurance  4. Change in isokinetic knee flexion torque  5. Change in maximal isometric voluntary contraction force | 1. MD 0.7, 95% CI 0.23 to 1.17  2. MD 17.7, 95% CI 5.11 to 30.29  3. MD 0.3, 95% CI -11.04 to 11.64  4. MD 0.5, 95% CI -9.78 to 8.78  5. MD 12.6, 95% CI -1.51 to 26.71 | Moderate certainty evidence for all outcomes |
| (181) | High | y | 1. Back-specific function  2. Pain  3. Adverse events | 1. SMD of -0.45, 95% CI -0.71 to - 0.19  2. MD 10.83, 95% CI 20. 85 to 0.81  3. RD 0.05, 95% CI 0.02 to 0.08 | Moderate to very low certainty evidence |
| (182) | High | y | 1. Strength  2. Flexibility  3. Balance  4. Adverse events | 1. MD 21.1, 95% CI 8.47 to 33.74  2. MD 2.05, 95% CI 0.59 to 3.51  3. SMD 0.44, 95% CI 0.08 to 0.79  4. SMD 0.81, 95% CI 0.34 to 1.29 | Mainly very low certainty evidence |
| (183) | High | y | 1.Hand function  2.Pain  3.Hand impairment  4.Adherence  5. Adverse events | 1. Narrative synthesis  2. MD -27.98, 95% CI -48.93 to -7. 03  3. SMD 0.44, 95% CI 0.11 to 0.78  4. RR 1.31, 95% CI 1.15 to 1.48  5.See table 4. | High to very low certainty evidence |
| (184) | High | y | 1. Quality of life | 1.SMD 0.88, 95% CI -0.12 to 1.88 | Very low certainty evidence; only two studies with 40 participants |
| (185) | High | y | 1. Pain  2. Disability  3. Function  4. Global impression of recovery  5. Adverse events | 1. MD -14.05, 95% CI -18.91 to -9.19  2. MD -10.54, 95% CI -18.46 to -2.62  3. MD 1.10, 95% CI 0.23 to 1.97  4. MD 1.50, 95% CI 0.70 to 2.30  5. See table 4. | Mainly low certainty evidence |
| (186) | High | y | 1.Quality of life  2.Asthma symptoms  3.Asthma control  4.FEV1  5.Medication usage  6.Adverse events | 1. MD 0.57, 95% CI 0.37 to 0.77  2. SMD 0.37, 95% CI 0.09 to 0.65  3. Narrative synthesis  4. MD 0.04, 95% CI -0.10 to 0.19  5.RR 5.35, 95% CI 1.29 to 22.11  6. See table 4. | Moderate to very low certainty evidence |
| (187) | High | y | 1. Cognitive speed  2. Visual memory functions  3. Working memory  4. Memory functions  5. Executive functions | 1.SMD 0.12, 95% CI -0.08 to 0.33  2.SMD -0.26, 95% CI -0.97 to 0.44  3. SMD 0.10, 95% CI -0.16 to 0.36  4. SMD 0.10, 95% CI -0.16 to 0.35  5.SMD 0.38, 95% CI -0.14 to 0.90 | / |
| (188) | Unclear | y | 1.Peak exercise  2.Exercise tolerance  3. Health related QOL | 1.MD -0.55, 95% CI -2.84 to 1.74  2. MD 3.70, 95%CI -3.38 to 10.78  3. MD 2.51; 95% CI -1.32 to 6.34 | Moderate to low certainty evidence |
| **Total** | High (n=109)  Low (n=24)  Unclear (n=28) | Yes (n=129)  No (n=21) | / | / | Moderate (n=29)  High (n=8) |

**Table 2 Footnote:**

ADL: activities in daily life, BMI: Body mass index, BW: Body weight, CI: confidence interval, CONSORT: Consolidated Standards of Reporting Trials, CRQ: Chronic Respiratory Disease Questionnaire, CVD: cardiovascular disease, FEV1: forced expiratory volume in 1 second, FVC: forced vital capacity, HRQL: health-related quality of life, ICU: intensive care unit, LBP: low-back pain, MD: mean difference, RCT: randomised controlled trial, RaR: rate ratio, RR: risk ratio, SMD: standard mean difference, WMD: weight mean difference, VO2max: maximal oxygen uptake/consumption

- or / = Not applicable

* Verhagen AP, de Vet HC, de Bie RA, Kessels AG, Boers M, Bouter LM, et al.The Delphi list: a criteria list for quality assessment of randomized clinical trials for conducting systematic reviews developed by Delphi consensus. Journal of Clinical Epidemiology 1998;51(12):1235–41.

**Supplementary Table 3.** **Conclusions from Cochrane systematic reviews “quote”**

| **Reference** | **Review authors’ conclusions** | **Direction of conclusion (-/+)** | **Comment** |
| --- | --- | --- | --- |
| (39) | “This review found one randomized trial that showed that weight loss may be beneficial for improving asthma control in overweight and obese patients, in conjunction with weight loss in intervention groups in the short term…On account of this low quality of evidence, the benefit of weight loss as an intervention for asthma control remains uncertain.” | -/+ | A need for well-designed studies in children and adolescents, as well as in low-income countries was emphasized |
| (40) | “We found low quality evidence that multidisciplinary interventions involving a combination of diet, physical activity and behavioural components reduce measures of BMI and moderate quality evidence that they reduce weight in overweight or obese adolescents, mainly when compared with no treatment or waiting list controls. Inconsistent results, risk of bias or indirectness of outcome measures used mean that the evidence should be interpreted with caution.” | -/+ | A large number of ongoing trials has been identified and will be included in the update review |
| (41) | “Evidence from this review suggests that both diet and exercise together and diet alone help females to lose weight after childbirth. Nevertheless, it may be preferable to lose weight through a combination of diet and exercise as this improves maternal cardiorespiratory fitness and preserves fat-free mass, while diet alone reduces fat-free mass. This needs confirmation in large trials of high methodological quality.” | -/+ | There was insufficient evidence to judge whether exercise or diet interfere with breastfeeding though it appeared not to in the included studies |
| (42) | “We found moderate quality evidence suggesting that exercise-based cardiac rehabilitation improves exercise capacity… Cardiac rehabilitation appears to be safe in this population, but long-term follow-up data are incomplete and further good quality and adequately powered trials are needed to demonstrate the longer-term benefits of exercise on safety and impact on both clinical and patient-related outcomes...” | + | Further research was suggested to establish long-term impacts of exercise-based rehabilitation on risk of death and hospital admission |
| (43) | “This updated Cochrane review supports the conclusions of the previous version of this review that, compared with no exercise control, exercise-based cardiac rehabilitation reduces the risk of cardiovascular mortality but not total mortality. We saw a significant reduction in the risk of hospitalisation with cardiac rehabilitation but not in the risk of MI (myocardial infarction) or revascularisation… More recent trials were more likely to be well reported and include older and female patients. However, the population studied in this review still consists predominantly of lower risk individuals following MI or revascularisation.” | + | Exercise-based cardiac rehabilitation significantly reduced cardiovascular mortality, and improved life quality compared with controls |
| (44) | “There is insufficient evidence to demonstrate that aerobic exercise in adults with Down syndrome improves physical or psychosocial outcomes. Although evidence exists to support improvements in physiological and psychological aspects from strategies using mixed physical activity programmes, well-conducted research examining long-term physical outcomes, adverse effects, psychosocial outcomes and costs is required before informed practice decisions can be made.” | -/+ | All studies had a high risk of bias for blinding |
| (45) | “There is currently insufficient evidence available to assess the efficacy of physical exercise in people with CVI. Future research into the effect of physical exercise should consider types of exercise protocols (intensity, frequency and time), sample size, blinding and homogeneity according to the severity of disease.” | - | / |
| (46) | “In the short-term, center-based programs are superior to home-based programs in patients with PVD (peripheral vascular disease). There is a high possibility of a training effect however as the center-based groups were trained primarily on treadmills (and the home based were not) and the outcome measures were treadmill based. There is conflicting evidence which is better in patients with COPD (Chronic Obstructive Pulmonary Disease). Home based programs appear to be superior to center based programs in terms of the adherence to exercise (especially in the long-term).” | -/+ | Conclusions drawn from a small number of poor-quality studies conducted mainly in the 1990-ies |
| (47) | “There is moderate quality evidence that aquatic exercise may have small, short-term, and clinically relevant effects on patient-reported pain, disability, and QoL in people with knee and hip OA. The conclusions of this review update does not change those of the previous published version of this Cochrane review.” | + | Short-term effectiveness established (up to 12 weeks of aquatic exercises) |
| (48) | “This review indicates that swimming training is well-tolerated in children and adolescents with stable asthma, and increases lung function (moderate strength evidence) and cardio-pulmonary fitness (high strength evidence). There was no evidence that swimming training caused adverse effects on asthma control in young people 18 years and under with stable asthma of any severity. However whether swimming is better than other forms of physical activity cannot be determined from this review. Further adequately powered trials with longer follow-up periods are needed to better assess the long-term benefits of swimming.” | -/+ | / |
| (49) | “There is no evidence for differences in mortality between the exercise and control groups. Physical exercise added to standard care can improve quality of life, especially physical functioning, depression and fatigue. Currently, there is inconclusive evidence regarding anxiety, physical performance, serious adverse events and adverse events.” | -/+ | Eight studies evaluated various aspects of physical performance (e.g. aerobic capacity, cardiovascular fitness), but none of them could be pooled in a meta-analysis. |
| (50) | “When compared with control, moderate-quality evidence indicates that aerobic exercise probably improves HRQL (health-related quality of life) and all-cause withdrawal, and low-quality evidence suggests that aerobic exercise may slightly decrease pain intensity, may slightly improve physical function, and may lead to little difference in fatigue and stiffness. Three of the reported outcomes reached clinical significance (HRQL, physical function, and pain). Long-term effects of aerobic exercise may include little or no difference in pain, physical function, and all-cause withdrawal, and we are uncertain about long-term effects on remaining outcomes. […] Aerobic exercise appears to be well tolerated (similar withdrawal rates across groups), although evidence on adverse events is scarce, so we are uncertain about its safety.” | -/+ | The evidence was downgraded due to the small number of included trials and participants across trials, and because of issues related to unclear and high risks of bias |
| (51) | “Whether WBV (whole body vibration) or WBV in addition to mixed exercise is superior to control or another intervention for Females with fibromyalgia remains uncertain. The quality of evidence is very low owing to imprecision (few study participants and wide confidence intervals) and issues related to risk of bias. These trials did not measure major outcomes such as pain intensity, stiffness, fatigue, and physical function. Overall, studies were few and were very small, which prevented meaningful estimates of harms and definitive conclusions about WBV safety.” | -/+ | Absolute improvement in the intervention was 4% (95% CI 11% better to 3% worse) |
| (52) | “Low to moderate quality evidence relative to control suggests that aquatic training is beneficial for improving wellness, symptoms, and fitness in adults with fibromyalgia. Very low to low quality evidence suggests that there are benefits of aquatic and land-based exercise, except in muscle strength (very low-quality evidence favoring land).” | -/+ | For the review main comparison, the evidence was predominantly of low quality |
| (53) | “The effects of physical exercise training interventions for childhood cancer participants are not yet convincing. Possible reasons are the small numbers of participants and insufficient study designs, but it can also be that this type of intervention is not as effective as in adult cancer patients. However, the first results show some positive effects on physical fitness in the intervention group compared to the control group. There were positive intervention effects for body composition, flexibility, cardiorespiratory fitness, muscle strength, and health-related quality of life (cancer-related items). These were measured by some assessment methods, but not all.” | -/+ | Conclusions drawn from a small number of studies. Heterogeneity of interventions |
| (54) | “We did not find support for an effect of dance/movement therapy on depression, stress, anxiety, fatigue and body image. The findings of individual studies suggest that dance/movement therapy may have a beneficial effect on QoL, somatization, and vigor. However, the limited number of studies prevents us from drawing conclusions concerning the effects of dance/movement therapy on psychological and physical outcomes in cancer patients.” | -/+ | Insufficient number of studies; more research was recommended |
| (55) | “We found minimal differences between yoga and non-standard care, the latter consisting of another exercise comparator, which could be broadly considered aerobic exercise. Outcomes were largely based on single studies with limited sample sizes and short-term follow up. Overall, many outcomes were not reported and evidence presented in this review is of low to moderate quality - too weak to indicate that yoga is superior or inferior to non-standard care control for management of people with schizophrenia.” | -/+ | More larger, and long-term trials was recommended |
| (56) | “Even though we found some positive evidence in favour of yoga over standard-care control, this should be interpreted cautiously in view of outcomes largely based each on one study with limited sample sizes and short-term follow-up. Overall, many outcomes were not reported and evidence presented in this review is of low to moderate quality - -too weak to indicate that yoga is superior to standard care control for the management of schizophrenia.” | -/+ | / |
| (57) | “A small number of small studies were included in this review and these lacked many key outcomes. The sparse data means we cannot state with any degree of certainty if yoga delivered as a package of care is beneficial in comparison to standard care.” | -/+ | Very small number of trials; ‘slicing salami phenomenon’ can be observed (please refer to reviews 17-18) |
| (58) | “Short- and long-term outcomes of interest for this review were poorly reported. Current evidence is confounded by the large variety of exercise interventions. There was insufficient high-quality evidence to be able to determine any differences between exercise and control groups for our outcomes of interest. For the woman, both fasting and postprandial blood glucose concentrations were reduced compared with the control groups. There are currently insufficient data for us to determine if there are also benefits for the infant.” | -/+ | The quality of the evidence ranged from high to low. More research was recommended with both the short- and long-term outcomes |
| (59) | “There is ’gold’ level evidence (www.cochranemsk.org) that supervised aerobic exercise training has beneficial effects on physical capacity and FM (Fibromyalgia) symptoms. Strength training may also have benefits on some FM symptoms. Further studies on muscle strengthening and flexibility are needed. Research on the long-term benefit of exercise for FM is needed.” | + | The effect sizes for all primary outcomes were small to medium |
| (60) | “The evidence (rated as low quality) suggested that moderate- and moderate- to high-intensity resistance training improves multidimensional function, pain, tenderness, and muscle strength in Females with fibromyalgia. The evidence (rated as low quality) also suggested that eight weeks of aerobic exercise was superior to moderate-intensity resistance training for improving pain in Females with fibromyalgia. There was low-quality evidence that 12 weeks of low-intensity resistance training was superior to flexibility exercise training in Females with fibromyalgia for improvements in pain and multidimensional function. There was low-quality evidence that Females with fibromyalgia can safely perform moderate- to high-resistance training.” | + | Determination of the minimum clinically important difference for future research was stressed |
| (61) | “In care facilities: we are uncertain of the effect of exercise on rate of falls and it may make little or no difference to the risk of falling. General medication review may make little or no difference to the rate of falls or risk of falling […]” | - | Future RCTs were recommended to reduce the uncertainties |
| (62) | “This review demonstrated that physical training showed significant improvement in maximum oxygen uptake, though no effects were observed in other measures of pulmonary function. Physical training was well tolerated among people with asthma in the included studies and, as such, people with stable asthma should be encouraged to participate in regular exercise training, without fear of symptom exacerbation.” | + | Given the low and very low certainty of the evidence, the conclusions seem overinflated |
| (63) | “Limited evidence from two RCTs demonstrated that PRT (progressive resistance training) is more effective than standard physiotherapy treatment for shoulder dysfunction in patients treated for head and neck cancer, improving pain, disability and range of motion of the shoulder joint, but it does not improve quality of life. However, although statistically significant the measured benefits of the intervention may be small. Other exercise regimes were not shown to be effective compared to routine postoperative physiotherapy. Further studies which apply other exercise interventions in head and neck cancer patients in the early postoperative and radiotherapy period are needed, with long term follow-up.” | -/+ | The quality of the evidence was classified as moderate |
| (64) | “Preoperative exercise training may reduce the risk of developing a postoperative pulmonary complication, the duration of intercostal catheter use, postoperative length of hospital stay, and improve both exercise capacity and FVC (forced vital capacity) in people undergoing lung resection for NSCLC. The findings of this review should be interpreted with caution due to disparities between the studies, risk of bias, and small sample sizes. This review emphasises the need for larger RCTs.” | + | Small number of methodologically weak studies, and total sample size prevent firm conclusions |
| (65) | “The evidence summarised in our review suggests that exercise training may potentially increase the exercise capacity of people following lung resection for NSCLC. The findings of our systematic review should be interpreted with caution due to disparities between the studies, methodological limitations, some significant risks of bias and small sample sizes. This systematic review emphasises the need for larger RCTs” | -/+ | Lack of blinding, and the need for intention-to-treat analyses were highlighted |
| (66) | “There is insufficient evidence to recommend, or advise against, diabetic pregnant Females to enrol in exercise programs. Further trials, with larger sample size, involving Females with gestational diabetes, and possibly type 1 and 2 diabetes, are needed to evaluate this intervention.” | -/+ | Six trials were awaiting classification; risk of bias was poorly reported |
| (67) | “There is moderate quality evidence that post-treatment exercise programmes can prevent recurrences of back pain but conflicting evidence was found for treatment exercise. Studies into the validity of measurement of recurrences and the effectiveness of post-treatment exercise are needed.” | + | There were differences in exercises across studies |
| (68) | “Muticomponent interventions appear to be an effective treatment option for overweight or obese preschool children up to the age of years. However, the current evidence is limited, and most trials had a high risk of bias. Most trials did not measure adverse events. We have identified four ongoing trials that we will include in future updates of this review.” | -/+ | There were few studies per outcome or the number children was small; attrition rate was high |
| (69) | “At this time, we are unable to determine an overall effect on functional exercise capacity, or on health-related quality of life, of an exercise-based intervention initiated after ICU (intensive care unit) discharge for survivors of critical illness. […] Some studies reported a beneficial effect of the intervention on functional exercise capacity, and others did not. No effect on health-related quality of life was reported.” | -/+ | Methodological rigor was lacking across several domains, influencing the quality of the evidence. |
| (70) | “Exercise is moderately more effective than a control intervention for reducing symptoms of depression, but analysis of methodologically robust trials only shows a smaller effect in favour of exercise. When compared to psychological or pharmacological therapies, exercise appears to be no more effective, though this conclusion is based on a few small trials.” | -/+ | Types of exercise, the number and duration of sessions which are of most benefit remains unclear |
| (71) | “We found that Constraint-induced movement therapy was associated with limited improvements in motor impairment and motor function, but that these benefits did not convincingly reduce disability. This differs from the result of our previous meta-analysis where there was a suggestion that CIMT might be superior to traditional rehabilitation. Information about the long-term effects of CIMT is scarce. Further trials studying the relationship between participant characteristics and improved outcomes are required.” | -/+ | Not all risk of bias domains were assessed |
| (72) | “Moderate-quality evidence supports the recommendation of yoga as a supportive intervention for improving health-related quality of life and reducing fatigue and sleep disturbances when compared with no therapy, as well as for reducing depression, anxiety and fatigue, when compared with psychosocial/educational interventions. Very low-quality evidence suggests that yoga might be as effective as other exercise interventions and might be used as an alternative to other exercise programmes.” | + | Heterogeneous comparators; several gaps in available evidence were identified |
| (73) | “The findings of the updated review have enabled a more precise conclusion to be made in that aerobic exercise can be regarded as beneficial for individuals with cancer-related fatigue during and post-cancer therapy, specifically those with solid tumours. Further research is required to determine the optimal type, intensity and timing of an exercise intervention.” | + | A tendency to over-emphasise the findings based on statistical significance |
| (74) | “The included studies were too small to determine to what extent strengthening exercises for people with ALS (amyotrophic lateral sclerosis) are beneficial, or whether exercise is harmful. There is a complete lack of randomised or quasi-randomised clinical trials examining aerobic exercise in this population. More research is needed.” | -/+ | Conclusions based on two studies |
| (75) | “The evidence examining exercise training in people with non-malignant dust-related respiratory diseases is of very low quality. This is due to imprecision in the results from the small number of trials and the small number of participants, the indirectness of evidence due to a paucity of information on disease severity and the data from one study being from a subgroup of participants, and inconsistency from high heterogeneity in some results. Therefore, although the review findings indicate that an exercise training programme is effective in improving exercise capacity and health-related quality of life in the short-term and at six months follow-up, we remain unsure of these findings due to the very low-quality evidence” | -/+ | With only 40 participants, the need for more, high quality trials was highlighted |
| (76) | “Evidence was insufficient to show whether exercise is an effective treatment for vasomotor menopausal symptoms. One small study suggested that HT is more effective than exercise. Evidence was insufficient to show the relative effectiveness of exercise when compared with HT or yoga.” | -/+ | Heterogeneous comparators; poor reporting |
| (77) | “There is ’silver’ level evidence (www.cochranemsk.org) that multidisciplinary intervention that includes exercise may increase the proportion of patients discharged to home and reduce length and cost of hospital stay for acutely hospitalised older medical patients.” | -/+ | There was not enough evidence on the benefits and harms of exercise sessions |
| (78) | “The evidence suggests the ongoing implementation of school-based physical activity interventions at this time, given the positive effects on behavior and one physical health status measure. However, given these studies are at a minimum of moderate risk of bias, and the magnitude of effect is generally small, these results should be interpreted cautiously. Additional research on the long-term impact of these interventions is needed.” | + | Reduced methodological quality of the evidence; cautious interpretation of the findings was advised |
| (79) | “There is insufficient evidence on the effect of early mobilization of critically ill people in the ICU (intensive care unit) on physical function or performance, adverse events, muscle strength and health-related quality of life at this time. […] We assessed that there is currently low-quality evidence for the effect of early mobilization of critically ill adults in the ICU due to small sample sizes, lack of blinding of participants and personnel, variation in the interventions and outcomes used to measure their effect and inadequate descriptions of the interventions delivered as usual care in the studies included in this Cochrane Review.” | - | The four studies awaiting classification, and the three ongoing studies may alter the conclusions of the review |
| (80) | “The results indicate that exercise has positive short-term effects on self-esteem in children and young people. Since there are no known negative effects of exercise and many positive effects on physical health, exercise may be an important measure in improving children’s self-esteem.” | + | Conclusions are based on several small low-quality trials |
| (81) | “[…] No evidence was found for the effectiveness of […] exercise. Studies are needed to investigate the effect of exercise intensity on exercise capacity and subjective fatigue. Future studies should focus on interventions that address the maladaptive behavioural or cognitive aspects of fatigue in people with PD. Characteristics, such as severity and nature of perceived fatigue and underlying mood disorders should be considered to identify responders and non-responders when studying interventions for fatigue […].” | - | The effect size was small; statistically not significant |
| (82) | “The currently available data provide little information about the effectiveness of yoga interventions for people suffering from haematological malignancies. The finding that yoga may be beneficial for the patients’ quality of sleep is based on a very small body of evidence. Therefore, the role of yoga as an additional therapy for haematological malignancies remains unclear. Further high-quality randomised controlled trials with larger numbers of participants are needed to make a definitive statement.” | -/+ | Finding must be interpreted cautiously |
| (83) | “There is promising evidence that exercise programs may improve the ability to perform ADLs (activities in daily life) in people with dementia, although some caution is advised in interpreting these findings. The review revealed no evidence of benefit from exercise on cognition, neuropsychiatric symptoms, or depression. There was little or no evidence regarding the remaining outcomes of interest (i.e., mortality, caregiver burden, caregiver quality of life, caregiver mortality, and use of healthcare services).” | + | Caution is advised when interpreting the findings |
| (84) | “High-quality evidence indicates that land-based therapeutic exercise provides short-term benefit that is sustained for at least two to six months after cessation of formal treatment in terms of reduced knee pain, and moderate-quality evidence shows improvement in physical function among people with knee OA (osteoarthritis). The magnitude of the treatment effect would be considered moderate (immediate) to small (two to six months) but comparable with estimates reported for non-steroidal anti-inflammatory drugs. Confidence intervals around demonstrated pooled results for pain reduction and improvement in physical function do not exclude a minimal clinically important treatment effect. Since the participants in most trials were aware of their treatment, this may have contributed to their improvement.” | + | The authors did not downgrade the quality of evidence despite the lack of blinding |
| (85) | “Pooling the results of these 10 RCTs demonstrated that land-based therapeutic exercise programmes can reduce pain and improve physical function among people with symptomatic hip OA (osteoarthritis).” | + | Research to assess the long-term effectiveness of exercise for people with hip OA was recommended |
| (86) | “Even though individual trials reported positive effects of breathing exercises, no reliable conclusions could be drawn concerning the use of breathing exercises for asthma in clinical practice. This was a result of methodological differences among the included studies and poor reporting of methodological aspects in most of the included studies. However, trends for improvement are encouraging, and further studies including full descriptions of treatment methods and outcome measurements are required.” | -/+ | Meta-analysis was possible for only two outcomes (asthma symptoms and change in Asthma Quality of Life Questionnaire) |
| (87) | “[…] The findings of the updated review have enabled us to make a more precise conclusion that both aerobic and resistance exercise can be regarded as beneficial for individuals with adjuvant therapy-related side effects. Further research is required to determine the optimal type, intensity, and timing of an exercise intervention. Furthermore, long-term evaluation is required due to possible long-term side effects of adjuvant treatment.” | + | Considerable degree of clinical heterogeneity; high risk for performance and detection bias |
| (88) | “No definitive conclusions can be made regarding the benefits of exercise for individuals with vertebral fracture. Although individual trials did report benefits for some pain, physical function and quality of life outcomes, the findings should be interpreted with caution given that findings were inconsistent and the quality of evidence was very low. The small number of trials and variability across trials limited our ability to pool outcomes or make conclusions. Evidence regarding the effects of exercise after vertebral fracture, particularly for Males, is scarce. A high-quality randomized trial is needed to inform exercise prescription for individuals with vertebral fractures.” | -/+ | Poor reporting in primary trials was noted |
| (89) | “Group and home-based exercise programmes, and home safety interventions reduce rate of falls and risk of falling. Multifactorial assessment and intervention programmes reduce rate of falls but not risk of falling; Tai Chi reduces risk of falling […]” | + | The effect size was large |
| (90) | “Results of this Cochrane review are similar to existing reviews that have examined the health benefits of exercise in this population. Although studies included in this review are small and used various measures of physical and mental health, results indicated that regular exercise programmes are possible in this population, and that they can have healthful effects on both the physical and mental health and well-being of individuals with schizophrenia. Larger randomised studies are required before any definitive conclusions can be drawn.” | -/+ | Conclusions based on three biased studies |
| (91) | “We cannot determine whether exercise is effective at altering the occurrence, severity or duration of acute respiratory infections. One analysis of four trials suggests that the number of days of illness per episode of infection might be reduced by exercise. […].” | -/+ | The small size of the studies, risk of bias and heterogeneous populations all contributed to the low certainty of the evidence |
| (92) | “From the available evidence, we found that exercising before influenza vaccination is neither beneficial nor harmful. However, study data were limited and of low quality. Small sample sizes, study design limitations, exercise types, and focus on biochemical rather than participant-centred outcomes strongly influenced our findings.” | -/+ | Only one study under the main comparison |
| (93) | “The small number of participants in the three included studies, the clinical and methodological heterogeneity observed and the high risk of bias assessed mean that we are unable to assess the place of water-based exercise in asthma. Randomised controlled trials are needed to assess the efficacy and safety of water-based exercise for adults with asthma. For future research, we suggest greater methodological rigour (participant selection, blinding of outcome assessors, reporting of all outcomes analysed and registering of the study protocol).” | -/+ | Very small number of highly biased studies; cautious interpretation recommended |
| (94) | “No high-quality evidence was found, indicating that there is still uncertainty about the effectiveness of exercise for neck pain. Using specific strengthening exercises as a part of routine practice for chronic neck pain, cervicogenic headache and radiculopathy may be beneficial. Research showed the use of strengthening and endurance exercises for the cervico-scapulothoracic and shoulder may be beneficial in reducing pain and improving function. However, when only stretching exercises were used no beneficial effects may be expected. Future research should explore optimal dosage.” | -/+ | The number of participants in most trials was small, more than half of the included studies were either of low or very low quality |
| (95) | “Evidence of moderate and high-quality shows that SET provides an important benefit for treadmill-measured walking distance (MWD and PFWD) compared with HBET and WA, respectively. Although its clinical relevance has not been definitively demonstrated, this benefit translates to increased MWD of 120 and 210 meters after three months in SET groups. These increased walking distances are likely to have a positive impact on the lives of patients with IC. Data provide no clear evidence of a difference between HBET and WA. Trials show no clear differences in quality of life parameters nor in self-reported functional impairment between SET and HBET. However, evidence is of low and very low quality, respectively. Investigators detected some improvements in quality of life favoring SET over WA, but analyses were limited by small numbers of studies and participants. Future studies should focus on disease-specific quality of life and other functional outcomes, such as walking behavior and physical activity, as well as on long-term follow-up.” | + | / |
| (96) | “The results suggest Tai Chi does not exacerbate symptoms of rheumatoid arthritis. In addition, Tai Chi has statistically significant benefits on lower extremity range of motion, in particular ankle range of motion, for people with RA. The included studies did not assess the effects on patient-reported pain.” | + | Cautious interpretation advised; small number of poor-quality studies |
| (97) | “There is limited randomised controlled trial evidence available on the effect of exercise during pregnancy for preventing pregnancy glucose intolerance or GDM (Gestational diabetes mellitus). Results from three randomised trials with moderate risk of bias suggested no significant difference in GDM incidence between Females receiving an additional exercise intervention and routine care.  Based on the limited data currently available, conclusive evidence is not available to guide practice. Larger, well-designed randomised trials, with standardised behavioural interventions are needed to assess the effects of exercise on preventing GDM and other adverse pregnancy outcomes including large-for-gestational age and perinatal mortality. Longer-term health outcomes for both Females and their babies and health service costs should be included […]” | -/+ | Small number of studies at high risk of bias Several such trials are in progress. Authors identified seven ongoing trials |
| (98) | “The limited evidence comes from small, short-term, low-quality studies. There is some evidence that yoga has favourable effects on diastolic blood pressure, HDL cholesterol and triglycerides, and uncertain effects on LDL cholesterol. These results should be considered as exploratory and interpreted with caution.” | -/+ | The included studies were of short duration, small and at risk of bias |
| (99) | “There are currently no long-term trials examining tai chi for the primary prevention of CVD (cardiovascular disease). Due to the limited evidence available currently no conclusions can be drawn as to the effectiveness of tai chi on CVD risk factors. There was some suggestion of beneficial effects of tai chi on CVD risk factors but this was not consistent across all studies. There was considerable heterogeneity between the studies included in this review and studies were small and at some risk of bias. Results of the ongoing trials will add to the evidence base but additional longer-term, high-quality trials are needed.” | -/+ | The results should be treated with caution as the studies were small, of short duration and at risk of bias |
| (100) | “Currently, very limited evidence is available on the effectiveness of qigong for the primary prevention of CVD (cardiovascular disease). Most of the trials included in this review are likely to be at high risk of bias, so we have very low confidence in the validity of the results. Publication of the ongoing trial will add to the limited evidence base, but further trials of high methodological quality with sufficient sample size and follow-up are needed to be incorporated in an update of this review before the effectiveness of qigong for CVD prevention can be established.” | -/+ | It was not clear whether the beneficial effects can be attributed to qigong; follow-up ranged from 12 weeks to 20-30 years |
| (101) | “There is low-quality evidence that fitness training is effective at improving cardiorespiratory deconditioning after TBI; there is insufficient evidence to draw any definitive conclusions about the other outcomes. Whilst the intervention appears to be accepted by people with TBI, and there is no evidence of harm, more adequately powered and well-designed studies are required to determine a more precise estimate of the effect on cardiorespiratory fitness, as well as the effects across a range of important outcome measures and in people with different characteristics (e.g. children). In the absence of high-quality evidence, clinicians may be guided by pre-exercise screening checklists to ensure the person with traumatic brain injury is safe to exercise, and set training parameters using guidelines established by the American College of Sports Medicine for people who have suffered a brain injury.” | -/+ | Small numbers of study participants, poor reporting, and possible errors in the conduct of certain trials |
| (102) | “Exercise therapy appears to be slightly effective at decreasing pain and improving function in adults with chronic low-back pain, particularly in healthcare populations. In subacute low-back pain there is some evidence that a graded activity program improves absenteeism outcomes, though evidence for other types of exercise is unclear. In acute low-back pain, exercise therapy is as effective as either no treatment or other conservative treatments.” | + | Acute, subacute and chronic low-back pain populations |
| (103) | “This review found that the existing evidence was insufficient to make any strong recommendations about the best approach to pelvic floor muscle training. We suggest that females are offered reasonably frequent appointments during the training period, because the few data consistently showed that females receiving regular (e.g. weekly) supervision were more likely to report improvement than females doing pelvic floor muscle training with little or no supervision.” | -/+ | Vague conclusions; future research was recommended |
| (104) | “Exercise therapy can be prescribed in people with MS (Multiple sclerosis) without harm. Exercise therapy, and particularly endurance, mixed, or ’other’ training, may reduce self-reported fatigue. However, there are still some important methodological issues to overcome. Unfortunately, most trials did not explicitly include people who experienced fatigue, did not target the therapy on fatigue specifically, and did not use a validated measure of fatigue as the primary measurement of outcome.” | + | Methodological shortcomings in the trials may have affected the reliability of the results |
| (105) | “There is evidence for significant beneficial effects of regular exercise on physical fitness, walking capacity, cardiovascular dimensions (e.g. blood pressure and heart rate), health-related quality of life and some nutritional parameters in adults with CKD. Other outcomes had insufficient evidence due to the lack of data from RCTs. The design of the exercise intervention causes difference in effect size and should be considered when prescribing exercise with the aim of affecting a certain outcome. Future RCTs should focus more on the effects of resistance training interventions or mixed cardiovascular- and resistance training as these exercise types have not been studied as much as cardiovascular exercise.” | + | Outcome measures were not blindly assessed and ITT analysis was not used in all studies |
| (106) | “There is no firm evidence that diet alone or physical activity alone compared to standard treatment influences the risk of T2DM (type 2 diabetes mellitus) and especially its associated complications in people at increased risk of developing T2DM. However, diet plus physical activity reduces or delays the incidence of T2DM in people with IGT. Data are lacking for the effect of diet plus physical activity for people with intermediate hyperglycaemia defined by other glycaemic variables. Most RCTs did not investigate patient-important outcomes.” | -/+ | All trials had weaknesses; and poor reporting. |
| (107) | “The evidence from randomised studies suggests that muscle stretching, whether conducted before, after, or before and after exercise, does not produce clinically important reductions in delayed-onset muscle soreness in healthy adults.” | - | Findings were very consistent |
| (108) | “There is moderate evidence suggesting that back schools, in an occupational setting, reduce pain, and improve function and return-to work status, in the short and intermediate-term, compared to exercises, manipulation, myofascial therapy, advice, placebo or waiting list controls, for patients with chronic and recurrent LBP. However, future trials should improve methodological quality and clinical relevance and evaluate the cost-effectiveness of back schools.” | + | Mean total quality score was 3.8 (low) |
| (109) | “Breathing exercises over four to 15 weeks improve functional exercise capacity in people with COPD compared to no intervention; however, there are no consistent effects on dyspnoea or health-related quality of life. Outcomes were similar across all the breathing exercises examined. Treatment effects for patient-reported outcomes may have been overestimated owing to lack of blinding. Breathing exercises may be useful to improve exercise tolerance in selected individuals with COPD who are unable to undertake exercise training; however, these data do not suggest a widespread role for breathing exercises in the comprehensive management of people with COPD.” | + | Few studies reported details of allocation concealment, assessor blinding or intention-to-treat analysis contributing to overall low-quality evidence |
| (110) | “There is weak evidence that some types of exercise (gait, balance, co-ordination and functional tasks; strengthening exercise; 3D exercise and multiple exercise types) are moderately effective, immediately post intervention, in improving clinical balance outcomes in older people. Such interventions are probably safe. There is either no or insufficient evidence to draw any conclusions for general physical activity (walking or cycling) and exercise involving computerised balance programmes or vibration plates. Further high methodological quality research using core outcome measures and adequate surveillance is required.” | + | The more effective programmes were more intense and frequent |
| (111) | “Our results suggest a relatively small statistically significant, but possibly important, effect of exercise on bone density compared with control groups. Exercise has the potential to be a safe and effective way to avert bone loss in postmenopausal Females.” | + | The quality of the reporting of studies in the meta-analyses was low |
| (112) | “Based on the evidence, aerobic capacity training combined with muscle strength training is recommended as routine practice in patients with RA.” | **+** | Conclusions based on two studies only |
| (113) | “Participation in exercise programmes may slightly improve physical function, depression and pain. It may slightly improve self-efficacy and social function, although there is probably little or no difference in anxiety.” | **-/+** | Conclusions drawn on both qualitative and quantitative data (mixed-methods review) |
| (114) | “There is no credible evidence regarding the effectiveness of breathing exercises for the clinical symptoms of DB/HVS. It is currently unknown whether these interventions offer any added value in this patient group or whether specific types of breathing exercise demonstrate superiority over others.” | **-** | The quality of the evidence was very low |
| (115) | “We found evidence that preoperative inspiratory muscle training was associated with a reduction of postoperative atelectasis, pneumonia, and duration of hospital stay in adults undergoing cardiac and major abdominal surgery.” | **+** | Lack of adequate blinding, small-study effects, and publication bias |
| (116) | “Exercise interventions in community-dwelling older people probably reduce fear of falling to a limited extent immediately after the intervention, without increasing the risk or frequency of falls. There is insufficient evidence to determine whether exercise interventions reduce fear of falling beyond the end of the intervention or their effect on other outcomes.” | **-/+** | The quality of the evidence was predominantly very low |
| (117) | “Regular aerobic exercise during pregnancy appears to improve (or maintain) physical fitness. Available data are insufficient to infer important risks or benefits for the mother or infant.” | **-/+** | The trials are quite small, and none are of high methodological quality |
| (118) | “No conclusions regarding breast cancer-related and all-cause mortality or breast cancer recurrence were possible. However, physical activity interventions may have small-to-moderate beneficial effects on HRQoL, and on emotional or perceived physical and social function, anxiety, cardiorespiratory fitness, and self-reported and objectively measured physical activity.” | -/+ | Variable quality of evidence, heterogeneity of interventions and outcome measures, imprecision of some estimates, and risk of bias in majority of trials |
| (119) | “High-quality evidence shows that exercise programmes provided important benefit compared with placebo or usual care in improving both pain-free and maximum walking distance in people with leg pain from IC who were considered to be fit for exercise intervention. Exercise did not improve ABI, and we found no evidence of an effect of exercise on amputation or mortality. Exercise may improve quality of life when compared with placebo or usual care.” | + | The evidence was not downgraded despite trials being at unclear or high risk of bias |
| (120) | “Patients with CFS may generally benefit and feel less fatigued following exercise therapy, and no evidence suggests that exercise therapy may worsen outcomes. A positive effect with respect to sleep, physical function and self-perceived general health has been observed, but no conclusions for the outcomes of pain, quality of life, anxiety, depression, drop-out rate and health service resources were possible.” | -/+ | All studies had a high risk of performance and detection bias |
| (121) | “Whilst there appears to be a small effect in favour of exercise in reducing depression and anxiety scores in the general population of children and adolescents, the small number of studies included and the clinical diversity of participants, interventions and methods of measurement limit the ability to draw conclusions. It makes little difference whether the exercise is of high or low intensity.” | -/+ | Well-designed randomised controlled trials were recommended |
| (122) | “There was no clear evidence of differences between supervised walking exercise and alternative exercise modes in improving the maximum and pain-free walking distance of patients with intermittent claudication. The results indicate that alternative exercise modes may be useful when supervised walking exercise is not an option for the patient.” | -/+ | Adequately powered trials were recommended |
| (123) | “Yoga has the potential for being included as part of patient-centred stroke rehabilitation.” | -/+ | Conclusions based on two highly biased studies |
| (124) | “There is limited evidence supporting early commencement of weight-bearing and the use of a removable type of immobilisation to allow exercise during the immobilisation period after surgical fixation. Because of the potential increased risk of adverse events, the patient’s ability to comply with the use of a removable type of immobilisation to enable controlled exercise is essential.” | -/+ | / |
| (125) | “This review provides evidence that PRT is an effective intervention for improving physical functioning in older people, including improving strength and the performance of some simple and complex activities.” | + | Adverse events were poorly reported; appropriate dose of the intervention was not established |
| (126) | “We are uncertain of the effects of exercise-based CR compared to control on mortality, morbidity, cardiovascular hospital admissions, adverse events, return to work and health-related quality of life in people with stable angina. Low-quality evidence indicates that exercise-based CR may result in a small increase in exercise capacity compared to usual care." | -/+ | The poor reporting, high risk of bias and small number of trials and participants |
| (127) | “Exercise training may, or may not, confer modest benefit on physical fitness and HRQoL. Limited evidence suggests that exercise training is probably not harmful and probably reduces fatigue.” | -/+ | The findings should be interpreted with caution: low number of studies, and high clinical heterogeneity |
| (128) | “Evidence of very low to moderate quality indicates that motor control exercise (MCE) showed no benefit over spinal manipulative therapy, other forms of exercise or medical treatment in decreasing pain and disability among patients with acute and subacute low back pain. Whether MCE can prevent recurrences of low back pain remains uncertain.” | -/+ | Cautious interpretation is advised |
| (129) | “We could draw no reliable conclusions concerning the use of breathing exercises for children with asthma in clinical practice.” | -/+ | Poor reporting of methodological quality of studies |
| (130) | “School and community-based physical activity interventions as part of an obesity prevention or treatment programme can benefit executive functions of children with obesity or overweight specifically.” | + | The evidence not downgraded for imprecision despite small sample (one trial; 116 participants) |
| (131) | “Evidence from this review indicates that some form of upper limb exercise training when compared to no upper limb training or a sham intervention improves dyspnoea but not HRQoL in people with COPD.” | + | The sample size range: 12 to 43 participants |
| (132) | “There is limited quality evidence that water-based exercise training is safe and improves exercise capacity and quality of life in people with COPD immediately after training. There is limited quality evidence that water-based exercise training offers advantages over land- based exercise training in improving endurance exercise capacity, but we remain uncertain as to whether it leads to better quality of life. Little evidence exists examining the long-term effect of water-based exercise training.” | + | The evidence not downgraded for imprecision despite small sample (one trial; 30 participants) |
| (133) | “Exercise can result in a significant and clinically meaningful improvement in shoulder ROM in Females with breast cancer. In the postoperative period, consideration should be given to early implementation of exercises, although this approach may need to be carefully weighed against the potential for increases in wound drainage volume and duration.” | + | Only one study met all quality criteria; 10 of the 24 trials were considered of adequate methodological quality |
| (134) | “Multi-component behaviour-changing interventions that incorporate diet, physical activity and behaviour change may be beneficial in achieving small, short-term reductions in BMI, BMI z score and weight in children aged 6 to 11 years. The evidence suggests a very low occurrence of adverse events.” | -/+ | Further research in lower income countries and in children from different ethnic groups was recommended |
| (135) | “The low-quality evidence from three small trials with 147 participants does not allow any firm conclusions to be drawn regarding the effectiveness of DMT for depression.” | -/+ | Very low methodological quality of the primary studies |
| (136) | “There is insufficient evidence for reliable conclusions about the effects of exercise on prevention of pre-eclampsia and its complications” | -/+ | High quality trials were recommended with adequate sample size and outcomes (pre-eclampsia related complications) |
| (137) | “The evidence from randomised controlled trials so far does not confirm or refute that water-based exercises after stroke might help to reduce disability after stroke.” | -/+ | All studies scored unclear on sequence generation |
| (138) | “There is insufficient evidence from RCTs to conclude that any one locomotor training strategy improves walking function more than another for people with spinal cord injury.” | - | Small number of trials with mainly unclear risk of bias |
| (139) | “Overall, people after stroke who receive treadmill training, with or without body weight support, are not more likely to improve their ability to walk independently compared with people after stroke not receiving treadmill training, but walking speed and walking endurance may improve slightly in the short term. Specifically, people with stroke who are able to walk (but not people who are dependent in walking at start of treatment) appear to benefit most from this type of intervention with regard to walking speed and walking endurance. This review did not find, however, that improvements in walking speed and endurance may have persisting beneficial effects.” | + | / |
| (140) | “This systematic review indicates that exercise may have beneficial effects on HRQoL and certain HRQoL domains including cancerspecific concerns (e.g. breast cancer), body image/self-esteem, emotional well-being, sexuality, sleep disturbance, social functioning, anxiety, fatigue, and pain at varying follow-up periods.” | + | Further research with longer follow-ups was recommended |
| (141) | “This systematic review indicates that exercise may have beneficial effects at varying follow-up periods on HRQoL and certain HRQoL domains including physical functioning, role function, social functioning, and fatigue. Positive effects of exercise interventions are more pronounced with moderate- or vigorous-intensity versus mild-intensity exercise programs.” | -/+ | Large number of trials and patients |
| (142) | “Exercise, though not appropriate for all in this population, may enhance sleep and contribute to an increased quality of life.” | -/+ | Lack of evidence from well-designed trials |
| (143) | “In people with pulmonary hypertension, exercise-based rehabilitation results in clinically relevant improvements in exercise capacity. Exercise training was not associated with any serious adverse events. Whilst most studies reported improvements in HRQoL, these may not be clinically important.” | + | The total number of participants was 206 |
| (144) | “Moderate-intensity exercise appears to be an important part of controlling weight gain in pregnancy.” | + | Only 20 of 65 studies contributed data for exercise only interventions |
| (145) | “No adverse events were reported, implying that Tai Chi is safe to practise in people with COPD. Evidence of very low to moderate quality suggests better functional capacity and pulmonary function in post-programme data for Tai Chi versus usual care. When Tai Chi in addition to other interventions was compared with other interventions alone, Tai Chi did not show superiority and showed no additional effects on symptoms nor on physical and psychosocial function improvement in people with COPD.” | -/+ | The number of participants ranged from 10 to 206 |
| (146) | “We found some evidence that biofeedback and electrical stimulation may enhance the outcome of treatment compared to electrical stimulation alone or exercises alone. Exercises appear to be less effective than an implanted sacral nerve stimulator” | -/+ | Methodological weaknesses prevent any definitive judgment |
| (147) | “Progressive resistive exercise or a combination of progressive resistive exercise and aerobic exercise appear to be safe and may be beneficial for adults living with HIV/AIDS.” | + | The findings are limited by the small number of studies, small sample sizes and variable participant withdrawal rates |
| (148) | “Aerobic exercise appears to be safe and may be beneficial for adults living with HIV. These findings are limited by the small sample sizes and large withdrawal rates described in the studies.” | + | These findings are limited by methodological weaknesses and high withdrawal rates |
| (149) | “When we pooled results from five studies, we found low-quality evidence showing small beneficial effects of exercise on hand pain, function and finger joint stiffness.” | -/+ | Small effect sizes, clinical relevance questionable |
| (150) | “The best available data show that a combination of manual therapy and exercise may not be as effective as glucocorticoid injection in the short-term. It is unclear whether a combination of manual therapy, exercise and electrotherapy is an effective adjunct to glucocorticoid injection or oral NSAID. Following arthrographic joint distension with glucocorticoid and saline, manual therapy and exercise may confer effects similar to those of sham ultrasound in terms of overall pain, function and quality of life, but may provide greater patient- reported treatment success and active range of motion.” | -/+ | The overall quality of evidence for most comparisons was low; complex interventions were evaluated |
| (151) | “Despite identifying 60 eligible trials, only one trial compared a combination of manual therapy and exercise reflective of common current practice to placebo. We judged it to be of high quality and found no clinically important differences between groups in any outcome. Effects of manual therapy and exercise may be similar to those of glucocorticoid injection and arthroscopic subacromial decompression, but this is based on low quality evidence. Adverse events associated with manual therapy and exercise are relatively more frequent than placebo but mild in nature.” | -/+ | Only 10 of 60 trials pertained to exercise |
| (152) | “There is limited and very low-quality evidence of benefit for all of a diverse collection of exercise and mobilisation interventions for carpal tunnel syndrome” | -/+ | More research was suggested |
| (153) | “No reliable conclusions can be drawn at present regarding the efficacy of yoga as a treatment for epilepsy. The complexity of the yoga intervention is similar to other forms of complementary and alternative treatments. Yoga may be an add-on to antiepileptic drugs (AEDs) at the present time and cannot be used as the sole method of intervention.” | -/+ | Very small number of studies and participants |
| (154) | “We found no evidence that undertaking therapeutic exercises before, during and/or immediately after HNC treatment leads to improvement in oral swallowing. This absence of evidence may be due to the small participant numbers in trials, resulting in insufficient power to detect any difference. Data from the identified trials could not be combined due to differences in the choice of primary outcomes and in the measurement tools used to assess them, and the differing baseline and endpoints across studies.” | **-** | Further studies with validated assessment tools to measure outcomes were recommended |
| (155) | “Conclusions about the efficacy of physical exercise training in cystic fibrosis (CF) are limited by the small size, duration and incomplete reporting of most of the studies included in this review. However, there is limited evidence that physical exercise training is beneficial.” | **-/+** | Uncertainty about the estimates and further research were recommended |
| (156) | “We found very low- to low-quality evidence for no important clinical benefit of high-intensity compared to low-intensity exercise programs in improving pain and physical function in the short term.“ | - | The small number of studies and participants included in some analyses reduced the robustness and precision of the findings |
| (157) | “There is no evidence to support - or refute - the use of dance therapy. Those with schizophrenia may wish to be involved in future research to help to resolve this lack of evidence.” | -/+ | Only one trial was included; caution interpretation advised |
| (158) | “The results of this review suggest that exercise therapy, whether similar to that recommended for the healthy population or modified to simply maintain function, does have efficacy in MS. There was no evidence described of deleterious effects of exercise therapy for patients with MS and the effect of type of MS remains unclear. Based on these results, it seems reasonable to promote exercise therapy to patients with MS not experiencing an exacerbation.” | + | Small total sample seize |
| (159) | “Due to few randomised patients and outcomes, we could not evaluate the real impact of exercise-based cardiac rehabilitation on mortality or serious adverse events. The evidence showed no clinically relevant effect on health-related quality of life. Pooled data showed a positive effect on the surrogate outcome of physical exercise capacity, but due to the low number of patients and the moderate to very low-quality of the underpinning evidence, we could not be certain of the magnitude of the effect. Future high-quality randomised trials are needed to assess the benefits and harms of exercise-based cardiac rehabilitation for adults with atrial fibrillation on patient-relevant outcomes.” | +/- | Only a few trials with a total of less than 500 participants |
| (160) | “There is lack of high-quality evidence to recommend the use of scoliosis-specific exercises for adolescent idiopathic scoliosis. One very low-quality study suggested that these exercises may be more effective than electrostimulation, traction and postural training to avoid scoliosis progression, but better quality research needs to be conducted before the use of scoliosis-specific exercises can be recommended in clinical practice.” | -/+ | Majority of the outcomes not reported |
| (161) | “There is low- to very low-quality evidence that aerobic exercise results in a small improvement in gross motor function but not aerobic fitness, and that resistance training results in a small improvement in muscle strength but not activity or participation in people with CP. Exercise appears to be safe for people with CP from the limited evidence available.” | -/+ | All of the studies had small sample sizes |
| (162) | “There is very low to moderate quality evidence that MCE has a clinically important effect compared with a minimal intervention for chronic low back pain. There is very low to low quality evidence that MCE has a clinically important effect compared with exercise plus EPA. There is moderate to high quality evidence that MCE provides similar outcomes to manual therapies and low to moderate quality evidence that it provides similar outcomes to other forms of exercises. Given the evidence that MCE is not superior to other forms of exercise, the choice of exercise for chronic LBP should probably depend on patient or therapist preferences, therapist training, costs and safety” | **+/-** | Limited generalizability |
| (163) | **“**Cardiorespiratory training and mixed training during or after usual stroke care is effective in increasing walking speed and walking capacity in stroke survivors. It is likely that improvements in fitness, mobility, and physical function outcomes are associated with ’task related’ training. “ | **+** | There were some consistent findings |
| (164) | “Some evidence from eleven small randomized trials of moderate methodological and reporting quality indicates that physical activity programs might promote moderate short-term growth and bone mineralization in preterm infants. The clinical importance of these findings is questionable, given the small effect size and the low baseline risk of poor bone mineralization and growth in study participants. Available data are inadequate to permit assessment of harm or long-term effects of physical activity programs.” | **+/-** | All trials were small, range: 16-50 |
| (165) | “The evidence to date is entirely limited to small studies with small sample sizes, short-term follow-up, and at high risk of methodological bias, which makes it difficult to derive any conclusions on the efficacy or safety of aerobic or resistance exercise among individuals with increased risk of cardiovascular disease or with two or more coexisting risk factors. We cannot, therefore, reach any conclusion, neither in favor nor against this intervention” | **+/-** | Future studies with larger sample sizes were recommended |
| (166) | “This review suggests that exercise is an effective weight loss intervention, particularly when combined with dietary interventions. Exercise is also an effective intervention for improving a range of secondary outcomes even when weight loss does not occur. While this review did not show any long-term morbidity and mortality benefits associated with exercise, exercise was shown to positively impact the intermediate outcomes that are commonly associated with cardiovascular disease.” | **+** | Data for exercise versus no treatment control were analysed here |
| (167) | “Moderate-quality evidence suggests reduced risks of GDM and caesarean section with combined diet and exercise interventions during pregnancy as well as reductions in gestational weight gain, compared with standard care. There were no clear differences in hypertensive disorders of pregnancy, perinatal mortality, large-for-gestational age, perineal trauma, neonatal hypoglycaemia, and childhood adiposity (moderate- to very low-quality evidence).” | **+/-** | Evidence has limited ability to inform practice |
| (168) | “Our findings suggest that exercise-based rehabilitation for adults after heart valve surgery, compared with no exercise, may improve exercise capacity. Due to a lack of evidence, we cannot evaluate the impact on other outcomes. Further high-quality randomised clinical trials are needed in order to assess the impact of exercise-based rehabilitation on patient-relevant outcomes, including mortality and quality of life.” | **+/-** | Two small trials; cautions conclusions and limited generalizability |
| (169) | “There is no conclusive evidence in this review to support or refute inspiratory muscle training for asthma.” | -/+ | Small number of participants (<100 participants) for all outcomes |
| (170) | “We found insufficient evidence to determine if overground physical therapy gait training benefits gait function in patients with chronic stroke, though limited evidence suggests small benefits for unidimensional variables such as gait speed or 6MWT.” | -/+ | Well-designed trials of sufficient size and quality were suggested |
| (171) | “Most exercise interventions produced improvement in one or more of the measured outcomes including pain, range of motion, strength and walking tolerance. Hydrotherapy may be more effective than land exercises for pain relief in adults. Functional exercises such as treadmill walking and partial weight bearing exercises seem to be more effective than static or short arc exercises for improving muscle strength. These findings are consistent with the many non-controlled intervention reports in the haemophilia literature. No adverse effects were reported as a result of any of the interventions.” | -/+ | Results should be interpreted with caution due to the quality of the evidence |
| (172) | “Overall, based on ’silver-level’ evidence (www.cochranemsk.org) there was no clinically important or statistically significant evidence that exercise therapy can improve functional ability, quality of life, aerobic capacity or pain. The low number of available RCTs limits the generalisability. The included and excluded studies were all consistent about the adverse effects of exercise therapy; no short- term detrimental effects of exercise therapy were found in any study. Both included and excluded studies showed that exercise does not exacerbate arthritis. The large heterogeneity in outcome measures, as seen in this review, emphasises the need for a standardised assessment or a core set of functional and physical outcome measurements suited for health research to generate evidence about the possible benefits of exercise therapy for patients with JIA. Although the short-term effects look promising, the long-term effect of exercise therapy remains unclear.” | -/+ | A total of 212 participants |
| (173) | “This updated Cochrane review supports the conclusions of the previous version of this review that, compared with no exercise control, exercise-based rehabilitation does not increase or decrease the risk of all-cause mortality in the short term (up to 12-months’ follow-up) but reduces the risk of hospital admissions and confers important improvements in health-related quality of life. This update provides further evidence that exercise training may reduce mortality in the longer term and that the benefits of exercise training on appear to be consistent across participant characteristics including age, gender and HF severity.” | + | Poor reporting was highlighted |
| (174) | “The meta-analysis shows that exercise significantly improves glycaemic control and reduces visceral adipose tissue and plasma triglycerides, but not plasma cholesterol, in people with type 2 diabetes, even without weight loss.” | + | Long term intervention studies were suggested beneficial |
| (175) | “Only two of the 20 trials offered evidence for exercise aiding smoking cessation in the long term. All the other trials were too small to reliably exclude an effect of intervention, or included an exercise intervention which may not have been sufficiently intense to achieve the desired level of exercise.” | -/+ | Some trials were small and had numerous methodological limitations |
| (176) | “The current findings indicate that treadmill intervention may accelerate the development of independent walking in children with Down syndrome and may accelerate motor skill attainment in children with cerebral palsy and general developmental delay.” | -/+ | Optimal dosage of intervention not established |
| (177) | “This review has found very low quality but consistent evidence that exercise therapy for PFPS may result in clinically important reduction in pain and improvement in functional ability, as well as enhancing long-term recovery.” | -/+ | Uncertainty about reliability of the results |
| (178) | “There is very low- to moderate-quality evidence that CME may be a valuable intervention to augment the pallet of therapeutic options for stroke rehabilitation.” | + | Results should be interpreted with caution |
| (179) | “Moderate-intensity strength training in myotonic dystrophy and FSHD and aerobic exercise training in dermatomyositis and polymyositis and myotonic dystrophy type I appear to do no harm, but there is insufficient evidence to conclude that they offer benefit. In mitochondrial myopathy, aerobic exercise combined with strength training appears to be safe and may be effective in increasing submaximal endurance capacity.” | -/+ | Only five trials with 170 participants |
| (180) | “There is inadequate evidence to evaluate the effect of exercise on functional ability in people with peripheral neuropathy. The results suggest that progressive resisted exercise may improve muscle strength in affected muscles.” | -/+ | Conclusions based on very small number of methodologically weak trials |
| (181) | “There is low- to moderate-certainty evidence that yoga compared to no exercise controls results in small to moderate improvements in back-related function at three and six months. Yoga may also be slightly more effective for pain at three and six months, however the effect size did not meet predefined levels of minimum clinical importance. It is uncertain whether there is any difference between yoga and other exercise for back-related function or pain, or whether yoga added to exercise is more effective than exercise alone. Yoga is associated with more adverse events than no exercise controls, but may have the same risk of adverse events as other back-focused exercise. Yoga is not associated with serious adverse events.” | + | 12 trials with 1080 participants; further research was recommended |
| (182) | “There is a paucity of evidence for exercise training to reduce fall rates in people living with and beyond cancer. Exercise training may improve strength, flexibility and balance for people in this population, but the evidence is very low quality.” | -/+ | / |
| (183) | “It is uncertain whether exercise improves hand function or pain in the short term. It probably slightly improves function but has little or no difference on pain in the medium and long term. It is uncertain whether exercise improves grip and pinch strength in the short term, and probably has little or no difference in the medium and long term. The ACR50 response is unknown. People who received exercise with adherence strategies were probably more adherent in the medium term than who did not receive exercise, but with little or no difference in the long term. Hand exercise probably does not lead to adverse events.” | -/+ | Lack of blinding of participants to their allocated treatment and measurements, methods of allocation, and small study sizes |
| (184) | “The evidence is currently inadequate to assess the safety and efficacy of exercise-based CR for people with implantable VADs compared with usual care. The amount of RCT evidence was very limited and of very low quality. In addition, the training duration was very short term, that is from six to eight weeks.” | -/+ | Future trials should include mortality and re-hospitalisation, patient-related outcomes, and cost-effectiveness data |
| (185) | “We did not find any high quality evidence for any of the treatment comparisons, outcomes or follow-up periods investigated. However, there is low to moderate quality evidence that Pilates is more effective than minimal intervention for pain and disability. When Pilates was compared with other exercises we found a small effect for function at intermediate-term follow-up. Thus, while there is some evidence for the effectiveness of Pilates for low back pain, there is no conclusive evidence that it is superior to other forms of exercises.” | + | 10 trials with 510 participants only |
| (186) | “We found moderate-quality evidence that yoga probably leads to small improvements in quality of life and symptoms in people with asthma. There is more uncertainty about potential adverse effects of yoga and its impact on lung function and medication usage.” | + | More, high-quality studies were recommended |
| (187) | “We found no evidence in the available data from RCTs that aerobic physical activities, including those which successfully improve cardiorespiratory fitness, have any cognitive benefit in cognitively healthy older adults.” | - | Lack of blinding and high attrition rates were of greatest concern |
| (188) | “Comparisons between the higher and lower training intensity were limited due to the small number of included studies and participants. Consequently, there are insufficient data to draw any conclusions on exercise capacity, symptoms and HRQoL for this comparison. For comparisons between continuous and interval training, both appear to be equally effective in improving exercise capacity, symptoms and HRQoL.” | -/+ | / |
| **Total** | / | (-/+) = 91  (+) = 49  (-) = 10 | / |

**Table 3 Footnote:**

ADL: activities in daily life, BMI: body mass index, CR: exercise training alone or in combination with psychosocial or educational interventions, CIMT: constraint-induced movement therapy, CKD: chronic kidney disease, COPD: Chronic obstructive pulmonary disease, CVD: cardiovascular disease, CVI: chronic venous insufficiency, FM: Fibromyalgia, HBET: homebased exercise therapy, HDL: high-density lipoprotein cholesterol, HRQL: health-related quality of life, HT: hormone therapy, ICU: intensive care unit, IGT: impaired glucose tolerance, LBP: low-back pain, LDL: low-density lipoprotein cholesterol, MI: myocardial infarction, MWD: maximal treadmill walking distance, NSCLC: non-small cell lung cancer, OA: osteoarthritis, QoL: quality of life, PD: Parkinson’s disease, PFWD: pain-free treadmill walking distance, RA: rheumatoid arthritis, RCT: randomised controlled trial, SET: supervised exercise therapy, T2DM: type 2 diabetes mellitus, TBI: traumatic brain injury, WA: walking advice, WBV: whole body vibration. + positive direction; - negative direction; -/+ equivocal direction.

**Supplementary Table 4. AEs reported in Cochrane systematic reviews**

| **Reference** | **Total number of trials** | **Adverse events reported (y/n)** | **Number of AE** | **Severity of AE** | **Description** | **Comment** |
| --- | --- | --- | --- | --- | --- | --- |
| (39) | 4 | n | / | / | / | No data were reported on adverse effects |
| (40) | 44 | y | / | / | Adverse events were experienced in 25% of participants in the diet plus aerobic exercise group; 19% in the diet plus resistance training group; 21% in the diet plus aerobic plus resistance training group and 24% in the diet-only group. | Only 5 trials reported  adverse events and of  these details were provided in only 1 showing no substantial differences between intervention and comparator groups |
| (41) | 12 | y | 0 | / | One trial found no adverse effect of diet plus exercise on breastfeeding performance | / |
| (42) | 10 | y | 1 | Severe | A myocardial infarction resulting in heart failure was reported in the comparator group; and none in the intervention group | Six studies reported no adverse events occurred. Two studies did not report if any |
| (43) | 63 | n | / | / | / | / |
| (44) | 3 | y | 1 | / | No undesired event occurred during a six-month exercise training program | / |
| (45) | 2 | n | / | / | / | / |
| (46) | 6 | n | / | / | / | / |
| (47) | 13 | y | 11 | Mild-moderate | Mild joint discomfort, lumbar pain, and cramps in the calf or foot, low-back pain, heart problem, dizziness | Total number of events: 112 (Exercise), versus 89 (Control). RR=1.25 (95% CI 0.98 to  1.60) |
| (48) | 8 | n | / | / | / | “Based on limited data there were no adverse effects on asthma control or occurrence of exacerbations.” |
| (49) | 9 | y | 31 serious | Mild-severe | Bleeding, infection, fever, hyponatraemia, pneumonia, hyperglycaemia, deep vein thrombosis | AEs (RR 7.23; 95% CI 0.38 to 137.05; P = 0.19, 1 study, low quality of evidence); serious AEs (RR 1.44 (95% CI 0.96 to 2.18; 3 studies, low certainty evidence)) |
| (50) | 13 | y | 3 | Mild-moderate | “unable to exercise after an injury’’ or ‘‘one participant assigned to the short bout exercise withdrew after developing a metatarsal stress fracture” | Unclear whether the injury was related to exercise |
| (51) | 4 | y | 1 | Mild | Acute pain in the leg | / |
| (52) | 16 | y | 73 | / | Muscle pain, first-degree burns, urinary infection, tinea pedis | Only five studies reported adverse effects |
| (53) | 6 | y | 0 | / | One study reported that none of the children experienced any negative effects from the exercises | The other studies did not report on adverse events |
| (54) | 3 | n | / | / | / | No adverse effects were reported |
| (55) | 6 | y | 0 | / | Only one study reported that none adverse effects occurred | 85 participants, moderate certainty evidence |
| (56) | 8 | y | 0 | 0 | Only one study reported that none adverse effects occurred | 94 participants, moderate certainty evidence |
| (57) | 3 | y | / | / | / | No study reported on adverse effects |
| (58) | 11 | n | / | / | / | / |
| (59) | 34 | y | 5 | Mild | Pain, stiffness and fatigue, worsening of symptoms, feelings of soreness and tiredness, metatarsal stress fracture, ischialgia | Some studies did not report any adverse effects |
| (60) | 5 | y | / | / | None serious occurred | “women with fibromyalgia could safely perform resistance training (no serious adverse effects were reported)” |
| (61) | 95 | y | 30 | 1 | Death due to a ruptured abdominal aortic aneurysm one week after the follow-up test (association could not definitely be ruled out) | Data from 4 studies with 1032 participants, very low certainty evidence |
| (62) | 21 | n | / | / | / | “Physical training was well tolerated with no adverse effects reported” |
| (63) | 3 | y | 2 | Mild | Nausea, increased pain as a result of soft-tissue injury to the scapular region | No serious adverse events were reported |
| (64) | 5 | n | / | / | / | / |
| (65) | 3 | n | / | / | / | / |
| (66) | 4 | n | / | / | / | / |
| (67) | 9 | n | / | / | / | Adverse events were not mentioned in any of the studies |
| (68) | 7 | y | 1 | / | / | One trial reported that no adverse events occurred |
| (69) | 6 | y | 5 | Mild to serious | A minor  musculoskeletal injury; overall mortality rates range: 4.8%-18.8% | Overall four deaths; no between group differences |
| (70) | 39 | y | / | Mild | Increased severity of depressive symptoms (n=1), chest pain (n=1) and joint pain/swelling (n=1) (among others) | Seven trials reported no between groups differences in adverse events |
| (71) | 42 | y | 6 | / | The intervention “appeared to have no adverse effects” | Rates of adverse events did not differ between the groups |
| (72) | 24 | y | 40 | Mild | / | Under yoga versus no therapy |
| (73) | 56 | n | / | / | / | / |
| (74) | 2 | y | 0 | Mild | Increased muscle cramping, muscle soreness or fatigue | None reported |
| (75) | 2 | y | 0 | / | / | None reported |
| (76) | 5 | y | See description | / | 17% for exercise and 18% for usual care | No between group differences |
| (77) | 9 | y | / | / | / | No incidence of musculoskeletal injuries as a result of exercise intervention (2 trials, n = 396) |
| (78) | 44 | n | / | / | / | / |
| (79) | 4 | y | 27 | Mild to moderate | Oxygen desaturation, dislodgement of the radial catheter; instability; postoperative pulmonary complications; asymptomatic bradycardia | AEs were unrelated to intervention |
| (80) | 23 | n | / | / | / | / |
| (81) | 11 | y | / | / | / | None related to exercise were reported |
| (82) | 1 | n | / | / | / | / |
| (83) | 17 | y | / | / | / | None of these trials reported any serious adverse events |
| (84) | 54 | y | 42 | Mild | Increased knee or low back pain (8 studies) | No study reported a serious adverse event |
| (85) | 10 | y | 7 | Mild | Increased back pain, increased blood pressure | No serious adverse events were reported |
| (86) | 13 | y | / | / | No adverse effects related to the intervention occurred | One study reported that one patient died from a myocardial infarct in the control group |
| (87) | 32 | y | 59 | Mild | Knee discomfort; unexplained leg pain; musculoskeletal pain or injury; muscle soreness; shoulder tendonitis; hip pain, sciatica (n = 16), arm discomfort (n = 4), knee discomfort (n = 10), ankle discomfort (n = 3), and foot discomfort (n = 8) | Seven trials reported a very small number of adverse events |
| (88) | 7 | y | 35 | Mild to moderate | Fracture of costal cartilage; rib fracture; metatarsal Fracture | Only AEs related to exercise are reported here |
| (89) | 159 | y | 28 | Mild to moderate | Musculoskeletal complaints or injuries | RR 3.6, 95% CI 1.5-8.0 for strength/ resistance training versus control |
| (90) | 3 | y | See description | / | MD 0.50, 95% CI -0.44 to 1.44 | Between yoga and exercise groups |
| (91) | 11 | n | / | / | / | / |
| (92) | 6 | y | / | / | None occurred | Not estimable for exercise |
| (93) | 3 | y | 1 | Mild | Skin sensitivity | RR 2.65, 95% CI 0.12 to 60.21 |
| (94) | 27 | y | 16 | Mild to moderate | Increased neck or headache, increased radicular pain, severe thoracic pain | Minor and short-lasting adverse events were reported in 25% of the studies |
| (95) | 21 | y | 13 | Severe | Death | No deaths were related to exercise therapy |
| (96) | 4 | y | / | Mild | Knee pain, leg cramps and ankle soreness | Reported by approx.. 1/3 of the patients in two studies |
| (97) | 5 | n | / | / | / | / |
| (98) | 11 | n | / | / | / | Adverse events, were not reported in any of the included studies |
| (99) | 13 | y | / | / | / | “None of the included trials reported on adverse events” |
| (100) | 11 | y | / | / | / | None of the included studies provided information on adverse events |
| (101) | 8 | y | / | / | / | No adverse events occurred |
| (102) | 61 | y | / | Mild | Increased low back pain and muscle soreness | Reported in 12 studies |
| (103) | 21 | y | / | / | / | No AEs occurred (reported in one trial) |
| (104) | 45 | y | / | / | / | One trial reported that none occurred |
| (105) | 45 | y | 1 | Moderate | Partial tearing of musculus supraspinatus | / |
| (106) | 12 | y | 12 | Serious | Deaths | RR 1.12, 95% CI 0.50 to 2.50 |
| (107) | 12 | n | / | / | / | / |
| (108) | 19 | n | / | / | / | / |
| (109) | 16 | y | / | / | / | “No significant adverse effects were reported.” |
| (110) | 94 | y | / | / | / | “The majority of studies either did not report on any adverse events (n = 55) or reported that there were no adverse events (n = 30)” |
| (111) | 43 | y | 75 | Mild | Muscle soreness, joint pain, headache and itching | Fractures and falls were reported as adverse events in some studies |
| (112) | 8 | y | / | / | / | None occurred |
| (113) | 21 | y | / | / | / | No information on adverse effects |
| (114) | 1 | n | / | / | / | / |
| (115) | 12 | y | / | / | / | Eight studies reported that no adverse events occurred |
| (116) | 30 | n | / | / | / | / |
| (117) | 14 | n | / | / | / | / |
| (118) | 63 | y | 50 | Mild to moderate | Plantar fasciitis, discomfort, stiffness, chronic back and shoulder problems, tendonitis, pelvis stress fracture, chest pain, asthma episode, hypoglycemia episode, broken hip, musculoskeletal injuries | Often AEs were not attributed to the interventions |
| (119) | 32 | y | 2 | / | Gastroenteritis | Not considered to be AE of pentoxifylline according to the trialists |
| (120) | 8 | y | 2 | Serious | Deterioration in mobility and self-care, worse symptoms and function; suicidal thoughts, a worsened depression; self-harm | RR 0.99, 95% CI 0.14 to 7.1 (1 study with 160 participants) |
| (121) | 16 | n | / | / | / | / |
| (122) | 5 | n | / | / | / | / |
| (123) | 2 | y | / | / | / | None reported |
| (124) | 7 | y | 23 | Moderate to serious | Additional surgery on the fractured ankle | Combined estimate (intervention + control) under: exercise post-immobilisation after surgical fixation |
| (125) | 121 | y | 43 | Mild | Joint pain and muscle soreness | Two deaths reported – causality not established. 53 trials failed to report any AEs. |
| (126) | 7 | y | / | / | / | Only one study reported that ‘no adverse events’ occurred |
| (127) | 11 | y | 1 | / | One patient with brain tumor experienced a grade 3 seizure after exercise training | Seven studies reported that no adverse events occurred |
| (128) | 3 | y | / | / | / | Not reported in any of the trials |
| (129) | 3 | y | / | / | / | No studies reported serious adverse events |
| (130) | 4 | n | / | / | / | No data on AEs available |
| (131) | 15 | n | / | / | / | / |
| (132) | 5 | y | 1 | Mild | One accidental skin tear | No adverse events reported in the second study |
| (133) | 24 | y | / | / | / | / |
| (134) | 70 | y | 4 | 31 trials reported on serious adverse events | Seasonal influenza that required hospitalisation (N = 3), hip surgery due to a chronic condition (N = 1), an ankle injury (N = 1), diagnosis of type 1 diabetes (N = 1), a blood clot (N = 1) and observation after a fall (N=1) | None of these were seen as related to the study |
| (135) | 3 | n | / | / | / | None reported |
| (136) | 2 | n | / | / | / | / |
| (137) | 4 | n | / | / | / | No information provided |
| (138) | 5 | y | See comments | / | / | 193 versus 116; RD 0.03, 95% CI -0.02 to 0.07 |
| (139) | 56 | y | See comments | / | Falls, injuries, cardiovascular events | 835 versus 669; RD 0.02, 95% CI -0.01 to 0.05 |
| (140) | 40 | y | / | / | Foot fracture, back problems, knee, hip, hip pain, sciatica, discomfort, heart palpitation, soft tissue injury, acute cholecystitis, musuloskeletal issues | Not or none reported in the majority of trials |
| (141) | 56 | y | / | Mild to severe | Dizziness, weakness, mild diarrhea, hip, knee, back problems, heart palpitations, death, myocardial infarctions, syncope, chest pain, anemia, discomfort, dyspnea | As above |
| (142) | 1 | n | / | / | / | / |
| (143) | 5 | y | 1 | / | Lightheadedness | Reported in one study |
| (144) | 65 | n | / | / | / | / |
| (145) | 12 | y | / | / | / | None occurred |
| (146) | 21 | n | / | / | / | / |
| (147) | 7 | y | 9 | Mild to severe | Death (n=4), breast enlargement, acne, testicular shrinkage | No adverse events were attributed to exercise |
| (148) | 14 | y | 4 | Mild to severe | Death (n=2), exacerbation of asthma, chest pain, hospitalization | None of those adverse events were attributed to exercise |
| (149) | 7 | y | 4 | Mild | Pain, inflammation, swelling in all fingers | RR 4.55, 95% CI 0.53 to 39.31 |
| (150) | 32 | y | 32 | Mild | Pain after treatment, facial flushing, irregular menstrual bleeding, fever and skin irritation | RR 1.07, 95% CI 0.76-1.49 for passive mobilisation and supervised exercise versus glucocorticoid injection |
| (151) | 60 | y | 17 | Mild | Short-term pain | RR 3.77, 95% CI 1.49 to 9.54 |
| (152) | 16 | n | / | / | / | No adverse effects were reported |
| (153) | 2 | n | / | / | / | / |
| (154) | 6 | y | / | / | Oral pain, throat discomfort and/or fatigue, mouth/throat pain, coughing and gagging | “There were no adverse events reported that were directly attributable to the intervention” |
| (155) | 15 | y | 2 | Mild | Ankle injury, haemoptysis | / |
| (156) | 6 | y | 7 | Mild | Knee, back pain | No severe adverse events were observed |
| (157) | 1 | y | 1 | / | / | No between groups differences |
| (158) | 9 | / | / | / | / | / |
| (159) | 6 | y | 3 | Severe | Retinal embolism, death, hospitalisation | / |
| (160) | 2 | n | / | / | / | / |
| (161) | 29 | y | 19 | Mild | Leg pain, soreness, muscle cramping or mild pain | AEs were “potentially related to the study” |
| (162) | 29 | y | / | Mild | / | 2 trials reported mild adverse events |
| (163) | 58 | y | 84 | See comment | 71 falls, 10 cerebrovascular events, 3 cardiovascular events | “There was no evidence of any serious adverse events arising from  training in people who participated in physical fitness training programmes“ |
| (164) | 11 | n | / | / | / | / |
| (165) | 4 | n | / | / | / | / |
| (166) | 43 | n | / | / | / | “No data were identified on adverse events [..]” |
| (167) | 23 | n | / | / | / | / |
| (168) | 2 | y | 11 | Severe | 2 heart arrhythmias,  1 sudden death,  1 brain stem death,  1 hematoma in abdominal muscle,  1 angina pectoris | RR 1.15, 95% CI 0.37 to 3.62; no difference between the groups |
| (169) | 5 | n | / | / | / | / |
| (170) | 9 | y | 6 | Mild to severe | Deaths (n=4 in the control group), fatigue, groin pain, falls | OR 0.18, 95% CI 0.02, 1.55 (for deaths); and 3.00, 95% CI 0.30, 29.64 for other adverse effects |
| (171) | 8 | n | / | / | / | “There were no adverse effects measured or reported” |
| (172) | 3 | n | / | / | / | “No short-term effects have been reported” |
| (173) | 33 | n | / | / | / | / |
| (174) | 14 | y | 1 | Severe | Cerebrovascular accident (n=1) in the control group | / |
| (175) | 20 | n | / | / | / | / |
| (176) | 7 | n | / | / | / | / |
| (177) | 31 | y | See comment | See comment | See comment | Not actively sought in the majority of trials |
| (178) | 9 | y | See comment | Mild | Falls | MD 0.04, 95%CI -0.10 to 0.18; very low quality of evidence |
| (179) | 5 | y | / | Mild | Muscle soreness and transient strength reduction, | Atrial arrhythmia (n=1) not related to exercise |
| (180) | 3 | y | 1 | Mild | Pain | / |
| (181) | 12 | y | 30 | Mild | Pain | RD 0.05, 95% CI 0.02, 0.08 |
| (182) | 11 | y | - | Mild | Temporary muscle soreness, fatigue | / |
| (183) | 7 | y | / | / | / | One study (n = 246) indicated no adverse events with exercising |
| (184) | 2 | y | 2 | Severe | Driveline infection | / |
| (185) | 10 | y | 2 | Mild | Shoulder and knee pain | / |
| (186) | 15 | y | 1 | Mild | Mild dyspnea | / |
| (187) | 12 | n | / | / | / | No trial reported on adverse effects |
| (188) | 11 | n | / | / | / | / |

**Table 4** Footnote: / = inestimable; n = no; y = yes

**Supplementary Table 5.** **Summary of withdrawals/non-adherence**

| **Reference** | **Total number of trials reporting**  **withdrawals/non-adherence** | **Number or %**  **withdrawn/non-adhering –**  **intervention group** | **Number or %**  **withdrawn/non-adhering –**  **control group** | **Estimate or comment** |
| --- | --- | --- | --- | --- |
| (39) | / | / | / | / |
| (40) | 40 | Range: 34% to 100%^a^ | Range: 38% to 100%^a^ | / |
| (41) | 3 | Range: 40.4 to 66% | / | / |
| (42) | / | 10.0% | 10.6% | / |
| (43) | 1 | / | / | Exercise adherence in the first year was 68% (39% to 92%), over the next 5 years 33%  (3% to 89%) in one trial |
| (44) | 3 | / | / | In one trial, 7% of randomised participants withdrew |
| (45) | / | / | / | / |
| (46) | 2 | 68% (adherence rate) | 36% (adherence rate) | At one-year follow-up 75.1-78.7% of the home-based participants were adhering to their exercise program compared with 52.6% of the centre-based (p<0.0005) |
| (47) | 13 | 18 per 100 (14 to 23) | 15 per 100 | Minor inconsistency in reporting of withdrawals: RR 1.25 (95% CI 0.98 to 1.60) in the tables vs 1.21 (95% CI 0.94 to 1.56) in the text |
| (48) | 8 | / | / | Out of 262, 42 participants (16%) withdrew |
| (49) | 1 | / | / | One participant with knee pain withdrew from the exercise programme, and the other two participants proceeded with a modified exercise programme |
| (50) | 9 | 20 out of 100 at 6-24 weeks follow-up | 17 out of 100 at 6-24 weeks follow-up | Withdrawals defined as all-cause attrition: RR 1.25 (95% CI 0.89 to 1.77, 8 studies, moderate certainty evidence) |
| (51) | 1 | 14 per 100 (3 of 21) | 10 per 100 (2 of 20) | RR 1.43 (95% CI 0.27 to 7.67) |
| (52) | 8 | 38/ 252 (15.1%) | 30/ 232 (12.9%) | RR: 1.13 (95% CI 0.73 to 1.77, eight studies, low certainty evidence) |
| (53) | 6 | / | / | In one study, one child withdrew after 3 months |
| (54) | 1 | / | / | Eight women withdrew after randomisation, nine patients dropped out from one study |
| (55) | 2 | / | / | Withdrawals reported in two trials: range 26-27.5% |
| (56) | 4 | / | / | In one study “…expected adherence to the yoga intervention was > 70%, […]” |
| (57) | / | / | / | / |
| (58) | 1 | 10 out of 10 | 9 out of 9 | RR 1.00, 95% CI 0.83 to 1.21, one study, N=19 |
| (59) | / | / | / | Poorly reported |
| (60) | 3 | 134 per 1000 | 39 per 1000 | Based on 3 studies; and low certainty evidence |
| (61) | 5 | / | / | In one study, there was an pprox... 30%withdrawal due to death and discharge |
| (62) | 1 | / | / | Five children withdrew due to logistic reasons |
| (63) | 2 | Range: 93-95% | 87% | / |
| (64) | / | / | / | Not measured |
| (65) | 1 | 7 | / | Two withdrew for not wanting to receive intervention; five withdrew consent for other reasons |
| (66) | / | / | / | / |
| (67) | / | / | / | / |
| (68) | / | / | / | / |
| (69) | 1 | / | / | Mean = 12 (out of 16 supervised  sessions) and mean = 6 (out of 8 unsupervised sessions) |
| (70) | 29 | 865 per 1000 (839 to 900) | 865 per 1000 | Acceptability of treatment: RR 1.0 95% CI: 0.97 to 1.04, moderate certainty evidence |
| (71) | 16 | / | / | Dropout rates of 4% to 23%, including losses for non-medical reasons and difficulties in performing activities of daily living |
| (72) | / | / | / | Dropouts range: 0-59% |
| (73) | 16 | / | / | Adherence varied between 61% and 98.4% |
| (74) | 2 | Range: 15.4-28.6% | Range: 0-15.4% | Reasons were: respiratory and mobility problems, rapid disease deterioration or death, long distances to travel, fatigue, and lack of interest |
| (75) | 2 | / | / | Drop outs (n=9) |
| (76) | / | Range: 3-44% | 5% | “Future trials should measure and report adherence in greater detail” |
| (77) | 2 | Range: 6.3-18% | / | / |
| (78) | / | / | / | / |
| (79) | 3 | 15/26^a^ | 5/24^a^ | At 6 months post-hospital discharge |
| (80) | / | / | / | / |
| (81) | / | / | / | / |
| (82) | 1 | / | / | “One person dropped out of the intervention group before attending any session and was not included in the data analysis.” |
| (83) | 13 | Range: 1.2%-77% | / | / |
| (84) | 44 | 14%  137 per 1000 | 15%  153 per 1000 | OR 0.93, 95% CI 0.75 to 1.15; high quality evidence |
| (85) | 7 | 59 per 1000 | 34 per 1000 | OR 1.77, 95% CI 0.86 to 3.65; moderate quality evidence |
| (86) | 13 | (n=11) | (n=6) | / |
| (87) | 28 | / | / | “In the remaining 22 studies, adherence to the exercise intervention was so low that we judged it to cause a high risk of bias.” |
| (88) | 4 | / | / | Range: 24-100% |
| (89) | / | / | / | / |
| (90) | / | / | / | / |
| (91) | 8 | 261 | 238 | RR 0.98; CI 0.95 to 1.02 |
| (92) | / | / | / | / |
| (93) | / | / | / | / |
| (94) | / | / | / | / |
| (95) | 9 | / | / | “Overall, adherence to supervised exercise therapy was approximately 80%, which was similar to that reported with home-based exercise therapy.” |
| (96) | / | 11 out of 101 (11%) | 25 out of 88 (28%) | RR 0.37, 95% CI 0.19 to 0.72 |
| (97) | 4 | / | / | Adherence range: 55-94% |
| (98) | / | / | / | / |
| (99) | / | / | / | / |
| (100) | / | / | / | / |
| (101) | 6 | / | / | Adherence range: 69-100% |
| (102) | / | / | / | / |
| (103) | 3 | / | / | MD 0.50; 95%CI -1.21 to 2.21  RR 0.03; 95%CI 0.00 to 0.54  RR 0.19; 95%CI 0.10 to 0.38 |
| (104) | 26 | 13% | 13% | In 80% of the trials, outcome assessment was available for those participants initially allocated to the groups |
| (105) | / | / | / | / |
| (106) | / | / | / | / |
| (107) | / | / | / | / |
| (108) | / | / | / | / |
| (109) | / | / | / | Adherence to intervention was unclear in one study |
| (110) | 38 | / | / | Adherence ranged from 25% to 100% |
| (111) | 33 | / | / | Adherence rate ranged from 39% to 95% |
| (112) | / | / | / | / |
| (113) | 7 | Range: 0 to 23.7% | Range: 0 to 14% | / |
| (114) | / | / | / | / |
| (115) | 10 | Range 0 to 22 % | Range 0 to 32% | / |
| (116) | 29 | / | / | Range: 42-91% |
| (117) | 9 | / | / | Range: 10-40% |
| (118) | 52 | See comment | See comment | Average adherence was 79% (range 36% to 163% of targeted session) for aerobic exercise |
| (119) | / | / | / | / |
| (120) | 6 | 458 | 385 | RR 1.63, 95% CI 0.77 to 3.43 |
| (121) | 15 | / | / | Drop-out range: 6-33% |
| (122) | 4 | / | / | Drop-out range: 0-2 participants |
| (123) | 2 | See comments | / | Mean attendance: 90% (SD 12.6); mean reported completion of daily home practice: 82% (SD 20.3) (in one study) |
| (124) | 34 | Range: 0 to 33% | Range: 0 to 46% | / |
| (125) | See comment | / | / | “These data are difficult to interpret because different definitions for adherence or compliance were used across the trials” |
| (126) | 6 | Range: 0 to 29% | Range: 6 to 58% | / |
| (127) | 11 | Range: 0 to 45% | Range: 0 to 16% | / |
| (128) | See comments | See comments | See comments | 66% completed the long-term follow-up |
| (129) | 2 | / | / | 10.7% withdrew |
| (130) | 14 | Range: 0 to 33% | Range: 0 to 29% | / |
| (131) | 13 | See comments | See comments | Dropout range: 24-42% |
| (132) | 4 | / | / | Range: 1-10 |
| (133) | / | / | / | / |
| (134) | 66 | Range: 0 to 64% | Range: 0 to 71% | / |
| (135) | 2 | 31% | 20% | OR 1.82, 95% CI 0.35 to 9. 45 |
| (136) | 1 | N=1 | N=2 | / |
| (137) | 4 | See comment | See comment | Range: 0% and 20%; RD -0.01, 95% CI -0.12 to 0.11 |
| (138) | 5 | See comment | See comment | Dropouts: 193 versus 116; RD -0.05, 95% CI -0.13 to 0.04 |
| (139) | 56 | 427 | 353 | RD 0.00, 95% CI -0.01 to 0.01 |
| (140) | 34 | Range: 0 and 47% | Range: 0 and 50% | / |
| (141) | 33 | Range: 0 and 47% | Range: 0 and 36% | / |
| (142) | 1 | See comments | See comments | 10% per study |
| (143) | 5 | Range: 0 to 21% | Range: 0 to 7% | / |
| (144) | 19 | Range: 3 to 31% | Range: 2 to 35% | / |
| (145) | 6 | Range: 0 and 28% | Range: 0 and 29% | / |
| (146) | 18 | See comments | See comments | In one trial, 5 of 18 (28%) dropped out (all from exercise group) |
| (147) | 6 | See comments | See comments | Range: 0 to 35% |
| (148) | 13 | Range: 4 to 48% | Range: 0 to 38% | Two studies reported that no participants withdrew |
| (149) | 6 | 157 | 152 | RR 2.88, 95% CI 0.30 to 27.18 |
| (150) | 5 | See comments | See comments | Withdrawals range: 5.5% to 10.1% |
| (151) | 9 | See comments | See comments | 18.75% noncompletion rate in one study |
| (152) | 4 | See comments | See comments | 5 |
| (153) | / | / | / | / |
| (154) | 1 | See comments | See comments | 9 of 13 (69%) patients were unable to complete intervention |
| (155) | 1 | / | 11.11% | / |
| (156) | 3 | 3 | 3 | Dropouts were due to medical reasons |
| (157) | 1 | / | > 40% | Reasons not provided |
| (158) | 2 | 3 | 1 | / |
| (159) | / | / | / | / |
| (160) | / | / | / | / |
| (161) | 9 | Range: 5 to 16% | >23% | / |
| (162) | 14 | Range: 20 to 30% | / | “The percentage of withdrawals was within the acceptable rate” |
| (163) | 48 | See comments | See comments | Often low <10% |
| (164) | 3 | / | Range: 20 to 45% | / |
| (165) | / | / | / | / |
| (166) | 33 | Range: 0 to 15% | / | / |
| (167) | 8 | / | / | Most drop-outs were not related to the intervention |
| (168) | 2 | / | / | / |
| (169) | 5 | Range: 0 to 13% | Range: 0 to 26% | / |
| (170) | 6 | See comments | See comments | Range: 3 to 33% |
| (171) | 2 | See comments | See comments | Range: 7 to 10% |
| (172) | 3 | See comments | See comments | Range: 2 to 12.5% |
| (173) | 22 | Range: 0 to 44 | Range: 0 to 40 | / |
| (174) | 8 | Range: 0 to 21% | Range: 0 to 5 % | No reasons for withdrawals given in two trials |
| (175) | 15 | Range: 11 to 75 % | Range: 4 to 68% | / |
| (176) | 7 | Range: 0 to 40% | Range: 0 to 42% | / |
| (177) | 2 | / | / | Range: 16-18% |
| (178) | 8 | Range: 0 to 25% | Range: 0 to 20% | / |
| (179) | 5 | See comments | See comments | Range: 0 to 10% |
| (180) | 3 | Range: 0 to 10% | Range: 0 to 30% | / |
| (181) | 12 | Range: 0 to 33% | Range: 0 to 55% | / |
| (182) | 7 | See comments | See comments | Range: 0 to 75% |
| (183) | 4 | / | / | 2 withdrew from one study |
| (184) | 2 | Range: 0 to 11% | Range: 0 to 13% | / |
| (185) | 10 | See comments | See comments | Range: 0 to > 20% |
| (186) | 13 | Range: 0 to 21% | Range: 0 to 33% | / |
| (187) | 12 | Range: 0 to 38% | Range: 0 to 27% | Dropout rates did not differ between the groups |
| (188) | 10 | Range: 0 to 29% | Range: 0 to 22% | / |

**Table 5 Footnote:**

a. results from one trial

/ Not reported

^a^ of those who completed the study

**Supplementary Table 6. Methodological quality assessment of the included Cochrane reviews with AMSTAR-2**

| **Reference** | **Research questions and inclusion criteria included the components of PICO?** | **Review methods were established prior to the conduct of the review?** | **Review authors explained their selection of the study designs for inclusion?** | **Review authors used a comprehen-sive literature search strategy?** | **Review authors perform study selection in duplicate?** | **Did the review authors perform data extraction in duplicate?** | **Did the review authors provide a list of excluded studies and justify the exclusions?** | **Did the review authors describe the included studies in adequate detail?** |
| --- | --- | --- | --- | --- | --- | --- | --- | --- |
| (39) | Yes | Yes | Yes | Yes | Yes | Yes | Yes | Yes |
| (40) | Yes | Yes | Yes | Yes | Yes | Yes | Yes | Yes |
| (41) | Yes | Yes | Yes | Yes | Yes | Yes | Yes | Yes |
| (42) | Yes | Yes | Yes | Yes | Yes | Yes | Yes | Yes |
| (43) | Yes | Yes | Yes | Yes | Yes | Yes | Yes | Yes |
| (44) | Yes | Yes | Yes | Yes | Yes | Yes | Yes | Yes |
| (45) | Yes | Yes | Yes | Yes | Yes | Yes | Yes | Yes |
| (46) | Yes | Yes | Yes | Yes | Yes | Yes | No | Partial Yes |
| (47) | Yes | Yes | Yes | Yes | Yes | Yes | Yes | Yes |
| (48) | Yes | Yes | Yes | Yes | Yes | Yes | Yes | Yes |
| (49) | Yes | Yes | Yes | Yes | Yes | Yes | Yes | Yes |
| (50) | Yes | Yes | Yes | Yes | Yes | Yes | Yes | Yes |
| (51) | Yes | Yes | Yes | Yes | Yes | Yes | Yes | Yes |
| (52) | Yes | Yes | Yes | Yes | Yes | Yes | Yes | Yes |
| (53) | Yes | Yes | Yes | Yes | Yes | Yes | Yes | Yes |
| (54) | Yes | Yes | Yes | Yes | Yes | Yes | Yes | Yes |
| (55) | Yes | Yes | Yes | Yes | Yes | Yes | Yes | Yes |
| (56) | Yes | Yes | Yes | Yes | Yes | Yes | Yes | Yes |
| (57) | Yes | Yes | Yes | Yes | Yes | Yes | Yes | Yes |
| (58) | Yes | Yes | Yes | Yes | Yes | Yes | Yes | Yes |
| (59) | Yes | No | Yes | Yes | Yes | Yes | Yes | Yes |
| (60) | Yes | Yes | Yes | Yes | Yes | Yes | Yes | Yes |
| (61) | Yes | Yes | Yes | Yes | Yes | Yes | Yes | Yes |
| (62) | Yes | Yes | Yes | Yes | Yes | Yes | Yes | Yes |
| (63) | Yes | Yes | Yes | Yes | Yes | Yes | Yes | Yes |
| (64) | Yes | Yes | Yes | Yes | Yes | Yes | Yes | Yes |
| (65) | Yes | Yes | Yes | Yes | Yes | Yes | Yes | Yes |
| (66) | Yes | Yes | Yes | Yes | Yes | Yes | Yes | Yes |
| (67) | Yes | Yes | Yes | Yes | Yes | Yes | Yes | Yes |
| (68) | Yes | Yes | Yes | Yes | Yes | Yes | Yes | Yes |
| (69) | Yes | Yes | Yes | Yes | Yes | Yes | Yes | Yes |
| (70) | Yes | Yes | Yes | Yes | Yes | Yes | Yes | Yes |
| (71) | Yes | Yes | Yes | Yes | Yes | Yes | Yes | Yes |
| (72) | Yes | Yes | No | Yes | Yes | Yes | Yes | Yes |
| (73) | Yes | Yes | Yes | Yes | Yes | Yes | Yes | Yes |
| (74) | Yes | Yes | No | Yes | Yes | Yes | No | Yes |
| (75) | Yes | Yes | No | Yes | Yes | Yes | Yes | Yes |
| (76) | Yes | Yes | No | Yes | Yes | Yes | Yes | Yes |
| (77) | Yes | Yes | No | Yes | Yes | Yes | Yes | No |
| (78) | Yes | Yes | Yes | Yes | Yes | Yes | Yes | Yes |
| (79) | Yes | Yes | Yes | Yes | Yes | Yes | Yes | Yes |
| (80) | Yes | Yes | No | Yes | Yes | Yes | Yes | No |
| (81) | Yes | Yes | No | Yes | Yes | Yes | Yes | Yes |
| (82) | Yes | Yes | Yes | Yes | Yes | Yes | Yes | Yes |
| (83) | Yes | Yes | No | Yes | Yes | Yes | Yes | Yes |
| (84) | Yes | Yes | No | Yes | Yes | Yes | Yes | Yes |
| (85) | Yes | Yes | No | Yes | Yes | Yes | Yes | Yes |
| (86) | Yes | Yes | Yes | Yes | Yes | Yes | Yes | Yes |
| (87) | Yes | Yes | Yes | Yes | Yes | Yes | Yes | Yes |
| (88) | Yes | Yes | No | Yes | Yes | Yes | Yes | Yes |
| (89) | Yes | Yes | Yes | Yes | Yes | Yes | Yes | Yes |
| (90) | Yes | Yes | No | Yes | Yes | Yes | Yes | Yes |
| (91) | Yes | Yes | Yes | Yes | Yes | Yes | Yes | Yes |
| (92) | Yes | Yes | No | Yes | Yes | Yes | Yes | Yes |
| (93) | Yes | Yes | No | Yes | Yes | Yes | Yes | Yes |
| (94) | Yes | Yes | Yes | Yes | Yes | Yes | Yes | Yes |
| (95) | Yes | Yes | Yes | Yes | Yes | Yes | Yes | Yes |
| (96) | Yes | Yes | No | Yes | Yes | Yes | Yes | No |
| (97) | Yes | Yes | No | Yes | Yes | Yes | Yes | Yes |
| (98) | Yes | Yes | No | Yes | Yes | Yes | Yes | Yes |
| (99) | Yes | Yes | No | Yes | Yes | Yes | Yes | Yes |
| (100) | Yes | Yes | Yes | Yes | Yes | Yes | Yes | Yes |
| (101) | Yes | Yes | Yes | Yes | Yes | Yes | Yes | Yes |
| (102) | Yes | Yes | No | Yes | Yes | Yes | Yes | No |
| (103) | Yes | Yes | No | Yes | Yes | Yes | Yes | Yes |
| (104) | Yes | Yes | No | Yes | Yes | Yes | Yes | Yes |
| (105) | Yes | Yes | Yes | Yes | Yes | Yes | Yes | Yes |
| (106) | Yes | Yes | Yes | Yes | Yes | Yes | Yes | Yes |
| (107) | Yes | Yes | Yes | Yes | Yes | Yes | Yes | Yes |
| (108) | Yes | Yes | Yes | Yes | Yes | Yes | Yes | Yes |
| (109) | Yes | Yes | Yes | Yes | Yes | Yes | Yes | Yes |
| (110) | Yes | Yes | No | Yes | Yes | Yes | Yes | Yes |
| (111) | Yes | Yes | No | Yes | Yes | Yes | Yes | Yes |
| (112) | Yes | Yes | No | Yes | Yes | Yes | Yes | Yes |
| (113) | Yes | Yes | No | Yes | Yes | Yes | Yes | Yes |
| (114) | Yes | No | No | Yes | Yes | Yes | Yes | Yes |
| (115) | Yes | No | No | Yes | Yes | Yes | Yes | Yes |
| (116) | Yes | No | Yes | Yes | Yes | Yes | Yes | Yes |
| (117) | No | No | No | Partial Yes | Yes | Yes | Yes | Partial Yes |
| (118) | Yes | No | No | Partial Yes | Yes | Yes | Yes | Yes |
| (119) | Yes | No | No | Partial Yes | Yes | Yes | Yes | Yes |
| (120) | Yes | No | No | Yes | Yes | Yes | Yes | Yes |
| (121) | Yes | No | No | No | Yes | Yes | Yes | Yes |
| (122) | Yes | No | No | Partial Yes | Yes | Yes | Yes | Partial Yes |
| (123) | Yes | No | No | Partial Yes | Yes | Yes | Yes | Yes |
| (124) | Yes | Yes | No | Partial Yes | Yes | Yes | Yes | Yes |
| (125) | Yes | No | No | Partial Yes | Yes | Yes | Yes | Yes |
| (126) | Yes | No | Yes | Yes | Yes | Yes | Yes | Yes |
| (127) | Yes | No | No | Partial Yes | Yes | Yes | Yes | Yes |
| (128) | Yes | No | Yes | Partial Yes | Yes | Yes | Yes | Yes |
| (129) | Yes | No | No | Partial Yes | Yes | Yes | Yes | Yes |
| (130) | Yes | Yes | No | Yes | Yes | Yes | Yes | Yes |
| (131) | Yes | No | No | Partial Yes | Yes | Yes | Yes | Partial Yes |
| (132) | Yes | No | No | Partial Yes | Yes | Yes | Yes | Partial Yes |
| (133) | Yes | No | No | Yes | Yes | Yes | Yes | Yes |
| (134) | Yes | No | No | Partial Yes | Yes | Yes | Yes | Partial Yes |
| (135) | Yes | No | Yes | Yes | Yes | Yes | Yes | Yes |
| (136) | Yes | No | No | Partial Yes | Yes | Yes | Yes | Partial Yes |
| (137) | Yes | No | Yes | Yes | Yes | Yes | Yes | Partial Yes |
| (138) | Yes | No | No | Yes | Yes | Yes | Yes | Partial Yes |
| (139) | Yes | Yes | No | Yes | Yes | Yes | Yes | Yes |
| (140) | Yes | No | No | Yes | Yes | Yes | Yes | Yes |
| (141) | Yes | No | No | Yes | Yes | Yes | Yes | Yes |
| (142) | Yes | No | No | No | Yes | Yes | Yes | Partial Yes |
| (143) | Yes | No | No | Partial Yes | Yes | Yes | Yes | Yes |
| (144) | Yes | No | No | Partial Yes | Yes | Yes | Yes | Yes |
| (145) | Yes | No | No | Yes | Yes | Yes | Yes | Yes |
| (146) | Yes | No | No | Partial Yes | Yes | Yes | Yes | Yes |
| (147) | Yes | No | No | Partial Yes | Yes | Yes | Yes | Partial Yes |
| (148) | Yes | No | No | Yes | Yes | Yes | Yes | Partial Yes |
| (149) | Yes | No | No | Partial Yes | Yes | Yes | Yes | Yes |
| (150) | Yes | No | No | Partial Yes | Yes | Yes | Yes | Yes |
| (151) | Yes | No | No | Partial Yes | Yes | Yes | Yes | Yes |
| (152) | Yes | Partial Yes | No | Partial Yes | Yes | Yes | Yes | Yes |
| (153) | Yes | No | No | Yes | Yes | Yes | Yes | Yes |
| (154) | Yes | Yes | No | Partial Yes | Yes | Yes | Yes | Yes |
| (155) | Yes | No | No | Yes | Yes | Yes | Yes | Yes |
| (156) | Yes | Yes | No | Yes | Yes | Yes | Yes | Yes |
| (157) | Yes | No | Yes | No | Yes | Yes | Yes | Yes |
| (158) | No | No | No | Partial Yes | Yes | Yes | Yes | Partial Yes |
| (159) | Yes | No | No | Partial Yes | Yes | Yes | Yes | Yes |
| (160) | Yes | No | Yes | Yes | Yes | Yes | Yes | Yes |
| (161) | Yes | Yes | No | Partial Yes | Yes | Yes | Yes | Yes |
| (162) | Yes | No | No | Partial Yes | Yes | Yes | Yes | Yes |
| (163) | Yes | No | Yes | Yes | Yes | Yes | Yes | Yes |
| (164) | Yes | No | No | Partial Yes | Yes | Yes | Yes | Partial Yes |
| (165) | Yes | No | Yes | Partial Yes | Yes | Yes | Yes | Yes |
| (166) | Yes | No | No | Partial Yes | Yes | No | Yes | Yes |
| (167) | Yes | Partial Yes | No | Partial Yes | Yes | Yes | Yes | Yes |
| (168) | Yes | Yes | No | Yes | Yes | Yes | Yes | Yes |
| (169) | Yes | No | No | Partial Yes | Yes | Yes | Yes | Yes |
| (170) | Yes | No | No | Yes | Yes | Yes | Yes | Yes |
| (171) | Yes | No | No | Partial Yes | Yes | Yes | Yes | Yes |
| (172) | Yes | No | No | No | Yes | Yes | Yes | Partial Yes |
| (173) | Yes | No | No | Partial Yes | Yes | Yes | Yes | Yes |
| (174) | Yes | No | Yes | Yes | Yes | Yes | Yes | Yes |
| (175) | Yes | No | No | No | No | No | Yes | Yes |
| (176) | Yes | Yes | No | Partial Yes | Yes | Yes | Yes | Yes |
| (177) | Yes | No | No | Yes | Yes | Yes | Yes | Yes |
| (178) | Yes | No | No | Yes | Yes | Yes | Yes | Yes |
| (179) | Yes | No | No | No | Yes | Yes | Yes | Yes |
| (180) | Yes | No | No | No | Yes | Yes | Yes | Partial Yes |
| (181) | Yes | Yes | No | Yes | Yes | Yes | Yes | Yes |
| (182) | Yes | Yes | Yes | Yes | Yes | Yes | Yes | Yes |
| (183) | Yes | No | No | Partial Yes | Yes | Yes | Yes | Yes |
| (184) | Yes | No | No | Yes | Yes | Yes | Yes | Yes |
| (185) | Yes | Yes | Yes | Partial Yes | Yes | Yes | Yes | Yes |
| (186) | Yes | No | No | Yes | Yes | Yes | Yes | Yes |
| (187) | Yes | No | No | Yes | Yes | Yes | Yes | Yes |
| (188) | Yes | No | No | Partial Yes | Yes | Yes | Yes | Yes |

**TABLE 6. AMSTAR-2 (continued)**

| **Reference** | **Review authors used a satisfactory technique for assessing RoB in individual studies?** | **Review authors reported on the sources of funding for the studies?** | **Review authors use appropriate methods for statistical combination of results (if meta-analysed)?** | **Review authors assessed the potential impact of RoB in individual studies on the results of the meta-analysis or other evidence synthesis (if meta-analysed)?** | **Review authors accounted for RoB in individual studies when interpreting/ discussing the results?** | **Review authors provided a satisfactory explanation for, and discussion of, any heterogeneity observed in the results?** | **Review authors carried out an adequate investigation of publication bias and discussed its likely impact on the results of the review (if meta-analysed)?** | **Review authors reported any potential sources of conflict of interest, including any funding they received for conducting the review?** |
| --- | --- | --- | --- | --- | --- | --- | --- | --- |
| (39) | Yes | No | n/a | n/a | Yes | Yes | n/a | Yes |
| (40) | Yes | Yes | Yes | Yes | Yes | Yes | Yes | Yes |
| (41) | Yes | No | Yes | Yes | Yes | Yes | Yes | Yes |
| (42) | Yes | Yes | Yes | Yes | Yes | Yes | Yes | Yes |
| (43) | Yes | Yes | Yes | Partial Yes | Yes | Yes | Yes | Yes |
| (44) | No | No | Yes | Yes | No | No | No | Yes |
| (45) | Yes | No | n/a | n/a | Yes | Yes | n/a | Yes |
| (46) | Partial Yes | No | n/a | n/a | No | No | n/a | Yes |
| (47) | Yes | Yes | Yes | Partial Yes | Yes | Yes | Yes | Yes |
| (48) | Yes | Yes | Yes | Yes | Yes | Yes | No | Yes |
| (49) | Yes | Yes | Yes | Yes | Yes | Yes | Yes | Yes |
| (50) | Yes | Yes | Yes | Yes | Yes | Yes | Yes | Yes |
| (51) | Yes | Yes | Yes | Yes | Yes | No | Yes | Yes |
| (52) | Yes | Yes | Yes | Yes | Yes | Yes | Yes | Yes |
| (53) | Yes | No | Yes | Yes | Yes | Yes | Yes | Yes |
| (54) | Yes | Yes | Yes | Yes | Yes | Yes | No | Yes |
| (55) | Yes | Yes | Yes | Yes | Yes | Yes | No | Yes |
| (56) | Yes | Yes | Yes | Yes | Yes | Yes | No | Yes |
| (57) | Yes | No | Yes | Yes | Yes | No | No | Yes |
| (58) | Yes | Yes | Yes | Yes | Yes | Yes | No | Yes |
| (59) | Yes | No | Yes | Yes | Yes | Yes | No | Yes |
| (60) | Yes | Yes | Yes | Yes | Yes | No | Yes | Yes |
| (61) | Yes | No | Yes | Yes | Yes | Yes | Yes | Yes |
| (62) | Yes | No | Yes | Yes | Yes | Yes | Yes | Yes |
| (63) | Yes | No | Yes | Yes | Yes | Yes | Yes | Yes |
| (64) | Yes | No | Yes | Yes | Yes | Yes | Yes | Yes |
| (65) | Yes | No | Yes | Yes | Yes | No | Yes | Yes |
| (66) | Yes | No | Yes | Yes | Yes | No | No | Yes |
| (67) | Yes | No | Yes | Yes | No | No | No | Yes |
| (68) | Yes | Yes | Yes | Yes | Yes | Yes | Yes | Yes |
| (69) | Yes | Yes | n/a | n/a | Yes | n/a | n/a | Yes |
| (70) | Yes | No | Yes | Yes | Yes | Yes | Yes | Yes |
| (71) | Yes | No | Yes | Yes | Yes | Yes | Yes | Yes |
| (72) | Yes | No | Yes | Yes | Yes | Yes | Yes | Yes |
| (73) | Yes | No | Yes | Yes | Yes | Yes | Yes | Yes |
| (74) | Yes | No | Yes | Yes | Yes | Yes | No | Yes |
| (75) | Yes | Yes | Yes | Yes | Yes | Yes | No | Yes |
| (76) | Yes | No | Yes | Yes | Yes | Yes | Yes | Yes |
| (77) | Yes | No | Yes | No | Yes | Yes | Yes | Yes |
| (78) | Yes | No | Yes | Yes | Yes | Yes | No | Yes |
| (79) | Yes | Yes | n/a | n/a | Yes | Yes | n/a | Yes |
| (80) | No | No | Yes | No | No | No | No | Yes |
| (81) | Yes | Yes | Yes | Yes | Yes | Yes | No | Yes |
| (82) | Yes | Yes | Yes | Yes | Yes | Yes | Yes | Yes |
| (83) | Yes | No | Yes | Yes | Yes | Yes | Yes | Yes |
| (84) | Yes | No | Yes | Yes | Yes | Yes | Yes | Yes |
| (85) | Yes | No | Yes | Yes | Yes | Yes | Yes | Yes |
| (86) | Yes | No | Yes | Yes | Yes | Yes | Yes | Yes |
| (87) | Yes | Yes | Yes | Yes | Yes | Yes | Yes | Yes |
| (88) | Yes | No | Yes | Yes | Yes | No | Yes | Yes |
| (89) | Yes | No | Yes | Yes | Yes | Yes | Yes | Yes |
| (90) | Yes | No | Yes | Yes | Yes | Yes | Yes | Yes |
| (91) | Yes | Yes | Yes | Yes | Yes | Yes | No | Yes |
| (92) | Yes | Yes | Yes | Yes | Yes | Yes | Yes | Yes |
| (93) | Yes | Yes | n/a | n/a | Yes | Yes | n/a | Yes |
| (94) | Yes | No | Yes | Yes | Yes | Yes | Yes | Yes |
| (95) | Yes | No | Yes | Yes | Yes | Yes | Yes | Yes |
| (96) | Yes | No | Yes | No | No | No | No | Yes |
| (97) | Yes | No | Yes | Yes | Yes | Yes | Yes | Yes |
| (98) | Yes | No | Yes | Yes | Yes | Yes | No | Yes |
| (99) | Yes | No | Yes | Yes | Yes | Yes | Yes | Yes |
| (100) | Yes | No | n/a | n/a | Yes | Yes | n/a | Yes |
| (101) | Yes | Yes | Yes | Yes | Yes | Yes | Yes | Yes |
| (102) | No | No | Yes | No | No | Yes | Yes | Yes |
| (103) | Yes | Partial Yes | Partial Yes | Partial Yes | Yes | Partial Yes | Yes | Yes |
| (104) | No | No | Yes | Yes | No | Yes | Yes | Yes |
| (105) | Yes | No | Yes | Yes | Yes | Yes | Yes | Yes |
| (106) | Yes | No | Yes | Yes | Yes | Yes | Yes | Yes |
| (107) | Yes | No | Yes | Yes | Yes | Yes | Yes | Yes |
| (108) | No | No | Yes | No | No | No | No | Yes |
| (109) | Yes | No | Yes | Yes | Yes | No | Yes | Yes |
| (110) | Yes | No | Yes | Yes | Yes | Yes | Yes | Yes |
| (111) | Yes | No | Yes | Yes | Yes | Yes | Yes | Yes |
| (112) | Partial Yes | No | Yes | Yes | No | Yes | No | Yes |
| (113) | Yes | No | Yes | Yes | Yes | Yes | No | Yes |
| (114) | Yes | No | n/a | n/a | No | No | n/a | Yes |
| (115) | Yes | No | Yes | Yes | Yes | Yes | Yes | Yes |
| (116) | Yes | No | Yes | Yes | Yes | Yes | Yes | Yes |
| (117) | Yes | No | Yes | No | Yes | No | No | Yes |
| (118) | Yes | Yes | Yes | Yes | Yes | Yes | Yes | Yes |
| (119) | Yes | No | Yes | Yes | Yes | Yes | Yes | Yes |
| (120) | Yes | No | Yes | Yes | Yes | Yes | Yes | Yes |
| (121) | No | No | Yes | Yes | No | Yes | No | Yes |
| (122) | Yes | No | Yes | Yes | Yes | Yes | Yes | Yes |
| (123) | Yes | No | No | Yes | Yes | Yes | Yes | Yes |
| (124) | Yes | Yes | Yes | Yes | Yes | Yes | No | Yes |
| (125) | Partial Yes | No | Yes | Yes | Yes | Yes | No | Yes |
| (126) | Yes | Yes | Yes | Yes | Yes | Yes | Yes | Yes |
| (127) | Yes | Yes | Yes | Yes | Yes | Yes | Yes | Yes |
| (128) | Yes | Yes | Yes | Yes | Yes | Yes | Yes | Yes |
| (129) | Yes | Yes | Yes | Yes | Yes | Yes | Yes | Yes |
| (130) | Yes | Yes | Yes | Yes | Yes | Yes | Yes | Yes |
| (131) | Yes | No | Yes | Yes | Yes | Yes | Yes | Yes |
| (132) | Yes | No | Yes | Yes | Yes | Yes | Yes | Yes |
| (133) | Partial Yes | No | No | Yes | No | Yes | No | Yes |
| (134) | Yes | Yes | Yes | Yes | Yes | Yes | Yes | Yes |
| (135) | Yes | No | Yes | Yes | Yes | Yes | Yes | Yes |
| (136) | No | No | No | Yes | No | No | No | Yes |
| (137) | Partial Yes | No | Yes | Yes | No | Yes | No | Yes |
| (138) | Partial Yes | No | Yes | Yes | No | Yes | No | Yes |
| (139) | Partial Yes | No | Yes | Yes | Yes | Yes | Yes | Yes |
| (140) | Yes | Yes | Yes | Yes | Yes | Yes | Yes | Yes |
| (141) | Yes | Yes | Yes | Yes | Yes | Yes | Yes | Yes |
| (142) | Partial Yes | No | No | No | No | No | No | Yes |
| (143) | Yes | Yes | Yes | Yes | Yes | Yes | Yes | Yes |
| (144) | Yes | No | Yes | Yes | Yes | Yes | Yes | Yes |
| (145) | Yes | Yes | Yes | Yes | Yes | Yes | Yes | Yes |
| (146) | Partial Yes | No | Yes | Yes | Yes | No | No | Yes |
| (147) | Partial Yes | No | Yes | Yes | Yes | Yes | No | Yes |
| (148) | Partial Yes | No | Yes | Yes | Yes | Yes | No | Yes |
| (149) | Yes | No | Yes | Yes | Yes | Yes | Yes | Yes |
| (150) | Yes | Yes | Yes | Yes | Yes | Yes | Yes | Yes |
| (151) | Yes | Yes | No | No | Yes | Yes | No | No |
| (152) | Yes | No | No | No | Yes | Yes | No | Yes |
| (153) | Yes | Yes | No | No | No | No | No | Yes |
| (154) | Yes | Yes | No | No | No | No | No | Yes |
| (155) | Yes | No | Yes | Yes | Yes | Yes | Yes | Yes |
| (156) | Yes | Yes | Yes | No | Yes | Yes | No | Yes |
| (157) | Yes | No | No | No | Yes | Yes | No | Yes |
| (158) | Partial Yes | No | No | No | No | No |  | Yes |
| (159) | Yes | Yes | Yes | Yes | Yes | Yes | No | Yes |
| (160) | Yes | No | No | No | Yes | No | No | Yes |
| (161) | Yes | Yes | Yes | No | Yes | Yes | Yes | Yes |
| (162) | Yes | Yes | No | Yes | Yes | Yes | Yes | Yes |
| (163) | Yes | No | Yes | No | Yes | Yes | Yes | Yes |
| (164) | Yes | No | Yes | No | Yes | Yes | No | Yes |
| (165) | Yes | No | No | No | Yes | No | No | Yes |
| (166) | No | No | Yes | Yes | Yes | Yes | Yes | Yes |
| (167) | Yes | Yes | Yes | Yes | Yes | Yes | Yes | Yes |
| (168) | Yes | Yes | No | No | Yes | Yes | No | Yes |
| (169) | Yes | No | Yes | Yes | Yes | Yes | No | Yes |
| (170) | Yes | No | No | Yes | Yes | Yes | Yes | Yes |
| (171) | Yes | No | Yes | No | Yes | Yes | Yes | Yes |
| (172) | Partial Yes | No | Yes | Yes | Yes | Yes | Yes | Yes |
| (173) | Yes | No | Yes | Yes | Yes | Yes | Yes | Yes |
| (174) | Partial Yes | No | Yes | Yes | Yes | Yes | Yes | Yes |
| (175) | Yes | No | No | No | Yes | Yes | No | Yes |
| (176) | Yes | Yes | No | No | Yes | Yes | No | Yes |
| (177) | Yes | No | Yes | Yes | Yes | Yes | No | Yes |
| (178) | Yes | No | Yes | Yes | Yes | Yes | Yes | Yes |
| (179) | Yes | No | No | No | Yes | Yes | No | Yes |
| (180) | Yes | No | No | No | Yes | Yes | No | Yes |
| (181) | Yes | Yes | Yes | Yes | Yes | Yes | Yes | Yes |
| (182) | Yes | Yes | Yes | Yes | Yes | Yes | Yes | Yes |
| (183) | Yes | Yes | Yes | Yes | Yes | Yes | Yes | Yes |
| (184) | Yes | Yes | Yes | Yes | Yes | Yes | Yes | Yes |
| (185) | Yes | Yes | Yes | Yes | Yes | Yes | Yes | Yes |
| (186) | Yes | No | Yes | Yes | Yes | Yes | Yes | Yes |
| (187) | Yes | No | Yes | Yes | Yes | Yes | No | Yes |
| (188) | Yes | No | Yes | Yes | Yes | Yes | No | Yes |

**Supplementary Table 7.** **Number of studies assessed as low risk of bias per domain**

| **Reference** | **Total number**  **of studies**  **in assessment** | **Selection bias** | | **Performance**  **bias** | **Detection**  **bias** | **Attrition**  **bias** | **Reporting**  **bias** | **Other bias** |
| --- | --- | --- | --- | --- | --- | --- | --- | --- |
|  |  | **Random**  **sequence**  **generation** | **Allocation**  **concealment** |  |  |  |  |  |
| (39) | 4 | 0 | 0 | 0 | 0 | 1 | 2 | 2 |
| (40) | 44 | 22 | 11 | 1 | 44 | 17 | 13 | 32 |
| (41) | 14 | 9 | 6 | 2^b^ | 2^b^ | 6 | 10 | 2 |
| (42) | 10 | 6 | 2 | / | 3 | 7 | 10 | 8 |
| (43) | 63 | 16 | 13 | 0 | 16 | 25 | 56 | 47 |
| (44) | 3 | 1 | 1 | 0 | 0 | 0 | 0 | / |
| (45) | 2 | 0 | 1 | 0 | 0 | 0 | 0 | / |
| (46) | 6 | / | 3 | / | / | / | / | / |
| (47) | 13 | 8 | 8 | 0 | 0 | 7 | 11 | 11 |
| (48) | 8 | 4 | 1 | 0 | 0 | 4 | 3 | 0 |
| (49) | 9 | 3 | 3 | 0 | 1 | 4 | 1 | 4 |
| (50) | 13 | 7 | 4 | 2 | 1 | 12 | 10 | 9 |
| (51) | 4 | 3 | 2 | 2 | 4 | 4 | 2 | 3 |
| (52) | 16 | 11 | 8 | 8 | 13 | 15 | 9 | 11 |
| (53) | 6 | 2 | 0 | 0 | 2 | 1 | 4 | 2 |
| (54) | 3 | 2 | 2 | 0 | 0 | 3 | 2 | 3 |
| (55) | 6 | 5 | 2 | 0 | 4 | 1 | 4 | 0 |
| (56) | 8 | 5 | 3 | 0 | 5 | 2 | 6 | 0 |
| (57) | 3 | 1 | 0 | 0 | 1 | 1 | 1 | 0 |
| (58) | 11 | 4 | 3 | 0 | 2 | 3 | 1 | 3 |
| (59) | 34 | 17 | 10 | 8 | 20 | Average: 27% | 32 | 5 |
| (60) | 5 | 4 | 2 | 1 | 2 | 5 | 3 | 5 |
| (61) | 95 | 66 | 39 | 7 | 10 | 61 | 50 | 45 |
| (62) | 21 | 7 | 2 | 0 | 2 | 8 | 0 | 3 |
| (63) | 3 | 3 | 1 | 2^b^ | 2^b^ | 3 | 1 | 2 |
| (64) | 5 | 0 | 0 | 0 | 2 | 2 | 0 | 3 |
| (65) | 3 | 3 | 3 | 0 | 1 | 1 | 0 | 0 |
| (66) | 3 | 1 | 1 | 0 | 0 | 0 | 0 | 0 |
| (67) | 9 | 8 | 5 | 0 | 0 | 8 | 0 | 6 |
| (68) | 7 | 7 | 3 | 1 | 7 | 3 | 3 | 4 |
| (69) | 6 | 4 | 4 | 0 | 5 | 3 | 3 | 4 |
| (70) | 39 | 11 | 14 | 0 | 1^a^ | 14 | 10 | 2 |
| (71) | 42 | 28 | 10 | 34^b^ | 34^b^ | 16 | / | / |
| (72) | 24 | 17 | 15 | 1 | 5 | 15 | 18 | 6 |
| (73) | 56 | 34 | 19 | / | 0 | 39 | 55 | / |
| (74) | 2 | 2 | 0 | 1 | 0 | 1 | 1 | 2 |
| (75) | 2 | 2 | 1 | 2 | 2 | 2 | 2 | 2 |
| (76) | 5 | 3 | 2 | 0 | 1 | 2 | 1 | 2 |
| (77) | 9 | 2 | 4 | / | / | / | / | / |
| (78) | 44 | 6 | 42 | 10^b^ | 10^b^ | 22 | 37 | 27 |
| (79) | 4 | 4 | 3 | 0 | 4 | 1 | 4 | 2 |
| (80) | 23 | / | 3 | / | / | / | / | / |
| (81) | 11 | 8 | 8 | 8 | 8 | 3 | 1 | 11 |
| (82) | 1 | 1 | 1 | 0 | 0 | 0 | 0 | 1 |
| (83) | 17 | 6 | 5 | 1 | 12 | 10 | 17 | 16 |
| (84) | 54 | 39 | 25 | 3 | 4 | 33 | 11 | / |
| (85) | 10 | 8 | 7 | 0 | 0 | 7 | 4 | 7 |
| (86) | 13 | 4 | 2 | 0 | 4 | 10 | 8 | 4 |
| (87) | 32 | 20 | 19 | 0 | 9 | 23 | 7 | 19 |
| (88) | 7 | 4 | 4 | 1 | 5 | 4 | 0 | / |
| (89) | 159 | 99 | 60 | 29 | 75 | 66 | / | 87 |
| (90) | 3 | 3 | 1 | 0^b^ | 0^b^ | 1 | 0 | 0 |
| (91) | 11 | 1 | 0 | 0 | 0 | 6 | 2 | 11 |
| (92) | 6 | 3 | 1 | 1 | 1 | 5 | 2 | 5 |
| (93) | 3 | 1 | 0 | 0 | 1 | 2 | 0 | 0 |
| (94) | 27 | 50% | 57% | 7% | | 71% | 21% | 29% |
| (95) | 21 | 17 | 11 | 0 | 3 | 15 | 17 | 6 |
| (96) | 3 | / | 0 | / | / | / | / | / |
| (97) | 5 | 2 | 2 | 0 | 0 | 2 | 3 | 3 |
| (98) | 11 | 1 | 1 | 0 | 1 | 1 | 4 | 0 |
| (99) | 13 | 5 | 1 | 0 | 2 | 2 | 6 | 0 |
| (100) | 11 | 1 | 1 | 0 | 1 | 0 | 2 | 0 |
| (101) | 8 | 6 | 4 | 0 | 7 | 3 | 0 | 7 |
| (102) | 61 | / | / | / | / | / | / | / |
| (103) | 21 | 11 | 5 | 2 ^b^ | 2 ^b^ | 2 | 20 | 12 |
| (104) | 45 | 60% | 46% | 0% | 0% | 70% | 89% | 76% |
| (105) | 45 | 11 | 11 | 3 | 6 | 14 | / | / |
| (106) | 12 | 8 | 8 | / | / | / | 1 | 5 |
| (107) | 12 | 4 | 1 | 0 | 0 | 2 | 1 | 0 |
| (108) | 19 | 6 | 5 | 1 | 4 | / | / | / |
| (109) | 16 | 6 | 1 | 1 | 3 | 3 | 6 | 11 |
| (110) | 94 | 40 | 27 | 16 | 37 | 31 | 24 | 74 |
| (111) | 43 | 16 | 11 | 1 | 14 | 15 | 1 | 37 |
| (112) | 8 | 8 | 1 | / | 4 | 5 | 0 | 1 |
| (113) | 21 | 15 | 10 | 0 | 12 | 13 | 21 | 20 |
| (114) | 1 | 0 | 0 | / | 0 | 0 | 0 | 0 |
| (115) | 12 | 7 | 6 | 0 | 5 | 6 | 1 | 8 |
| (116) | 30 | 11 | 2 | 0 | 0 | 17 | 1 | 4 |
| (117) | 14 | 3 | 2 | 0 | / | 10 | 12 | 12 |
| (118) | 63 | 32 | 22 | 0 | 24 | 18 | 55 | 45 |
| (119) | 32 | 17 | 15 | 32 | 7 | 20 | 29 | 11 |
| (120) | 8 | 7 | 5 | 0 | 0 | 5 | 2 | 6 |
| (121) | 16 | 1 | 0 | / | / | / | / | / |
| (122) | 5 | 4 | 2 | 5 | 3 | 4 | 5 | 4 |
| (123) | 2 | 2 | 2 | 0 | 1 | 0 | 0 | 0 |
| (124) | 38 | 17 | 11 | / | / | 2 | 17 | 10 |
| (125) | 121 | 19 | 11 | / | 33 | / | / | / |
| (126) | 7 | 2 | 1 | / | 2 | 4 | 0 | / |
| (127) | 11 | 8 | 7 | 0 | 3 | 8 | 7 | 7 |
| (128) | 3 | 3 | 2 | 0 | 0 | 1 | 3 | / |
| (129) | 3 | 1 | 0 | 0 | 1 | 2 | 3 | 0 |
| (130) | 18 | 9 | 5 | 0 | 8 | 5 | 10 | 15 |
| (131) | 15 | 7 | 5 | 0 | 6 | 7 | 5 | 13 |
| (132) | 5 | 2 | 2 | 0 | 2 | 1 | 5 | / |
| (133) | 24 | 9 | 3 | 0 | 9 | / | / | / |
| (134) | 70 | 48 | 49 | 3 | 21 | 27 | 17 | 6 |
| (135) | 3 | 1 | 1 | 0 | 1 | 2 | 3 | 1 |
| (136) | 2 | / | 2 | / | 1 | / | / | / |
| (137) | 4 | 0 | 2 | 0 | 3 | / | / | / |
| (138) | 5 | 1 | 0 | / | 2 | / | / | / |
| (139) | 56 | 29 | 23 | / | 25 | / | / | / |
| (140) | 40 | 22 | 17 | 0 | 5 | 12 | 38 | 32 |
| (141) | 56 | 33 | 18 | 0 | 0 | 16 | 47 | 50 |
| (142) | 1 | 0 | 1 | 0 | 1 | / | / | / |
| (143) | 6 | 3 | 0 | 0 | 5 | 4 | 3 | 3 |
| (144) | 65 | 45 | 34 | 9 | 19 | 29 | 13 | 32 |
| (145) | 12 | 8 | 3 | 0 | 6 | 5 | 3 | / |
| (146) | 21 | 13 | 10 | / | 4 | 5 | / | / |
| (147) | 7 | 4 | / | 0 | 2 | / | / | / |
| (148) | 14 | 5 | 1 | 0 | 1 | / | / | / |
| (149) | 7 | 5 | 4 | 1 | 2 | 3 | 5 | 5 |
| (150) | 32 | 11 | 8 | 3 | 17 | 24 | 2 | 32 |
| (151) | 60 | 39 | 25 | 13 | 12 | 50 | 12 | 58 |
| (152) | 16 | 8 | 3 | 4 | 9 | 8 | 8 | 16 |
| (153) | 2 | 2 | 0 | 0 | 0 | 2 | 2 | 2 |
| (154) | 6 | 4 | 1 | 1 | 3 | 0 | 3 | 1 |
| (155) | 15 | 4 | 2 | 1 | 6 | 7 | 7 | 2 |
| (156) | 6 | 5 | 1 | 0 | 1 | 0 | 0 | 3 |
| (157) | 1 | 0 | 1 | 0 | 0 | 0 | 1 | 1 |
| (158) | 9 | / | 1 | / | / | / | / | / |
| (159) | 6 | 4 | 4 | 0 | 3 | 4 | 4 | / |
| (160) | 2 | 0 | 0 | 0 | 0 | 2 | 2 | / |
| (161) | 29 | 8 | 3 | 0 | 5 | 20 | 3 | 17 |
| (162) | 29 | 24 | 13 | 2 | 2 | 15 | 29 | / |
| (163) | 58 | 24 | 10 | 0 | 25 | 42 | 6 | 0 |
| (164) | 11 | 5 | 3 | 5 | 5 | 8 | 10 | 2 |
| (165) | 4 | 2 | 0 | 0 | 0 | 2 | 3 | 0 |
| (166) | 43 | / | 0 | / | / | / | / | / |
| (167) | 23 | 17 | 13 | 0 | 8 | 12 | 3 | 16 |
| (168) | 2 | 1 | 0 | 0 | 0 | 1 | 0 | / |
| (169) | 5 | 0 | 0 | 4 | 4 | 4 | 2 | 0 |
| (170) | 9 | 7 | 7 | 0 | 6 | / | / | / |
| (171) | 8 | 2 | 2 | 0 | 1 | 1 | 0 | 8 |
| (172) | 3 | / | 3 | 0 | 2 | / | / | / |
| (173) | 33 | 10 | 6 | 11 | 11 | 28 | 31 | / |
| (174) | 14 | 1 | 0 | / | 0 | / | / | / |
| (175) | 20 | 12 | 5 | / | / | 11 | / | / |
| (176) | 7 | 4 | 1 | 0 | 4 | 5 | 3 | 0 |
| (177) | 31 | 16 | 12 | 0 | 0 | 15 | 21 | / |
| (178) | 9 | 4 | 5 | 0 | 6 | 7 | 2 | 3 |
| (179) | 5 | 4 | 2 | 3 | 3 | 3 | 3 | 5 |
| (180) | 3 | 0 | 0 | 0 | 0 | 1 | 3 | 3 |
| (181) | 12 | 10 | 7 | 0 | 0 | 5 | 2 | 8 |
| (182) | 11 | 8 | 5 | 0 | 3 | 7 | 2 | 9 |
| (183) | 7 | 5 | 2 | 0 | 0 | 3 | 5 | 4 |
| (184) | 2 | 2 | 2 | 0 | 2 | 2 | 2 | 1 |
| (185) | 10 | 6 | 6 | 0 | 8 | 8 | 10 | / |
| (186) | 15 | 3 | 3 | 3 | 5 | 10 | 12 | 12 |
| (187) | 12 | 1 | 0 | 0 | 5 | 4 | 0 | 12 |
| (188) | 11 | 5 | 2 | 4 | 4 | 8 | 8 | / |
| **Total** | **2,888** | / | / | / | / | / | / | / |

**Table 7 Footnote:**

1. detection bias for those delivering the intervention
2. performance and detection biases analysed jointly

- Not applicable

/ Not reported

**Supplementary Table 8.** **GRADE for the review’s main comparison**

| **Reference** | **Limitations of studies (risk of bias)** | **Inconsistency of results (heterogeneity, *I^2^*)** | **Indirectness of the evidence** | **Imprecision (sample size)** | **Publication bias** | **Comment** |
| --- | --- | --- | --- | --- | --- | --- |
| (39) | Yes | No | No | Yes | / | The quality of the evidence was low |
| (40) | Yes | Yes (I^2^ range: 78-96%) | Yes | Yes | No | Publication bias assessed with comprehensiveness of the search strategy, industry influence, funnel plot asymmetry and discrepancies between published and unpublished trials |
| (41) | / | / | / | / | / | The evidence was not graded; all but one of the included studies were at high risk of bias |
| (42) | Yes | No | No | Yes | No | The quality of the evidence was moderate for the main comparison |
| (43) | Yes | No | No | No | Yes | Egger test was asymmetrical for myocardial infraction (P=0.009) and hospitalisation admission (P=0.001) |
| (44) | / | / | / | / | / | The evidence was not formally graded |
| (45) | Yes | No | No | / | / | Downgraded by three levels for study limitations |
| (46) | / | / | / | / | / | Five trials were of ‘medium quality’ and one was of poor. The authors used the ’criteria list for the methodological quality criteria by van Tulder 1997 |
| (47) | Yes | No | No | No | No | Confidence intervals were wide but not downgraded for imprecision |
| (48) | Yes | No | No | Yes | No | The effect size was small for asthma symptoms |
| (49) | Yes | No | No | Yes | No | The quality of the included evidence was moderate for deaths, physical functioning, and fatigue, low for overall quality of life, depression, anxiety, AEs and serious AEs, and very low for physical performance |
| (50) | Yes | Yes | No | Yes | No | The quality of the evidence was considered to be low or moderate |
| (51) | Yes | No | No | Yes | No | Imprecision was serious and study limitations very serious |
| (52) | Yes | No | Yes | Yes | No | There was high heterogeneity of > 50% for stiffness |
| (53) | Yes | Yes | Yes | Yes | No | The evidence ranged from moderate to very low (predominantly low) |
| (54) | Yes | Yes (considerable heterogeneity, *I*² = 80%) | No | No (wide CIs were stressed) | No | The quality of the evidence was considered to be very low for all outcomes |
| (55) | Yes | No | Yes | Yes | No | Indirectness and imprecision were very serious (-2) |
| (56) | Yes | No | Yes | Yes | No | Indirectness rated as very serious (-2) |
| (57) | Yes | No | Yes | Yes | No | As above |
| (58) | Yes | No | No | Yes | No | Sample sizes and  wide CIs |
| (59) | / | / | / | / | / | van Tudler and Jadad scales were used to measure internal validity |
| (60) | Yes | Yes | No | Yes | No | The quality of the evidence was low for all outcomes |
| (61) | Yes | Yes | Yes | Yes | Yes | Considerable heterogeneity (*I*²=76%); very serious imprecision and indirectness |
| (62) | Yes | Yes | No | Yes | No | Heterogeneity range: (*I*²=45-96%) |
| (63) | Yes | No | No | Yes | No | Unclear how the GRADE system was used |
| (64) | Yes | No | No | Yes | No | *I*² = 93% precluded pooling for post-intervention lung function |
| (65) | Yes | No | No | Yes | No | All studies had a high risk of performance bias; and were of small sample sizes |
| (66) | Yes | No | No | No | No | GRADE system was not formally used |
| (67) | Yes | No | No | Yes | No | Very low-quality evidence (limitations in design, serious inconsistency and imprecision) for additional comparison |
| (68) | Yes | No | Yes | Yes | No | Very serious study limitations |
| (69) | Yes | Yes | No | Yes | No | The quality of the evidence was low or very low for all outcomes |
| (70) | Yes | Yes | No | No | Yes | Moderate heterogeneity (*I*² = 63%); publication bias (Begg P value= 0.02, Egger P value=0.002 |
| (71) | Yes | Yes (range: 0-87%) | Yes | Yes | Yes | The quality of the evidence was not officially GRADED |
| (72) | Yes | Yes (range: 0-95%) | No | Yes | No | Imprecision was main reason for downgrading |
| (73) | Yes | Yes | No | Yes | No | The GRADE system was not applied |
| (74) | Yes | No | Yes | Yes | No | Study limitations and imprecision were main reasons for downgrading |
| (75) | No | Yes | Yes | Yes | No | Very serious imprecision |
| (76) | Yes | Yes | No | Yes | No | Heterogeneity range: 30-61%; small samples |
| (77) | Yes | Yes | Yes | No | No | The grading system recommended by Tugwell 2004 was used |
| (78) | Yes | Yes | No | No | / | The quality of the evidence was low for all outcomes |
| (79) | Yes | No | No | Yes | No | Imprecision and study limitations were the main reasons for downgrading |
| (80) | / | / | / | / | / | Categories described by Clarke 2001were used |
| (81) | Yes | No | No | Yes | No | I^2^ =44% |
| (82) | Yes | No | No | Yes | No | Very serious imprecision |
| (83) | Yes | Yes (range: 77-80%) | Yes | Yes | Yes | The quality of the evidence ranged from very low to moderate |
| (84) | No | Yes (68%) | No | No | No | Downgraded for one outcome only: physical function |
| (85) | No | No | Yes | Yes | Yes | The authors did not attempt to retrieve unpublished studies; I^2^: range 23-41% |
| (86) | Yes | Yes (82%) | No | No | No | The quality of the evidence was very low |
| (87) | Yes | Yes | No | Yes | No | *I*^2^ range: 49-59% |
| (88) | Yes | No | No | Yes | No | Imprecision and study limitations were main reasons for downgrading |
| (89) | / | / | / | / | / | GRADE system was not applied |
| (90) | / | / | / | / | / | Inconsistency in reporting; high risk of bias; the possibility of publication bias was speculated |
| (91) | Yes | Yes | No | Yes | / | Heterogeneity ranged from 9-42%; detection and performance biases were the most common limitations |
| (92) | Yes | No | Yes | Yes | No | All studies had methodological limitations |
| (93) | Yes | Yes | Yes | Yes | / | Very serious inconsistency and risk of bias |
| (94) | Yes | No | No | Yes | / | All studies had a high risk of performance and detection bias |
| (95) | Yes | Yes | No | Yes | Yes | Imprecision was very serious |
| (96) | / | / | / | / | / | GRADE system was not applied |
| (97) | / | / | / | / | / | The overall risk of bias was judged to be moderate |
| (98) | / | / | / | / | / | High risk of bias and a high degree of heterogeneity were of concern |
| (99) | Yes | Yes (range 75-98%) | No | Yes | / | Funnel plots were not inspected |
| (100) | / | / | / | / | / | GRADE system was not formally employed; trials “were at significant risk of bias” |
| (101) | Yes | Yes | No | Yes | No | *I*^2^ range: 0-64% |
| (102) | / | / | / | / | / | van Tulder criteria for methodological quality were used |
| (103) | / | / | / | / | / | GRADE system was not formally employed; heterogeneity was small in majority of analyses |
| (104) | Yes | Yes | Yes | Yes | Yes | *I*^2^=73% for the main comparison and outcome |
| (105) | / | / | / | / | / | The checklists by Jadad 1996; Moher 1998 and Schulz 1995 were used |
| (106) | Yes | No | Yes | Yes | Yes | Very serious imprecision and study limitations |
| (107) | Yes | No | No | Yes | Yes | Small number of studies; and overall sample size |
| (108) | / | / | / | / | / | Low internal validity |
| (109) | Yes | Yes | No | Yes | No | Assessor blinding was ensured in two of the 16 studies |
| (110) | / | / | / | / | / | Only 24 studies had more than 100 participants at entry |
| (111) | Yes | Yes | No | No | No | *I*^2^ =69% |
| (112) | No | No | No | Yes | No | The evidence was not downgraded despite limitations of studies |
| (113) | Yes | Yes | No | No | No | Heterogeneity range: *I*^2^ = 47-75% |
| (114) | Yes | No | No | Yes | No | Only one poorly reported study was included |
| (115) | Yes | Yes | No | Yes | Yes | Heterogeneity range: *I*^2^ = 0 to 59% |
| (116) | Yes | Yes | Yes | Yes | Yes | Heterogeneity range: *I*^2^ =0 to 81%; publication bias coefficient 2.98, 95% CI 0.68 to 5.28 |
| (117) | / | / | / | / | / | GRADE system was not formally employed |
| (118) | Yes | Yes | No | Yes | Yes | Heterogeneity range: *I*^2^ = 50-90%; Egger’s test (P = 0.06 and 0.07) |
| (119) | No | Yes | No | Yes | Yes | Heterogeneity range: *I*^2^ = 64-89%; very serious imprecision |
| (120) | Yes | Yes | No | Yes | No | Heterogeneity range: *I*^2^ = 0-85% |
| (121) | / | Yes (Range: 54.9 to 91.4%) | / | / | / | GRADE system was not formally employed |
| (122) | No | Yes (Range: 52 to 76%) | No | Yes | No | Good methodological quality of the studies |
| (123) | Yes | No | No | Yes | No | Low internal and external validity |
| (124) | Yes | Range: 0 to 41% | No | No | No | GRADE system was not formally employed |
| (125) | Yes | Yes (Range: 0 to 90%) | No | No | See comment | […] low quality trials, usually small studies, that comprise the majority of the studies in the review probably overestimate the effect of resistance training” |
| (126) | Yes | No | Yes | Yes | No | Predominantly very low-quality evidence |
| (127) | Yes | Yes | Yes | Yes | No | Very serious indirectness and study limitations |
| (128) | Yes | Yes | No | Yes | No | Very serious imprecision |
| (129) | Yes | No | No | Yes | No | Low quality evidence; imprecise findings |
| (130) | Yes | Yes (Range: 0 to 68%) | No | Yes | No | Very serious study limitations under inhibition control |
| (131) | Yes | No | No | Yes | No | Studies’ limitations were the main reasons for downgrading |
| (132) | Yes | Yes (Range: 0 to 69%) | No | No | No | The evidence not downgraded for studies limitations despite high risk of bias |
| (133) | / | / | / | / | / | Low generalizability |
| (134) | Yes | Yes (Range: 0 to 89%) | No | Yes | Yes | Large number of trials and participants for majority of the outcomes |
| (135) | Yes | Yes (Range: 76 to 85%) | Yes | Yes | No | Very serious imprecision |
| (136) | / | / | / | / | / | No blinding of outcome assessment, and 12% of participants were excluded from the analysis |
| (137) | Yes | Yes (Range: 0 to 81%) | / | / | / | Significant methodological heterogeneity |
| (138) | / | / | / | / | / | GRADE system was not formally employed; clinical and methodological heterogeneity |
| (139) | Yes | Yes (Range: 0 to 55%) | No | Yes | No | Upgraded evidence due to a dose-response gradient under walking endurance |
| (140) | Yes | Yes | No | Yes | Yes | Moderate to high statistical heterogeneity |
| (141) | Yes | Yes | No | Yes | Yes | Downgraded for imprecision under 5 outcomes |
| (142) | / | / | / | / | / | GRADE system was not formally employed; all except inconsistency and indirectness apply |
| (143) | Yes | No | Yes | Yes | No | Not downgraded for inconsistency despite *I*^2^=64% |
| (144) | Yes | No | No | Yes | No | Not downgraded for inconsistency despite *I*^2^=52%; and study limitations despite high risk of bias |
| (145) | Yes | Yes (Range: 30 to 60%) | No | Yes | No | Three outcomes under main comparison were based on one RCT |
| (146) | / | / | / | / | / | GRADE system was not formally employed |
| (147) | / | / | / | / | / | Reduced generalizability |
| (148) | Yes | Yes | No | Yes | Yes | GRADE system was not formally employed; heterogeneity range: 0 to 100% |
| (149) | Yes | No | No | Yes | No | Heterogeneity range: 0 to 59% but not downgraded for inconsistency |
| (150) | Yes | No | No | Yes | No | Effect estimates based on one study; very imprecise |
| (151) | No | No | No | No | No | Not downgraded for imprecision despite one small trial contributing |
| (152) | Yes | No | No | Yes | No | Study limitations and imprecision were main reasons for downgrading |
| (153) | Yes | No | No | Yes | No | Wide confidence intervals and lack of blinding |
| (154) | Yes | Yes | No | Yes | No | Mainly study limitations and imprecision |
| (155) | Yes | No | No | Yes | No | Indirectness and imprecision were of main concern |
| (156) | Yes | Yes | No | Yes | No | Very serious risk of bias, heterogeneity range: 0-49% |
| (157) | Yes | No | No | No | No | Moderate quality evidence despite one biased study being evaluated |
| (158) | / | / | / | / | / | The methodological quality score ranged from 55% to 82% of the maximum feasible score of 22 points (van Tulder 2003) |
| (159) | Yes | Yes | No | Yes | No | Very serious risk of bias; serious inconsistency for QOL physical component |
| (160) | Yes | No | No | Yes | No | Very serious risk of bias; only two trials with small total sample size |
| (161) | Yes | Yes | Yes | Yes | Yes | Overall high risk of bias for all trials and imprecision (sample size <400) |
| (162) | Yes | Yes | No | Yes | Yes | Inconsistency and  imprecision were main reasons for downgrading |
| (163) | Yes | Yes | No | No | No | Mainly high-quality evidence; not downgraded despite studies limitations |
| (164) | / | / | / | / | / | Methodological quality and reporting were variable |
| (165) | Yes | / | / | / | / | Very serious study limitations |
| (166) | Yes | / | / | / | No | All studies had some methodological weaknesses |
| (167) | Yes | No | No | Yes | No | *I*² = 42%; high risk of performance bias in all studies |
| (168) | Yes | No | No | No | No | Limited generalisability of the findings |
| (169) | Yes | Yes | No | Yes | No | *I*² = 43-54% |
| (170) | No | No | No | Yes | No | *I*^2^<50% |
| (171) | Yes | Yes | No | Yes | No | Imprecision and risk of bias were main reasons for downgrading |
| (172) | / | / | / | / | / | Heterogeneity of interventions and outcome measures was a limiting factor |
| (173) | Yes | Yes (Range: 0-79%) | No | Yes | Yes | Only 5 (15%) studies were at a high risk of bias |
| (174) | Yes | Yes (Range: 0-76%) | No | Yes | Yes | Assessor blinding not reported in any of the trials |
| (175) | Yes | / | / | / | / | Only one study was judged to be at low risk of bias across all domains assessed |
| (176) | Yes | Yes (Range: 0-73%) | No | Yes | No | Evidence not downgraded despite inclusion of quasi RCT |
| (177) | Yes | Yes (Range 74 to 94%) | Yes | Yes | No | Very serious inconsistency and risk of bias (quasi RCTs) |
| (178) | Yes | Yes | No | Yes | Yes | Reasons for downgrading not provided |
| (179) | No | No | No | Yes | No | Difference not clinically important |
| (180) | Yes | No | No | No | No | Very serious limitations |
| (181) | Yes | Yes (I^2^ ≥75%) | No | Yes | Yes | Imprecision (<400 participants) |
| (182) | Yes | Yes | Yes | Yes | No | *I*² = 74-82% |
| (183) | Yes | Yes (Range: 0 to 84%) | No | Yes | No | Very serious study limitations and imprecision |
| (184) | Yes | No | No | Yes | No | Very serious imprecision (N=37); *I*^2^=47% |
| (185) | Yes | Yes (Range: 0 to 56%) | No | Yes | Yes | Study limitation – main reasons for downgrading |
| (186) | Yes | Yes (*I*^2^= 68%) | No | Yes | Yes | Indirectness was not considered significant |
| (187) | Yes | Yes (Range: 0 to 80%) | No | No | No | / |
| (188) | Yes | No | No | Yes | No | Sample size <400 for all outcomes |
| **Total (yes)** | **114** | **68** | **27** | **98** | **26** | **/** |

**Table 8 Footnote:**

/ = Not reported or not estimable

**Supplementary Table 9: Studies reporting quality of life outcomes as mean difference**

| **Outcome_name** | **Author** | **Year** | **Disease** | **Exercise** | **Duration** | **Session length** | **Frequency per week** | **Evidence quality** | **ROB** | **Total studies** | **No patients** | **MD** | **LL** | **UL** |
| --- | --- | --- | --- | --- | --- | --- | --- | --- | --- | --- | --- | --- | --- | --- |
| Health-related QoL | Bidonde | 2017 | Fibromyalgia | aerobic exercises | 15 weeks | 35 mins | 2-3 times | Moderate | High | 13 | 839 | -7.89 | -13.23 | -2.25 |
| Health-related QoL | Bidonde | 2017 | Fibromyalgia | vibration exercise | NR | NR | NR | Very low | High | 4 | 150 | -3.73 | -10.81 | 3.35 |
| Health-related QoL | Broderick | 2017 | Schizophrenia | yoga | 2-12 weeks | NR | 3-5 times | Low | High | 6 | 586 | -5.3 | -17.78 | 7.78 |
| Overall QoL | Broderick | 2015 | Schizophrenia | yoga | 2-12 weeks | NR | 3-5 times | Low | High | 8 | 457 | 15.5 | 4.27 | 26.73 |
| Overall QoL | Broderick | 2017 | Schizophrenia | yoga | 3-8 weeks | NR | 3-5 times | Low | High | 3 | 193 | 22.93 | 19.74 | 26.12 |
| Overall QoL | Freitas | 2013 | Asthma | Breathing exercises | 2-16 weeks | 10-75 mins | 3 times per day-3 times per week | Very low | Unclear - high | 13 | 906 | 0.79 | 0.5 | 1.08 |
| Overall QoL | Furmaniak | 2016 | Breast cancer | Aerobic or resistance exercise interventions | NR | NR | NR | Low | High | 32 | 2626 | 1.1 | -5.28 | 7.48 |
| Health-related QoL | Gorczynski | 2010 | Schizophrenia or related conditions | Walking or resistance exercise and aerobic training | 12-16 weeks | NR | NR | Very low | High | 3 | 86 | -9.22 | -18.86 | 0.42 |
| Overall QoL | Holland | 2012 | Chronic obstructive pulmonary disease | Breathing exercises | 1 session-20 months | NR | 0-3 times | Low | High | 16 | 1233 | -12.94 | -22.29 | -3.6 |
| Health-related QoL | Hurley | 2018 | Chronic knee or hip joint pain from OA | Multiple exercises | 4-24 weeks | 20-60 mins | 0.5-5 times | Low | High | 21 | 2372 | 58.3 | 34.58 | 82.02 |
| Health-related QoL | Lane | 2017 | atherosclerotic disease | resistance training to pole striding and upper or lower limb exercises | 14 days - 2 years | 15-90 mins | 2 per day to 2 per week | Moderate | Low -unclear | 32 | 1835 | 2.15 | 1.26 | 3.04 |
| Health-related QoL | Lane | 2017 | atherosclerotic disease | resistance training to pole striding and upper or lower limb exercises | 15 days - 2 years | 15-90 mins | 3 per day to 2 per week | Moderate | Low -unclear | 32 | 1835 | 3.76 | 2.7 | 4.82 |
| Health-related QoL | Lawrence | 2017 | Stroke survivors | yoga | 8-10 weeks | 40-90 mins | ≥3 times | Very low | High | 2 | 72 | 15.3 | 1.29 | 29.31 |
| Health-related QoL | Loughney | 2018 | Cancer | Multiple exercises | 5 weeks - 12 months | 15 - 90 mins | 2 - 6 times | Moderate | High | 11 | 1067 | 2.29 | -1.06 | 5.65 |
| Overall QoL | Meekums | 2015 | Depression | Dance movement therapy | 4-12 weeks | 45 mins - 2 hours | 2 - 5 times | Low | High | 3 | 147 | 0.3 | -0.6 | 1.2 |
| Health-related QoL | Morris | 2017 | Pulmonary hypertension | upper and lower limb exercise | 9 - 33 weeks | 30 - 60 mins | 2 - 7 times | Low | High | 65 | 11444 | 4.63 | 0.8 | 8.47 |
| Health-related QoL | Morris | 2017 | Pulmonary hypertension | upper and lower limb exercise | 9 - 33 weeks | 30 - 60 mins | 3 - 7 times | Low | High | 65 | 11444 | 4.17 | 0.01 | 8.34 |
| Overall QoL | Østerås | 2017 | Hand OA | NR | 6 weeks - 12 months | NR | 4 times daily - 2 times per week | Very low | High | 7 | 534 | 0.3 | -3.72 | 4.32 |
| Overall QoL | Regnaux | 2015 | Knee or hip OA | resistnace training | 8-24 weeks | 25-60 min | 2 - 5 times | Very low | High | 6 | 656 | 4.3 | -6.5 | 15.2 |
| Health-related QoL | Takken | 2008 | Juvenile idiopathic arthritis | Multiple exercises | 10 weeks - 6 months | 60 mins | 1-3 times | Low | Low | 3 | 212 | -3.96 | -8.91 | 1.00 |
| Health-related QoL | Taylor | 2014 | Heart failure | Aerobic exercise + resistnace trainin g | 15 - 120 weeks | 15- 120 mins | 1 - 7 times | Low | Low-unclear | 33 | 4740 | -5.8 | -9.2 | -2.4 |
| Overall QoL | Yang | 2016 | Asthma | yoga | 2 weeks - 54 months | 15 -180 mins | 2 per day to 2 per week | Moderate | High | 15 | 1048 | 0.57 | 0.37 | 0.77 |
| Health-related QoL | Zainuldin | 2011 | Chronic obstructive pulmonary disease | Leg exercise training | 3 - 16 weeks | 27 - 72 mins | 2 - 5 times | Moderate | Unclear | 11 | 598 | -1.26 | -0.01 | 2.54 |

**Table 9 Footnote:** LL = lower limit; MD = mean difference; ROB = risk of bias; UL = upper limit.
